# Progression of Diabetic Capillary Occlusion: A Model

**DOI:** 10.1371/journal.pcbi.1004932

**Published:** 2016-06-14

**Authors:** Xiao Fu, John Scott Gens, James A. Glazier, Stephen A. Burns, Thomas J. Gast

**Affiliations:** 1 The Biocomplexity Institute, Indiana University, Bloomington, Indiana, United States of America; 2 Department of Physics, Indiana University, Bloomington, Indiana, United States of America; 3 Department of Intelligent Systems Engineering, Indiana University, Bloomington, Indiana, United States of America; 4 School of Optometry, Indiana University, Bloomington, Indiana, United States of America; University of Virginia, UNITED STATES

## Abstract

An explanatory computational model is developed of the contiguous areas of retinal capillary loss which play a large role in diabetic maculapathy and diabetic retinal neovascularization. Strictly random leukocyte mediated capillary occlusion cannot explain the occurrence of large contiguous areas of retinal ischemia. Therefore occlusion of an individual capillary must increase the probability of occlusion of surrounding capillaries. A retinal perifoveal vascular sector as well as a peripheral retinal capillary network and a deleted hexagonal capillary network are modelled using Compucell3D. The perifoveal modelling produces a pattern of spreading capillary loss with associated macular edema. In the peripheral network, spreading ischemia results from the progressive loss of the ladder capillaries which connect peripheral arterioles and venules. System blood flow was elevated in the macular model before a later reduction in flow in cases with progression of capillary occlusions. Simulations differing only in initial vascular network structures but with identical dynamics for oxygen, growth factors and vascular occlusions, replicate key clinical observations of ischemia and macular edema in the posterior pole and ischemia in the retinal periphery. The simulation results also seem consistent with quantitative data on macular blood flow and qualitative data on venous oxygenation. One computational model applied to distinct capillary networks in different retinal regions yielded results comparable to clinical observations in those regions.

## Author Summary

Diabetes is a disease of elevated blood sugar which damages the body’s blood vessels, especially in the eye. Current understanding is that diabetics block one capillary at a time basically because white blood cells become ‘sticky’ in diabetes. This begins a process which results in blockage of a vessel and starves a retinal area of oxygen. This process is believed to randomly block vessels but patients actually have large areas with no intact vessels. We developed a computer model of this process which shows why diabetics have areas in which all capillaries are occluded rather than blocked randomly. Areas develop because the factors released from the retina around one blocked vessel increase the chance that nearby capillaries also become blocked. This understanding may increase our ability to intervene in the process to prevent these large ischemic areas from developing and thereby prevent blindness from diabetic retinopathy.

## Introduction

**Fig 1:**
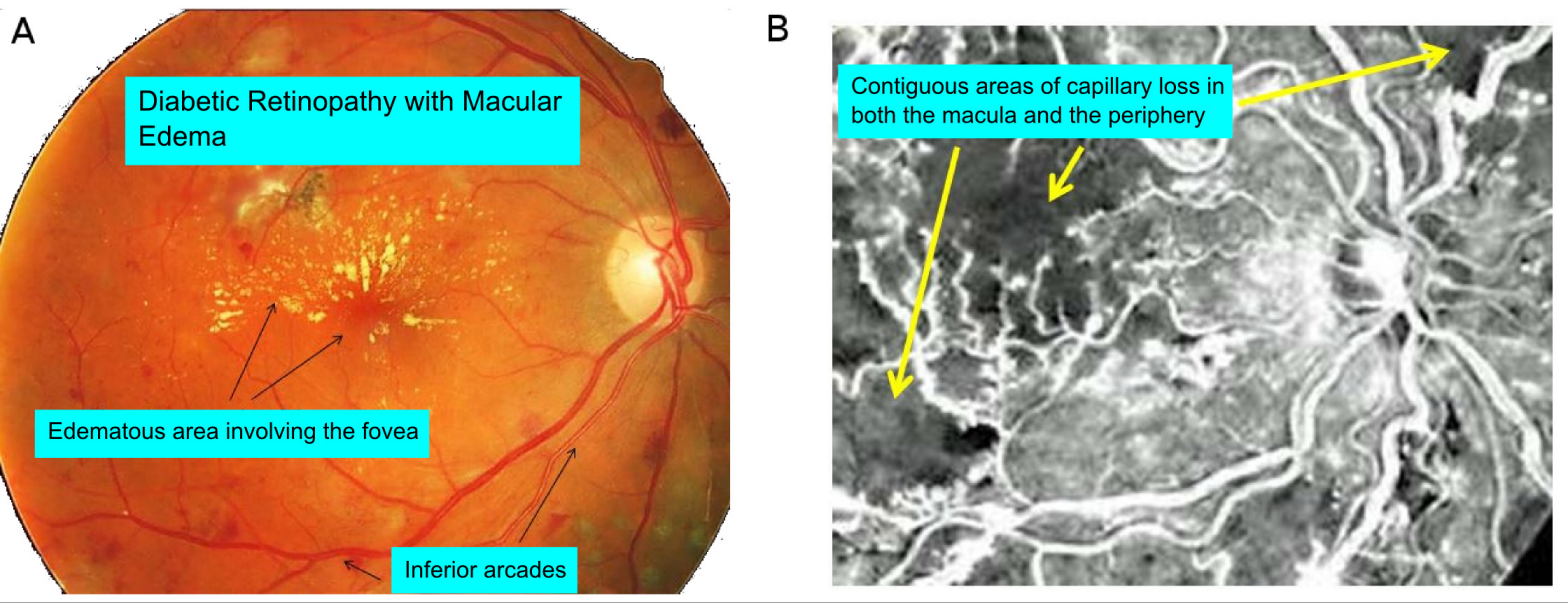
Clinical background of diabetic retinopathy. (A) Fundus image of patient with a 10 year history of type 2 diabetes mellitus showing hard exudates in the macula indicating edema [15]. Dot/blot hemorrhages, and some laser burns are also present. Image is taken with a standard color fundus camera. Resolution is adequate to see arterioles and venules but not the capillaries connecting them. For perspective the disk diameter is approximately 1450 microns. (B) Fluorescein angiogram of the diabetic fundus. The image shows areas that are ‘dark’ and therefore lack patent capillaries. These occur in various areas of the image. Certain vessels appear to lack sharpness because they show some leakage of the fluorescein dye through their walls; walls that would not leak if the normal blood retinal barrier were intact. This image shows the phenomenon this paper is primarily addressing, the occurrence of contiguous areas of capillary loss both in the macula and in the periphery in diabetic retinopathy.

Diabetes mellitus is a group of metabolic diseases characterized by hyperglycemia, or elevated blood glucose. Diabetes is a major and increasingly common problem both in the United States and globally. The global prevalence of diabetes is estimated to be 9%, and diabetes causes approximately 1.5 million deaths per year [1, 2]. By 2030, diabetes mellitus is projected to be the 7^th^ leading cause of death worldwide [3]. Either defects in insulin secretion, type 1 diabetes, or in the action of insulin, type 2 diabetes, may cause the hyperglycemia which over time results in damage and dysfunction to many organs. Type 2 diabetes is the most common form of diabetes and includes 90% of people with diabetes worldwide. Both types of diabetes produce similar complications [4]. Diabetes causes its morbidity and mortality through both macrovascular and microvascular damage. The macrovascular complications are cardiovascular disease, stroke, and peripheral vascular disease [5–9]. The commonly accepted microvascular complications are diabetic retinopathy, nephropathy, and peripheral neuropathy of which the most common is diabetic retinopathy. Diabetes causes 1% of worldwide blindness and diabetic retinopathy is the leading cause of blindness in people 20–64 years of age in the U.S. [4, 10]. Diabetic retinopathy is defined as damage to the retina as a complication of diabetes [4]. Its early clinically visible manifestations are damage to the smallest blood vessels, the capillaries, in the retina with resultant microaneurysms, hemorrhages, edema, and nerve fiber layer infarcts [4]. The major cause of decreased visual acuity is diabetic maculopathy which reduces visual acuity largely as a consequence of macular edema, fluid accumulation in the macula due to leakage from abnormal capillaries (Fig 1A). A fraction of patients lose visual acuity solely due to macular ischemia, a loss of blood flow in most of the perifoveal capillaries. Most affected patients will have areas of abnormal leaking capillaries with surrounding edema and adjacent areas of capillary occlusion often seen as enlargement of the foveal avascular zone (FAZ) [11–14].

The major cause of blinding diabetic retinopathy is the development of abnormal new blood vessels either occurring at the optic nerve head as a sign of global ischemia or elsewhere at the border of a large ischemic area. This neovascularization is termed proliferative diabetic retinopathy. Blindness often results from hemorrhage filling the vitreous with opaque blood and initiating a damaging fibrovascular scarring process [16]. Examination of the retinal vasculature with fluorescein angiography and more recently through other techniques such as adaptive optics scanning laser ophthalmoscopy (AOSLO) commonly shows patterns of capillary loss in both the macula and especially in the midperiphery that are striking in their contiguous nature (Fig 1B). Large patches of retina appear dark on fluorescein angiography i.e. lacking in patent, or unobstructed, capillaries (Figures 6-21-3, 6-21-4 in [16] Figures 11.3b, 11.6 in [17], Figures 6.1 and 6.2 in [18], Figures 4–122, 123,126 in [19], Figures 5–14,5–16 in [20], Figure 1 and 3 in [21]). These dark areas tend to be noted as “peripheral ischemia or non-perfused areas” without further elaboration. The severe loss of vision in diabetes from neovascularization is related to these dark areas of peripheral retinal ischemia. Treatment of retinal neovascularization involves laser panretinal photocoagulation which destroys an oxygen sink, the photoreceptors, and allows oxygen influx from a source, the choriocapillaris [22]. Thus ischemic retina becomes oxygenated and ceases production of excess vascular endothelial growth factor (VEGF) [22, 23]. Additionally retina on one side of a bounding retinal arteriole or venule may be completely lacking in capillaries whereas that on the other side sometimes possesses an intact capillary network (Fig 1B). This sort of pattern occurs both in the peripheral retina and also between the arterioles and venules supplying the fovea (Figure 6.16 P 136, [24]).

The complications of diabetes in the eye are therefore largely related to physiological disturbances of these smallest retinal vessels in the form of confluent capillary occlusion with resultant retinal ischemia leading to elevated production of VEGF. The elevated VEGF leads to capillary leakage which in turn results in retinal edema, and if sufficient areas of retina are involved, to neovascularization. In the periphery large ischemic areas usually without significant edema are seen whereas posteriorly the ischemic areas are generally accompanied by hyperemia and retinal edema [25–28]. These clinical findings are common if not universal and can be seen within classifications of diabetic capillary non-perfusion [29].

The fundamental pathological process resulting in individual capillary occlusion is believed to be the result of an activated leukocyte adhering to and damaging the retinal capillary wall composed of endothelial cells. This local endothelial cell loss ultimately results in occlusion of a single capillary. The significant question we address in the current paper is how random capillary occlusion by leukocytes can lead to the large clinically observed areas of confluent retinal ischemia.

For random capillary occlusion to lead to the formation of a large area in which all capillaries are occluded, the occlusion of an individual capillary must increase the probability of occlusion of nearby capillaries. That is, while the initial occlusion may be random, the probability of nearby occlusions must be non-random, such that occlusion begets further occlusion in the form of an adverse feedback cycle. This paper creates a physiologically sound computational model which ties these biologically observed random occlusions of capillaries by leukocytes to the clinically manifested patterns of occlusions that indicate an eye at risk of blindness. This is achieved by relating local capillary occlusion to local vascular endothelial growth factor (VEGF) levels and further capillary loss. An essential feature for the model is that with capillary occlusion local retinal tissue becomes ischemic and that ischemic tissue releases a factor or factors which increase the likelihood of nearby capillary occlusion producing the adverse feedback mechanism which can propagate further capillary occlusions.

In more detail: elevated blood glucose creates a permissive state because an elevated glucose causes a slight elevation of VEGF due to direct consequences of glucose on the molecular biology of VEGF. This then causes a slight elevation of retinal capillary intercellular adhesion molecules (ICAMs). The elevated level of ICAM-1 then increases the rate of binding of activated leukocytes to capillary endothelial cells. This activated leukocyte, in turn, can kill the endothelial cell it binds to. Thus the permissive state of the retina results in the continual loss of randomly located retinal vascular endothelial cells due to increased binding of activated leukocytes over the non-diabetic state. The loss of endothelial cells over time eventually exhausts endothelial replicative capacity and results in capillary occlusion. Occlusion of a capillary causes the local area of retina primarily supplied by that capillary to become hypoxic. In this hypoxic area of retina there is an exponential rise of VEGF synthesis. This VEGF diffuses, increasing the VEGF level that surrounding still patent capillaries experience. Increased VEGF induces further elevation of ICAM-1 levels in these surrounding capillaries. Elevated ICAM-1 again increases the probability of capillary occlusion. If this feedback is sufficiently strong, each random capillary occlusion will initiate an expanding area of capillary loss, due to increased binding of leukocytes and consequential accelerated endothelial cell loss and capillary occlusion in these adjacent areas. This ischemic area will then increase in size until it reaches the oxygenated boundary of a large retinal arteriole or venule.

While we posit the model is based on VEGF, the identification of VEGF as the critical substance released by ischemic tissue need not be specifically or solely VEGF and likely is a balance of factors. It is also not critical that it be released by one particular cell type. It seems essential that the process of ischemic damage does not reduce the production of the critical diffusing substance, modelled as VEGF, through death of the synthesizing cell, and thus the positive feedback cycle is able to remain intact.

### Support for the model’s physiological assumptions

VEGF is a factor with a long history. First identified as a vascular permeability factor [30, 31], it has become clear over time that it has other important roles as a factor in angiogenesis [32], endothelial cell proliferation [33], and also as a neuroretinal protective factor [34, 35]. While we do model retinal edema [36] the important property of VEGF for this model is its role a mediator of elevated ICAM-1 on retinal endothelial cells. In our model of progressive capillary occlusion, VEGF is the locally secreted molecule which diffuses and increases the likelihood of nearby capillary occlusion. As this pro-occlusive property is not as well-known and even possibly denied by some, we provide below the background supporting our choice of VEGF as a substance responsible for the adverse cycle of capillary occlusion. This does not mean VEGF is by any means the only cytokine involved or that there is not an intervening cascade of events that generate the chronic inflammatory state that is diabetic retinopathy. Leukostasis is mediated by the diabetic activation of circulating leukocytes co-existing with marked upregulation of adhesion molecules such as ICAM-1 on the retinal vascular endothelium [37]. These changes increase the likelihood of leukocyte adhesion to the retinal capillary endothelium and therefore the probability of capillary closure. The model does not detail the local, undoubtedly complex, phenomena such as the cumulative leukocyte mediated endothelial capillary damage resulting in endothelial replicative exhaustion culminating in capillary occlusion; they are treated as black boxes at this time.

The model treats elevation of one substance for simplicity though likely the relative amounts of two substances, such as the balance of VEGF and pigment epithelium derived factor (PEDF), is what is often physiologically important [38]. PEDF itself has complex neurotrophic, neuroprotective, and anti-angiogenic, anti-exudative and anti-inflammatory properties [39, 40]. High glucose decreases expression of PEDF in retinal Mueller cells as it simultaneously elevates VEGF expression. Additionally vitreous levels of PEDF are significantly lower in patients with diabetic macular edema or proliferative diabetic retinopathy than in non-diabetic patients or diabetic patients without retinopathy whereas in each situation VEGF is elevated. The model simplifies this duality by treating the physiological import of an imbalance as simply the concentration of VEGF. Certain steps in the progressive ischemic process must be met by a diffusible substance and ideally we need to have anatomical and physiological support for each of the steps in the model if we are to model the substance as VEGF. The local adverse positive feedback model could stand on its own dealing only with the problem of diabetic ischemia as a geographic phenomenon but it is more constructive to have model elements that correspond as closely as possibly to biological elements. We now construct a simple biological model based on the observation that this substance is required to have a number of physiological properties which first create the permissive diabetic state of recurrent capillary endothelial cell loss by activated leukocytes, leads to permanent capillary occlusions from local endothelial depletion, and which in turn produces geographic propagation of capillary occlusions. This progression requires a substance, modelled as VEGF, and the retinal tissue to have the following characteristics:

Step 1: With the onset of hyperglycemia or the continued presence of hyperglycemia this substance must change its production in some way from that of the non-diabetic state.
Step 2: The change in concentration of this substance must cause some change in the retinal capillaries resulting in an increased probability of permanent capillary occlusion above essentially zero in the non-diabetic state.
Step 3: Capillary occlusion must in turn create local retinal ischemia and by some mechanism further elevate the level of the substance, meaning that ischemia cannot act to kill the cells producing the substance.
Step 4: Higher concentrations of this substance in a local retinal area must cause a higher likelihood of occlusion in nearby capillaries with the probability of occlusion functionally related to the concentration of the substance.

#### Step 1: Initial slight elevation of VEGF in diabetes, above the basal level of a non-diabetic, to initiate a non-zero probability of activated leukocyte adhesion to the capillary endothelium

Step 1 requires an initial slight elevation of VEGF in the retina in response to an elevation of glucose to create a non-zero probability of capillary occlusion as a way to create an initial state allowing capillary endothelial cell destruction and ultimately capillary occlusion. Ideally the cellular source of the VEGF should also be specified. Different Retinal cell types respond to elevated glucose in a variety of ways. Retinal pigment epithelial (RPE) cells respond to acute elevation of glucose with an increased production of VEGF (as well as a decreased production of PEDF) [41]. Though these changes are in the directions required by the model, the secreted factors likely leave the basal portion of the RPE cells, below the posterior blood brain barrier, and move into the choriocapillaris not the neural retina [42]. In addition, the choriocapillaris rather than the retinal vasculature provides most of the oxygen to the RPE, so loss of retinal capillaries is unlikely to reduce oxygenation of the RPE significantly, a key step in our hypothesized feedback loop (steps 3 and 4). Our retinal model therefore neglects RPE cells as a source of VEGF (see Lutty [43] for the choroid).

There is evidence that both endothelial cells and pericytes [44] respond to an elevation of glucose with at least some VEGF production and elevation of ICAM-1 as well as NF-Kβ [45]. Pericytes are likely the earliest cells to die in diabetic retinopathy, and as they and endothelial cells die with the process of capillary occlusion, they are therefore essentially absent from the areas of peripheral ischemia. Therefore these two cell types are not likely the source of factors for the propagation of occlusion though they certainly have a role to play in the process, especially a possible role in initiation of elevated probability of occlusion through ICAM-1 induction [46] and also through hyperglycemia-induced angiopoietin 2 mediated apoptosis of pericytes [47]. Angiopoietin 2 is important in the loss of pericytes and therefore in microvascular diabetic complications. It is present in elevated concentrations in the vitreous in proliferative diabetic retinopathy and is produced in the retina. We concentrated on VEGF in the model because the physiology supports its relationship to ICAMs and capillary occlusion, the central point of the model, much more clearly than it does for angiopoietin 2 and more is known about it. Human retinal endothelial cells unlike endothelial cells in some animal models, when exposed to elevated glucose do not stimulate endogenous ROS production, activation of NF-Kβ, or other proinflammatory changes [47]. Other cells in the retina may produce VEGF but the only cell with significant evidence of increased VEGF secretion caused by elevated glucose, and with survival in ischemic retina, is the major glial cell of the retina, the Mueller cell [48–50]. Du *et al*. 2004 [51] show that Mueller cells exposed to elevated levels of glucose also produce iNOS, ICAM, cytokines, and PGE2. There is further support for these results and evidence for mediation by CaMKII-CREB in Jun Li *et al*. 2012 [52]. The degradation of HIF-1α is controlled by von Hippel-Lindhal suppressor protein and degradation is lessened at elevated glucose levels raising VEGF through HIF-1α [53]. Increasing levels of the transcription factor HIF-1α increases synthesis of VEGF above the base line of a non-diabetic. Diabetes is a chronic disease and clinically observable retinal pathology is generally not present until at least several years of the condition. This means that Advanced Glycation End products (AGEs) are present and could additionally serve a role in elevating synthesis of VEGF. Mamputu *et al*. [54] show this possibility with VEGF induction of ICAMs through AGEs in Mueller cells. Diabetes also elevates RAGE expression in Mueller cells [51]. Others have noted that although hypoxia stimulates the release of hypoxia regulated vasoproliferative factors, such as VEGF, VEGF has been found to be increased in the retinas of diabetic animals before capillary degeneration therefore indicating that factors other than hypoxia must regulate its induction in diabetes [55]. Additionally VEGF is present in the retina at basal levels prior to the initiation of diabetes and is increased significantly within days of the onset of diabetes [56]. Mueller cells survive in ischemic areas and as these areas increase in size, the total amount of VEGF synthesized would therefore increase. In conclusion, physiological support exists for a small increase in VEGF from Mueller cells in the diabetic retina prior to any ischemia. This is the permissive step in the model that discriminates the diabetic state from the non-diabetic one. This is supportive of the model only if this elevation of the substance (VEGF) above the basal state, step 1, is able to increase the probability of leukocyte adhesion to the retinal capillary endothelium.

#### Step 2: VEGF raises capillary endothelial ICAM increasing leukostasis and the probability of capillary occlusion

As early as 1991 [57] there was evidence of capillary occlusion secondary to activated granulocytes and monocytes in experimental diabetic retinopathy. VEGF was shown to increase expression of ICAM-1 in endothelial capillaries in vivo [58]. In human diabetic retina, ICAM increases adhesion of leukocytes and monocytes to the vascular endothelium [59]. Joussen *et al*. [60] showed that VEGF induces retinal ICAM-1 and eNOS expression and initiates early diabetic retinal leukocyte adhesion in vivo. VEGF is produced in Mueller cells of the retina, and inhibition of Mueller cell-derived VEGF significantly decreased retinal expression of TNFα, ICAM-1 and NF-Kβ in diabetic mice [50]. This supports VEGF being upstream to ICAM-1 and other pro-inflammatory substances including NF-Kβ. Nitric oxide as well as inflammatory proteins, including iNOS and ICAM, cytokines, and PGE2 are produced by Mueller cells exposed to elevated levels of glucose [51]. Diabetes has been shown to activate NF-Kβ in rodent retinas [61, 62] and to cause migration of the p65 subunit into nuclei of retinal endothelial cells, pericytes, ganglion cells, and cells of the inner nuclear layer (likely Mueller cells) [61, 63]. Activation of NF-Kβ results most commonly in the translocation of p50-p65 heterodimers into the nucleus, where subsequently transcription of a variety of proinflammatory proteins including iNOS, ICAM, and cytokines is induced. These papers [50, 51, 58–60] support the elevation of ICAM in the complex inflammatory state that is diabetic retinopathy and for tractability we deal in the model only with VEGF and its contributory role in ICAM induction. The process of capillary occlusion initiates when leukocytes adhere to the wall of a capillary. There are many factors related to leukocytes’ mechanical properties such as increased rigidity that also likely increase leukostasis but the dominant interpretation of capillary occlusion in diabetic retinopathy is that diabetic leukocytes are much more commonly activated than those in non-diabetics [64]. In this state they possess cell surface receptors, CD18, CD11a and CD11b that bind to ICAM-1 on the surface of retinal endothelial cells [60]. The activation of leukocytes can be induced by elevated glucose alone [64]. ICAM-1 is not normally present on endothelial cells but can be induced by VEGF or by other mechanisms such as an increased production of reactive oxygen species by oxidized LDL [65]. This leukostasis in the retinal capillaries occurs quite early, within 2 weeks of diabetes onset [66]. Thus there is strong support for VEGF promotion of ICAM expression on endothelial cells [55] and this results in increased probability of leukocyte adhesion to the endothelium [67] supporting part of the model’s step 2.

Leukocytes adhere to retinal vascular endothelium in diabetes [67] and likely are instrumental in the permanent occlusion of capillaries in diabetic retinopathy. The first demonstration that vascular endothelial damage occurs in diabetes was in Cogan *et al*. 1961 [68]. From correlative studies of retinal trypsin digests and fluorescein angiograms we know that as long as endothelial cells are present, capillaries are perfused and non-perfused capillaries are associated with damaged endothelial cells and empty basement membrane tubes [69]. It is unclear how leukocytes damage retinal endothelium as multiple overlapping mechanisms are involved. We have detailed elsewhere the presence of cell adhesion factors present on both the leukocytes in diabetes and on the retinal capillary endothelium. There are mechanical factors both involving the thickened basement membrane of the capillary wall, and the increased rigidity of leukocytes [70] in diabetes. Substances released by leukocytes, toxic oxygen metabolites and various enzymes, can cause significant ‘bystander’ damage. Attachment to endothelial cells strongly increases the ability of neutrophil’s to produce reactive oxygen metabolites [71]. Also, activated neutrophils from diabetic animals produce more superoxide radicals than those from non-diabetics [72], suggesting that leukocytes in the diabetic are both more adherent and more damaging to endothelium. Degranulated PMNs have also been observed in association with apparently dying endothelial cells [73]. As the neutrophil contains a number of types of granules including cationic lysosomal proteins which increase vascular permeability, acid and neutral proteases which digest basement membranes, and neutrophil elastase, it is reasonable that endothelial toxicity would result. Fas levels are increased in retinas of rats that were diabetic for 2 weeks, and blocking FasL in vivo inhibited endothelial cell damage, vascular leakage, and platelet accumulation [74]. This dependence on Fas/FasL shows the importance of apoptotic mechanisms on endothelial cell loss even in the physiological context of their exposure to leukocyte-released oxygen radicals and proteases.

Probably the earliest observation that neutrophils can occlude diabetic retinal capillaries is in Schroder *et al* 1991 whose observations in alloxan treated diabetic rats [57] showed local leukocyte accumulation was geographically associated with other vascular pathology such as endothelial cell damage, capillary nonperfusion, and extravascular leukocytes. Hatchell *et al*. [73] report the observation of white blood cells obstructing capillaries in retinas from diabetic cats. Both Miyamoto [75] and Ogura [76] observed acridine orange labelled leukocytes by scanning laser ophthalmoscopy in diabetic rat retina. There was significant elevation of leukocytes trapped in the retinal microcirculation in the early stages of diabetes compared to nondiabetic rats. Their hypothesis was that accumulation of leukocytes in diabetic retinas during the preretinopathy stage could cause microvascular occlusions and dysfunction, causing subsequent retinopathy and these occlusions occurred early. The leukocyte occlusions observed by these experimenters seemed random without any clustering though Joussen *et al*. [77] observed clustered endothelial cell damage even at an early stage. Leukocytes frequently get temporarily ‘held up’ at the entrance to a capillary in both normals and in diabetics because leukocytes are simply larger in diameter than most retinal capillaries (10 microns vs 6 microns). To enter the capillary requires an active cytoskeletal remodelling process to occur within the leukocyte. Also this means that flow in a capillary is temporarily blocked, but this is flow in a capillary with an intact endothelium and the capillary remains patent once the leukocyte passes through. This occurs over a generally short period of time, and in the non-diabetic, the leukocyte moves on without adhering to and damaging the endothelium by releasing ROS and enzymes or activating apoptosis. In the diabetic there can be adherence through complementary cell receptors on the activated leukocyte and the endothelium resulting in ongoing endothelial loss ultimately exceeding local endothelial replicative capacity causing an occluded, acellular capillary. All this supports leukocyte mediated capillary occlusion through leukocyte adhesion and endothelial cell damage with the initial step in the process of capillary occlusion being endothelial cell ICAM expression, dependent on VEGF [78]. So step 2, the change in concentration of this substance causes some change in the retinal capillaries resulting in an increased probability of capillary occlusion above essentially zero in the non-diabetic basal state has occurred.

#### Step 3: Capillary occlusion and resultant ischemia further increases VEGF production

Clearly capillary occlusion must produce ischemia. Oxygen is carried by patent capillaries to tissues. When the capillary becomes blocked and is no longer patent, the tissue receives no blood flow and this is the definition or at least the literal meaning of ischemia. However, there are a range of possibilities to consider. If there is dense packing of the capillaries in the network, occlusion of one could have minimal effect and all tissue could remain oxygenated. If the capillary network is extremely sparse, some cells are teetering on the edge of ischemia even before the occlusion and a large amount of tissue can become ischemic with the closure of a single capillary. Our model posits that blockage of a capillary produces tissue ischemia which upregulates a factor able to diffuse to adjacent capillaries and increase their likelihood of occlusion. The central nervous tissue is well known for having high metabolic requirements, e.g. 25% of the oxygen utilized at rest by a human is consumed by the CNS. However, the CNS, while it upregulates oxygen consumption with activity, as shown by fMRI, has functional changes that are quite small relative to tissue like muscle. In the CNS there is no requirement for a capillary network that has density adequate to cope with temporary, extremely elevated oxygen demands. The retina, because blood absorbs or scatters light impeding optimal ocular function, is a specialized part of the CNS which has reason to minimize capillary density. Therefore it is more likely in the retina that occlusion of a capillary produces tissue ischemia. The capillary networks used in our modelling are from actual subject imaging or from peripheral retinal capillary networks in the literature and were therefore not created for these modelling purposes. Also the oxygen diffusion coefficient was taken from the literature. Ischemia of the Mueller cell results in stabilization of HIF-1α which is then transported to the nucleus where it is able to act as a transcription factor for VEGF [79] which in turn further upregulates production of VEGF. Ischemia, or hypoxia, is known to induce endothelial cell production of ICAM-1 [80]. Under hypoxic conditions, HIF-1α, VEGF, and erythropoietin levels all increase rapidly in the inner retina, especially in the central region of the inner nuclear layer, the location of Mueller cell nuclei. If HIF-1α is disrupted in Mueller cells there is attenuation of the increased leakage and adhesion of leukocytes as well as decreased VEGF and ICAM [81]. Also, Mueller cells survive in the ischemic retina of diabetics. Step 3, that capillary occlusion results in local retinal ischemia and by some mechanism further elevates the level of the substance (VEGF) and also that ischemia does not act to kill the cells producing the substance, seems to be well supported.

#### Step 4: Elevated concentration of the substance in a local area increases the probability of occlusion of nearby capillaries resulting in the spatial propagation of capillary occlusion

The model posits that once a capillary is permanently occluded, the probability of a nearby capillary occluding increases. Once this irreversible capillary occlusion is initiated by an activated leukocyte adhesion, a local area of retina composed of Mueller cells and other retinal tissue has a drop in its oxygen tension. Within the Mueller cell HIF-1α is stabilized, migrates to the nucleus as a transcription factor, and further increases the production of VEGF [79]. This elevated level of VEGF diffuses to surrounding tissues, including capillary endothelium, and in those adjacent capillaries further increases local VEGF and ICAM-1 (step 2) with a resulting increase in the likelihood of occlusion. There is evidence supportive of this process. In spontaneously diabetic monkey retinas neutrophils are detected adjacent to areas with capillary closure [82]. This spatially selective concentration of adherent neutrophils means local endothelial adhesion must be elevated and though there is no specific immunohistological evidence of locally elevated ICAMs secondary to locally elevated VEGF, this is reasonable. Earlier work in human diabetics [83] found relatively large areas containing only cell-free capillaries and the margins of such fields were generally studded with microaneurysms, proliferated endothelial cells, and irregularities in the contours of venous walls Also in humans, increased numbers of adherent PMNs within retinal capillaries are observed adjacent to sites of capillary non-perfusion or degeneration [84]. All this is consistent with locally elevated VEGF generated in the ischemic retinal areas, diffusing to affect surrounding retinal capillaries in both an ischemic way, by increasing leukocyte adhesion, and in an angiogenic way, by causing local vasoproliferative type changes.

The most relevant animal model is that of spontaneous diabetes in a primate. The earliest histologically documented changes observed were dot/blot hemorrhages, cotton-wool spots (cotton-wool spots are non-perfused nerve fiber layer areas), and small non-perfused retinal areas [85]. Microaneurysms, often associated with small intraretinal microvascular abnormalities (IRMAs), were located adjacent to areas of nonperfusion, shown by lack of ADPase positive blood vessels. Large areas of capillary loss always involved arteriolar pruning. These observations are consistent with the hypothesis that the driver of ischemic retinopathy is the development of small ischemic areas which then propagate locally. The arteriolar pruning would occur as vascular branches both decrease their flow due to loss of capillaries, and are then subjected to higher VEGF levels. The microaneurysms and IRMA represent canonical angiogenic consequences of VEGF, elevated at the edge of the ischemic areas. Over the disease course, large ischemic areas occur, meaning that generally the microaneurysms and IRMA occur later in the disease process. Within this framework a large retinal vessel, by creating a surrounding oxygenated zone, may act as a barrier to propagation of capillary loss. This can be seen both in clinical angiograms and in histology (see Figure 2D in [85]). Similarly, in induced or spontaneous diabetes in monkeys, early background retinopathy was characterized by capillary dropout and IRMAs [86, 87]. As is commonly experienced in humans, no clinical sign of diabetic retinopathy was detected in monkeys with spontaneous or STZ-induce diabetes for 4 to 13 years provided the monkeys were not also hypertensive. Though there is no existing data on local VEGF and ICAM levels as a function of distance from an area of retinal ischemia, fairly strong observation support is present in the literature that is consistent with step 4, the spatial propagation of capillary occlusion.

There are a several types of in vivo experiments which either examine the results of injection of VEGF into animal eyes or the results of variation in the level of VEGF in animal models. VEGF is considered to be a proinflammatory molecule whose vitreal levels are highly correlated with retinal neovascularization and edema [55, 88, 89]. In mice even a temporary VEGF expression in photoreceptors, without elevated glucose, demonstrated retinal vascular changes similar to diabetic retinopathy, including retinal leukostasis, capillary endothelial cell and pericyte loss, and acellular capillaries [90]. There are also animal diabetic models in which an intervention, ranging from oral or intravitreal pharmaceuticals or genetic manipulation are able to prevent the development of diabetic retinal vascular changes [50, 77, 91–94]. To the best of our knowledge, though acting by quite different pathways, and some without altering VEGF, all prevent the adhesion of leukocytes to the retinal endothelium thereby preventing development of retinal capillary occlusion and attenuating signs of diabetic retinopathy. Thus, the occlusion process seems essential to diabetic retinopathy and this model will behave in the same way since the elevation of capillary occlusion probability is essential to development and propagation of ischemia. A number of interesting results were obtained by Wang *et al*. 2015 [95] using a conditional Mueller cell VEGF knock out model (CVKO). They examined the levels of proinflammatory markers in CVKO mice by IB analysis for intercellular adhesion molecule-1 (ICAM1) and tumor necrosis factor-α (TNFα), 2 months after STZ injection. Compared with controls, the CVKO mice showed 62.3% and 52.9% reduction of ICAM1 and TNFα (Table 1 in [95]), respectively and showed a 75.0% reduction of adherent leukocytes, a cardinal feature of retinal inflammation in DR.

Direct exposure of the retina to VEGF at levels comparable to those found in patients with diabetic neovascularization also supports this hypothesis. Just 9 days post uni-ocular VEGF injection in monkeys, the intraluminal volume of capillaries in the deep retinal plexus was decreased by 5+ fold due to capillary endothelial cell hypertrophy as measured by both EM and light microscopy [96]. This suggests a preferential occlusion of these capillaries of the inner nuclear layer which are adjacent to Mueller cell bodies. An acute exposure to VEGF results in endothelial cell hypertrophy sufficient to prevent flow in these capillaries which were only about 6 microns in lumen diameter prior to the 5 fold swelling. It was not shown in the study but capillary occlusion and retinal ischemia would be expected. A classic study using VEGF injections over a longer period of time was Tolentino 1996 [97]. In this study animals received from 1 to 26 injections of VEGF. Even a single injection yielded large vessel dilation, tortuosity, and vascular leakage, all canonical changes seen with elevated VEGF. After 6 injections (one every 3 days) in one animal, venous beading is visible and areas of nonperfusion were present in the midperiphery. In another animal after 4 injections, large areas of capillary closure appeared. Neovascularization of the disk appears much later (80 days) and associated with “extensive areas of avascular retina temporally”. The authors of this paper anticipate the adverse positive feedback hypothesis of this paper (without any geographic dependence or modelling) in this quote “These data show that VEGF alone can trigger retinal ischemia through capillary closure in normotensive eyes. This activity could initiate a positive feedback loop, further increasing VEGF levels.” In summary, the molecule VEGF is a reasonable candidate for a one molecule model of the progression of the diabetic capillary occlusive process even if its actions are often through other molecules, e.g. ICAMs, or complex processes, e.g. leukostasis and capillary occlusion

## Methods

### Human subjects

The protocol for AOSLO imaging used to provide retinal vascular images used in this study was approved by the Indiana University Institutional Review Board and adheres to the tenets set forth in the Declaration of Helsinki and the Health Insurance Portability and Accountability Act regulations. Written informed consent was obtained from all subjects.

### Model considerations

**Fig 2:**
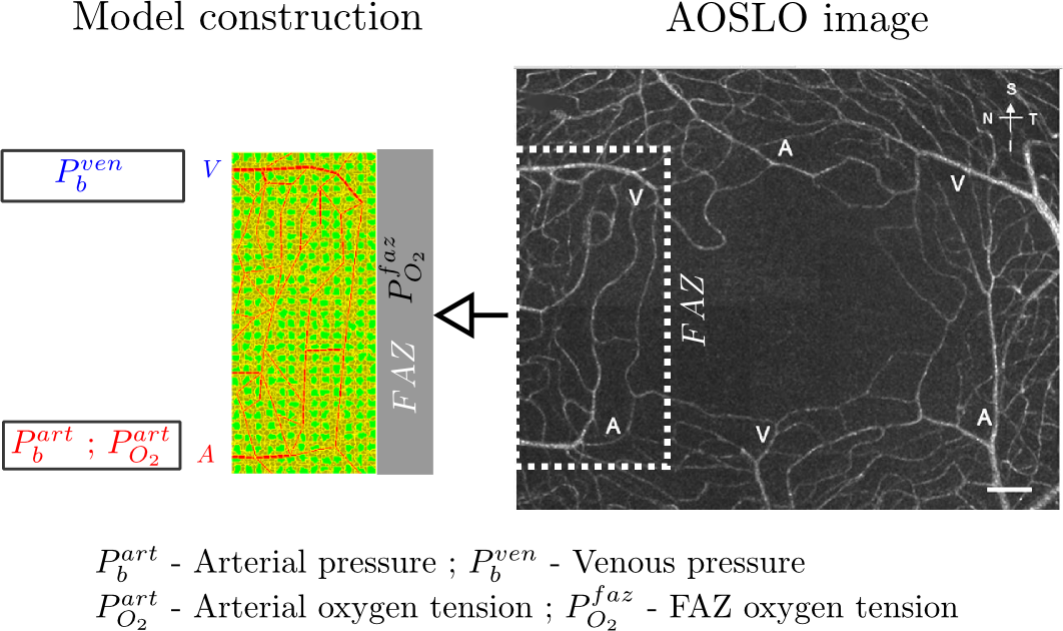
Model abstraction and construction of human perifoveal capillary network from an Adaptive Optics Scanning Laser Ophthalmology (AOSLO) image. On the right, AOSLO image shows juxtafoveal capillaries adjacent to the foveal avascular zone (FAZ). This is a normal capillary map in a patient without diabetes. On the left, a model schematic shows the reconstruction of the capillary network framed in the AOSLO image with cells filling in empty space between vessels uniformly. Capillary network has arteriole (A) and venule (V) termini marked. Boundary blood pressures are assigned for A and V termini. Boundary oxygen tensions are assigned for A terminus and for FAZ whereas venous oxygen tension is model dependent. Model objects in red, green, brown are capillary blocks (**CAP**), Mueller cells (**MC**) and other retinal cells (**OT**) respectively. Yellow pixels surrounding objects are object borders, which are muted in other figures of the manuscript. Note that this example shows a macular capillary network from a different subject than that shown in CASE 1. Diameter of the FAZ in this ASOLO image is approximately 500 microns. The scale bar is 100 microns.

To capture the events of progressive capillary occlusion we implemented a quantitative model of the anatomical features mentioned above in Compucell3D [98]. As shown in Fig 2, beginning with an AOSLO scan of the perivascular fovea, a full vascular model from an arteriole to a venule with the linking capillaries is included with oxygen advection, oxygen diffusion, and oxygen consumption. This paper develops a conceptually simple model of the diabetic retina treating Mueller cells as the sole retinal source of VEGF and assumes a slight elevation of VEGF production by Mueller cells in a diabetic retina higher than that in the normal retina [99]. Physiologically VEGF is a necessary neurotrophic factor in the retina and is normally present at low levels [100–102]. In the model VEGF is produced by Mueller cells locally in variable amounts based on oxygen saturation. VEGF diffuses from the Mueller cells and is consumed by cells including endothelial cells but is not transported away by advection. The model vessels have endothelial cells which respond to local VEGF levels by an increased probability of occlusion with elevation of local VEGF and also by leaking if local VEGF exceeds a threshold level. The model is cycled many times and if a capillary occlusion occurs, all flow rates, steady state oxygen tension and VEGF levels are recalculated. The model’s treatment of occlusion is an irreversible decrease of capillary diameter to zero. An important assumption is that the vascular supply to each area of retina is critical in that occlusion of a capillary will result in ischemia of an area of physiologically dependent retina with a resultant elevation of VEGF synthesis by the locally ischemic Mueller cells. We do not know that this has been proven but the constraints imposed by evolution on the visual apparatus make this assumption reasonable. To quote Chan *et al*. 2012 [102] “It is likely that retinal capillary networks are morphometrically adapted in order that the balance between cellular nutrition and optical clarity can be achieved.” Note, however, that with the variations of capillary spacing seen anatomically, all areas of retina would not have equal dependence on a single supplying capillary. In a network based on actual capillary anatomy different areas of retina could be more or less critical as a result of variation in local capillary density. There would thus be greater or lesser propagation of capillary closure by the adverse feedback mechanism. Small capillary diameter adjustments can also occur, e.g. slightly increasing diameter with increased flow after each capillary occlusion. Maps at various model times are made of capillary network structure, flow, oxygen tensions, VEGF, and retinal edema. These are the output measures as well as summary graphs of the system such as total flow and average distance from an intact capillary.

Model capillary occlusions are always probabilistic based on local VEGF levels and the calculated flows of the capillary segments. Capillary networks of several types were utilized including physiologically unlikely hexagonal capillary network with introduced deletions, physiologically realistic peripheral retinal ‘ladder’ capillaries [103] and an actual perifoveal arteriovenous sector capillary map obtained from adaptive optics scanning laser ophthalmoscopy (AOSLO) imaging of a subject. The hexagonal map was used to explore the dependence of capillary occlusion progression on amount of tissue dependent on capillaries by varying the scale of the hexagons. Both the macula and the peripheral retina are clinically important, with the macular area being the location of ischemia as well as macular edema affecting visual acuity, and the periphery being the major source of the ischemia and resultant VEGF production which results in retinal neovascularization. Though it is not clear if it is physiologically appropriate to utilize the same fundamental model parameters for the macula and periphery given the distinct peripheral retinal and vascular architecture [103, 104] we altered only the capillary diameters and network structures. In the main text we address primarily a sector of the perifoveal capillary network from AOSLO imaging (CASE 1) and filled the open space between vessel segments with cells of anatomically reasonable sizes (S4 Table). Capillary diameters were estimated based on the AOSLO image. Model inputs such as terminal hydrostatic pressure and arteriolar blood oxygen tension were estimated from published results (S4 Table, S15 Fig). Vascular flows, oxygen and VEGF fluxes were calculated and resulting tissue oxygen tension and VEGF levels were determined in the model. A large number (362) of replicate runs were made of the subject’s capillary network in order to assess the vulnerability of distinct capillaries given the probabilistic nature of the model of individual capillary occlusion. S1 Text details mathematical descriptions, parameter selection and influence and boundary and initial conditions of the model. S2 Text briefly treats a run of the model in the macular area with a different initial capillary occlusion (CASE 2). S3 Text treats a hexagonal network with some deleted edges, and S4 Text treats a peripheral schematic ladder capillary network modelled on that of the peripheral human retina. The peripheral ladder capillary network seen in the peripheral retina has significant implications about the development of the peripheral ischemic wedge-like sectors seen clinically between arterioles and venules whereas the hexagonal model was informative about the dependence of capillary occlusion propagation on distance between capillaries.

Many models have been constructed to study problems at the interface of vasculature in various tissues: skeletal muscle [105, 106], brain [107, 108], vascular tumor [109, 110] and retina [111]. Shirinifard *et al*. employed a 3D multi-cell model to successfully recapitulate the three patterns of progression of age-related macular degeneration and suggested that defects in adhesion were the dominant contributor to initiation and development of choroidal neovascularization [111]. Cringle *et al*. divided retina into multiple layers and used a mathematical model to calculate the oxygen tension in each layer in terms of oxygen consumption rate in that layer and the oxygen level in choroidal capillaries [112, 113]. McDougall *et al*. studied angiogenesis during normal retinal development using a hybrid discrete-continuum mathematical model and computationally simulated the structure of a retinal vascular plexus that agreed with the whole-mount retinal vasculatures at different stages of development [114]. Our model deals with a different pathophysiological issue: progression of ischemia and edema in diabetic retinopathy based on a local VEGF-dependent mechanism of propagation of capillary occlusions. Unlike this study, Gandica *et al*. [115] developed a computational model of retinal ischemia studying the effect of critical sizes and densities of localized blockages of retinal vasculature on the emergence of diabetic retinopathy. In their model, various sizes of local blockages of vessels, assumedly caused by destabilizing proteins such as Angiopoietin-2, were randomly distributed in the region of interest and areas of derived hypoxia were examined regarded as an indicator of potential phenotypes of diabetic retinopathy. An important conclusion in their study is that local blockages with smaller size than characteristic irrigation length, if their densities exceed a critical threshold, likely result in large hypoxic areas because of a cooperating effect. A limitation of the model, as the authors also noted, is the simplified consideration of oxygen transport.

To ensure that we respect the specific geometry of the retinal capillary network, we use networks from actual subject imaging or from peripheral retinal capillary networks in the literature and were therefore not created for these modelling purposes. Also the oxygen diffusion coefficient was taken from the literature.

### Model description

**Fig 3:**
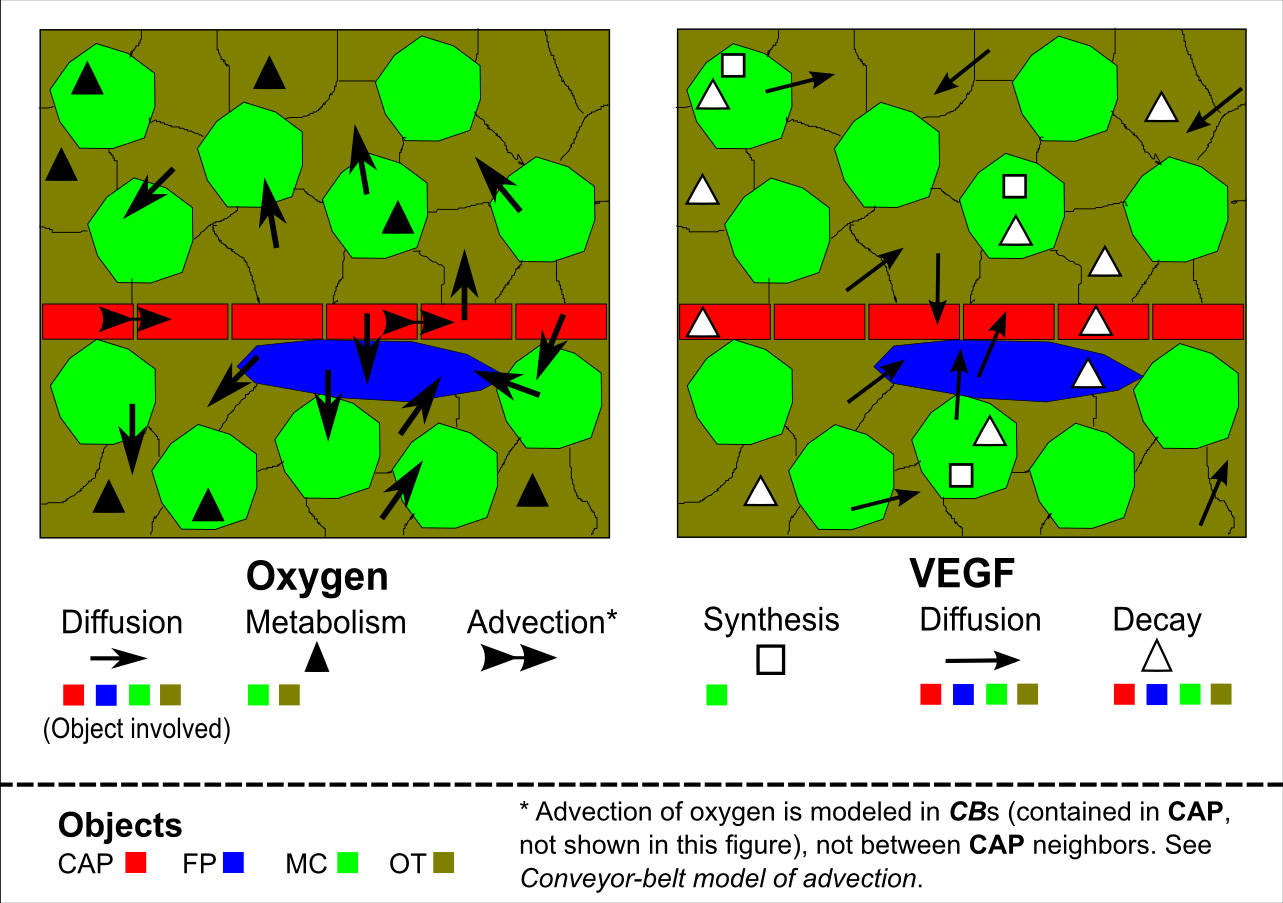
Schematic of oxygen and VEGF fluxes. Colored blocks represent model objects: Capillary block (CAP in red), Fluid portion (FP in cyan), Mueller cell (MC in green) and Other retinal cells (OT in brown). Markers in the form arrows, triangles and squares are used to represent modeled fluxes. On the left are the oxygen fluxes including advection, diffusion and metabolism. Oxygen advection is modeled for object ***CB***. Oxygen diffusion is modeled for object pairs among **CAP**, **MC**, **OT** and **FP**. Oxygen metabolism is modeled only for objects **MC** and **OT**. On the right are the VEGF fluxes including synthesis, diffusion and decay. VEGF synthesis is modeled for object **MC** again as the model’s only VEGF source. VEGF diffusion is modeled for object pairs among **CAP**, **MC**, **OT** and **FP**. VEGF decay is modeled for objects **CAP**, **MC**, **OT** and **FP**. The arrangement of model objects do not necessarily reflect detailed configurations constructed in the simulations.

In this study, we explore the effect of focal capillary occlusion on decreasing local oxygenation of retinal cells, resultant elevation in VEGF, and the consequences in terms of propagation of capillary occlusions and formation of edema using a computational model. The anatomy of this capillary network was determined from a normal patient using AOSLO (Fig 2). This network is an arteriovenous sector with the capillaries connecting the arteriole and venule, the foveal avascular zone on one edge and retinal tissue on the other borders. We computationally reconstruct the network and initialize the simulation with boundary blood pressures and arterial and FAZ oxygen tension (Fig 2). The major entering arterial node and the exiting venous node are assigned with blood pressures $P_{b}^{art}$ and $P_{b}^{ven}$ respectively. These pressures would not be expected to depend on the diabetic state and remain fixed. Other boundary nodes are assigned with blood pressures of intermediate values (S15 Fig, also see **Boundary conditions and initial state of simulation** in S1 Text for details). The FAZ region is treated as an oxygen source, supposedly supplied by choroidal capillaries. The entering arterial node and the FAZ region are assigned with oxygen tension $P_{O_{2}}^{art}$ and $P_{O_{2}}^{faz}$ though the exiting venous oxygen tension is model dependent. Other boundary nodes with incoming blood flow are assigned with oxygen tensions of smaller values (S15 Fig). Hypoxia-induced elevated secretion of VEGF in Mueller cells is known as an occurrence in diabetic retinopathy [115–117]. FAZ region is treated as a sink for VEGF. We modelled the oxygenation and local VEGF levels within this retinal sector following a focal capillary obstruction. Within the sector of capillary network modeled, the arterial side receives oxygen-rich blood, while from the venous terminus carries away blood with lower oxygen tension. Oxygen diffuses into tissue space from capillaries, where it is consumed and metabolized by retinal cells. Hypoxia of local retinal tissue is induced by a local capillary segment occlusion, and production of local VEGF, dependent on the level of hypoxia is upregulated in ischemic Mueller cells. VEGF released by Mueller cells diffuses to other nearby patent capillary segments, which probabilistically derive more occlusions based on an underlying and not visibly modelled upregulation of ICAM and increased probability of leukostasis. The schematic of oxygen and VEGF fluxes is shown in Fig 3.

### Model objects and processes

The described computational model consists of four generalized model cell types: capillary block (**CAP**), fluid portion (**FP**), Mueller cell (**MC**) and other retinal cell (**OT**). Along with these four generalized cell types in the model is another model object called the conveyor-belt block, ***CB***, which is an object associated with the capillary block and introduced for modeling of oxygen advection. In addition to five model objects, two chemical fields exist in the model: oxygen and VEGF. Modeled processes include advection of blood carrying oxygen, diffusion and metabolism of oxygen, and synthesis, diffusion and decay of VEGF. As detailed in S1 Text and summarized in S1 Table, model objects have the following properties and are representative of various retinal cells:

1. **CAP** is the structural element of the capillary segment. **CAP** functionally represents a capillary segment with endothelium and blood within the capillary lumen. **CAP** is involved in modules of oxygen diffusion and VEGF diffusion and decay. **CAP** has three states: “normal”, “leaky” and “occluded”. “Normal” **CAP** can transition to “leaky” state if local VEGF level is greater than a threshold value. Both “normal” and “leaky” **CAPs** can transition to an “occluded” state if the occlusion condition, whose probability is raised by an elevated VEGF level and lowered by elevated blood flow velocity, is met (S2 Table). Once one **CAP** becomes “occluded”, the flow of that capillary segment from node to node is set to zero. Also, “Occluded” is a permanent or irreversible state for the **CAP**. (More details on capillary occlusion in S1 Text)
2. **FP** is the structural element of and represents incremental volume of retinal edema, cystic areas of fluid within the retina. **FP** represents fluid leaked from a **CAP** while it is in the leaky state. **FP** is modeled as a “cell”-like object that is well trapped by surrounding objects. **FP** is involved in modules of oxygen diffusion and VEGF diffusion and decay. (More details on edema formation in S1 Text)
3. **MC** is involved in modules of oxygen diffusion and metabolism and VEGF synthesis, diffusion and decay. The **MC** has two states: “normal” and “hypoxic”. The “Normal” **MC** can reversibly transition to a “hypoxic” state synthesizing VEGF only when cellular oxygen tension is below a threshold value. In the model only the “hypoxic” **MC** has the capability of synthesizing VEGF though in the diabetic retina a number of other cells have at least some ability to synthesize VEGF.
4. **OT** broadly includes all other retinal cells other than Mueller cells, i.e. the neural retina including astrocytes and microglia but excluding photoreceptors supplied with oxygen from the choriocapillaris. **OT** is involved in modules of oxygen diffusion and metabolism and VEGF diffusion and decay.
5. ***CB*** is a functional element created in the model to “convey” oxygen along a linear pipe, thus ***CB*** or conveyor belt is not an anatomical element corresponding to a retinal structure but is a model device allowing mathematically convenient modelling of oxygen advection. ***CB*** is involved in the module of oxygen advection. Each ***CB*** is associated with a “host” **CAP**, which usually contains more than one ***CB***. In contrast to the fixed pre-defined size of **CAP** on every capillary segment, size of ***CB*** is proportional to the blood flow velocity on its host capillary segment.

**Fig 4:**
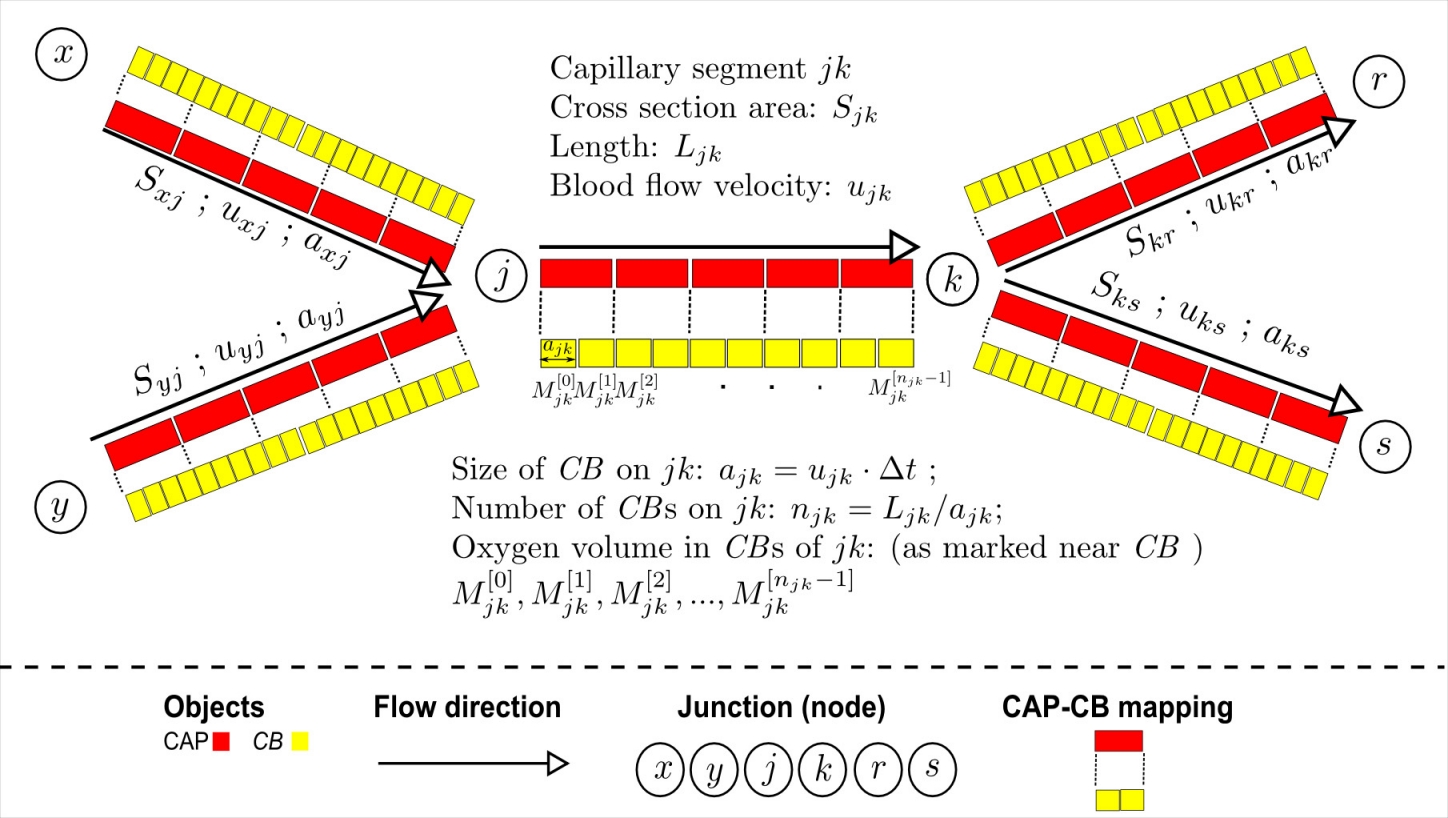
Schematic of conveyor-belt model of oxygen advection. The schematic shows a small capillary network with five segments and two junctions. Along the blood flow direction, marked as arrow with empty triangular head, the first junction has merging blood flow and the second branching blood flow. Blocks in red are **CAP**s, building blocks, or structural elements (visually present in model configuration), of capillary segments. Blocks in yellow are ***CB*s**, functional elements for advection. Size of a **CAP** is fixed throughout a simulation, so each capillary segment has fixed number of **CAP**s. In contrast, the size of a ***CB*** is always proportional to flow velocity on the capillary segment, so flow velocity and accordingly the number of ***CB***s on a patent capillary segment can vary following an occlusion elsewhere as flows in the network are adjusted to the changed network resistance structure. During each time step of advection, a ***CB*** passes its oxygen volume to the next ***CB***. Importantly, the size of a ***CB*** is equal to flow velocity of host capillary segment multiplied by time step of advection, which means the “conveying” speed of oxygen from one **CB** to next is exactly equivalent to the flow velocity in that capillary segment. At a merging junction, the upstream capillary segments add the oxygen volumes in their *last* **CB**s and pass the total to the *first* **CB** of the down stream capillary segment. At a branching junction, the parent capillary segment distributes the oxygen volume in its *last* **CB** into the *first* **CB**s of the daughter segments according to conservation of blood flow volume. Mathematical descriptions were detailed as equations (9)-(11) in S1 Text. Each ***CB*** is associated with, or mapped to, a host **CAP**. The modules of advection (involving ***CB***s) and diffusion (involving **CAP**s) are connected with processes that convert oxygen volumes between the host **CAP** and its associated ***CB***s. (see Fig 5).

**Fig 5:**
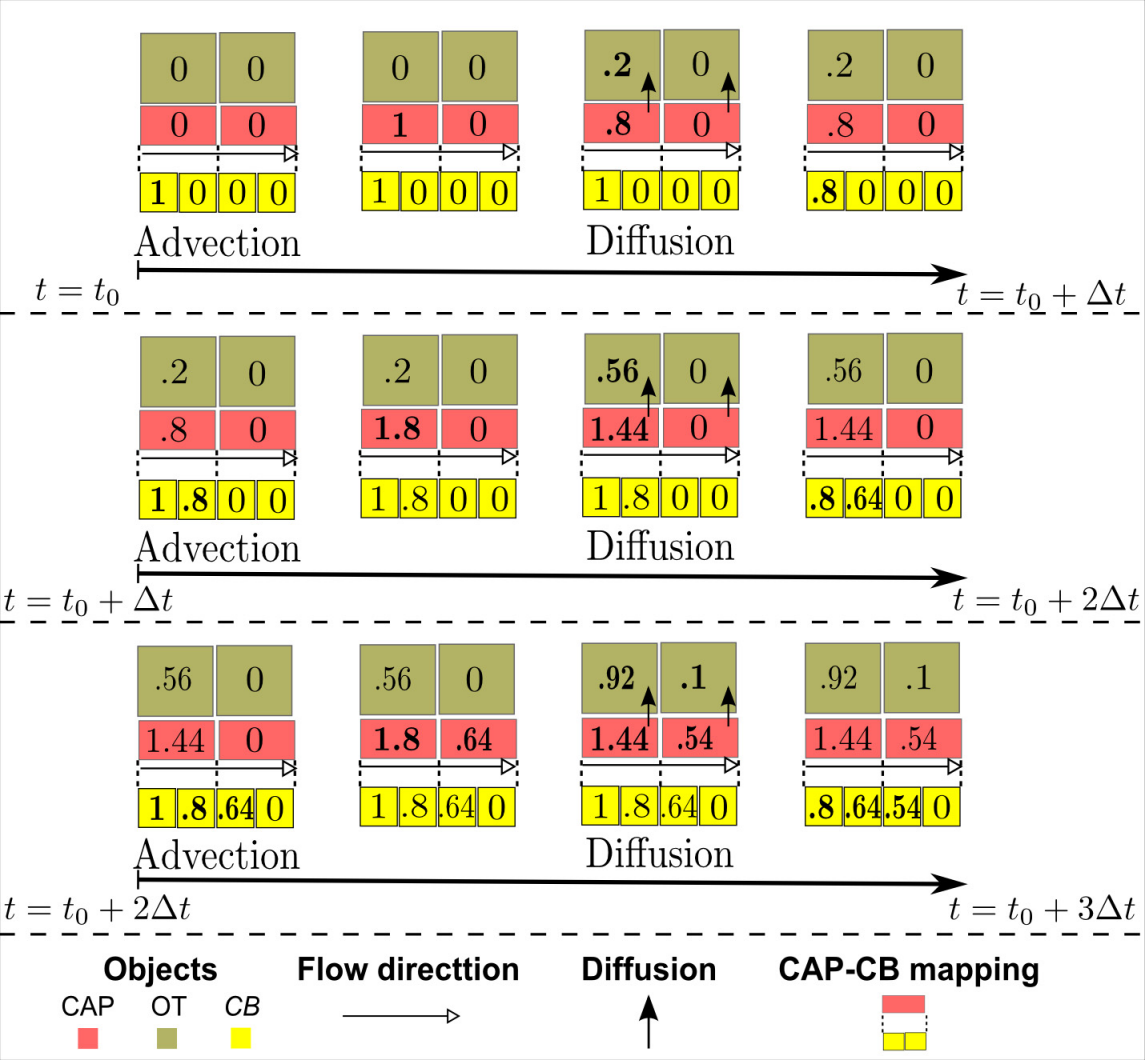
Procedure of sequential simulation of advection and diffusion illustrated with an example. During each time step, simulation of advection precedes simulation of other processes including diffusion. After advection (involving the ***CB***s delivering the blood containing oxygen) and before diffusion (involving **CAP**s), a **CAP** always sums up oxygen volumes in all its associated ***CB***s and update its *pre-diffusion* oxygen volume. Simulation of diffusion updates the **CAP** to have its *post-diffusion* oxygen volume. After diffusion and before advection at the next time step, the associated ***CB***s must have the same *relative* change of oxygen volumes as the **CAP** gains or loses during diffusion, namely, percent change from *pre-diffusion* to *post-diffusion* values. This mathematically recognizes the conservation of oxygen. Three consecutive time steps are shown in the example. A naïve assumption here for this illustration is that the first **CB** always receives 1 unit oxygen volume from upstream (not drawn). Another simplification made for this illustration is that only diffusion between the **CAP** (shown in red) and nearby **OT** (shown in light brown) are considered. In the actual model **OT** to **OT** diffusion is also treated. Note that in a certain step, boldface numbers represent values being changed or updated. During the first time step from *t*_0_ to *t*_0_ + Δ*t*, while the second **CAP** and its associated ***CB***s still have a zero oxygen volume, the first **CAP** and associated ***CB***s undergo (1) the process of *advection* that passes **1** unit oxygen volume to first ***CB***, while **CAP**s are not involved; (2) an intermediate step that updates **CAP**’s pre-diffusion oxygen volume by adding **1** (its first associated ***CB***) and **0** (its second associated ***CB***); (3) the process of *diffusion* delivers **0.2** to **OT** in contact (amount assumed for convenience in this example, and again **OT**-**OT** diffusion is ignored in this example) and **CAP**’s post-diffusion oxygen volume becomes **0.8**, while ***CB***s are not involved; (4) a last step in the time period that updates **CAP**’s associated ***CB***s’ oxygen volumes by subtracting 0.2/1 = 20% (diffused/pre-diffusion), the first CB thus having **0.8** oxygen volume now. During the second time step from *t*_0_ + Δ*t* to *t*_0_ + 2Δ*t*, similar verbal “simulation” goes. (1) process of *advection* goes as another **1** oxygen volume is passed to first ***CB*** and **0.8**, previously held by the first ***CB***, is passed to the second ***CB***; (2) an intermediate step adds **1** and **0.8** to first **CAP**, still none added to the second **CAP**; (3) the process of *diffusion* updates first **CAP**’s oxygen volume to **1.44**, with **0.36** diffused out; (4) last step updates oxygen volumes in both of the host **CAP**’s two ***CB***s, again by subtracting diffused fraction 0.36/1.8 = 20%. During the third time step from *t*_0_ + 2Δ*t* to *t*_0_ + 3Δ*t*, *advection* now passes oxygen volume **0.64**, previously held by the second ***CB***, into the third ***CB***, which is associated with the second **CAP**. An intermediate step updates both **CAP**s by summing up oxygen volumes in their associated ***CB***s. Process of *diffusion* now changes the oxygen volumes of both **CAP**s, with the first and second diffusing out 0.36/1.8 = 20% and 0.1/0.64 = 15.625% respectively. The last step subtracts the oxygen volumes of their asscociated ***CB***s’ with the percent change.

To model oxygen advection, we discretize each capillary segment into a one-dimensional sequence of equally-sized ***CB***s and simulate oxygen advection using a “conveying” action, which moves a volume of oxygen in a given ***CB*** to its next downstream connected ***CB*** (Fig 4). The size of a ***CB*** is proportional to both flow velocity and the time step for advection. In the case of merging at a junction, total oxygen volumes in last ***CB***s of the predecessor capillary segment are conveyed to the first ***CB*** of the successor capillary segment (Fig 4). In the case of branching at a junction, conservation of blood flow is enforced to appropriately distribute the oxygen volume in the last ***CB*** of the parent capillary segment to the first ***CB*** of the daughter capillary segments (Fig 4). Mathematical descriptions were detailed as equations (9)-(11) in S1 Text. The benefit of the conveyor-belt model of oxygen advection is the flexibility of adjusting the number of ***CB***s in a **CAP** without interference with oxygen diffusion. When occlusion occurs, flow velocities change on the capillary network and accordingly the size and number of ***CB*** on a **CAP** also change. This alters the discretization for oxygen advection, but the module of oxygen diffusion remains unaffected. A simple rule is followed to associate a ***CB*** with **CAP**: a ***CB*** is associated with a **CAP** if the ***CB***’s center is enclosed by the **CAP**’s volume. Then, the following sequence of processes is used to link modules of advection and diffusion: (1) oxygen advection in ***CB***s on each capillary segment at time step *t*_0_; (2) conversion of oxygen volume from ***CB***-level to **CAP**-level just before diffusion at time step *t*_0_; (3) oxygen diffusion involving **CAP**s and their surrounding objects at time step *t*_0_; (4) conversion of oxygen volume from **CAP**-level to ***CB***-level just before advection at time step (*t*_0_ + Δ*t*_*f*_) (Fig 5). (More details on the CB model of oxygen advection are in S1 Text).

To model diffusion of a chemical field, we assume that each generalized cell has a uniform intra-cellular chemical concentration and that diffusion occurs at the interface between neighboring cell pairs. Metabolism of oxygen and synthesis and decay of VEGF are modeled as an intra-cellular process (Fig 3). As for diffusion, the rate of exchange of oxygen or VEGF between generalized cells is proportional to inter-cellular gradient of concentration and inter-cellular contact surface area. Metabolism of oxygen obeys Michaelis-Menton kinetics, which assumes variability of oxygen consumption given different intracellular oxygen tension. Synthesis of VEGF in hypoxic **MC** is dependent on intra-cellular oxygen tension and VEGF level. Decay of VEGF is modeled using first-order kinetics. FAZ region is treated as both an oxygen source and a VEGF sink. (Details on oxygen and VEGF fluxes in S1 Text)

### Model workflow

**Fig 6:**
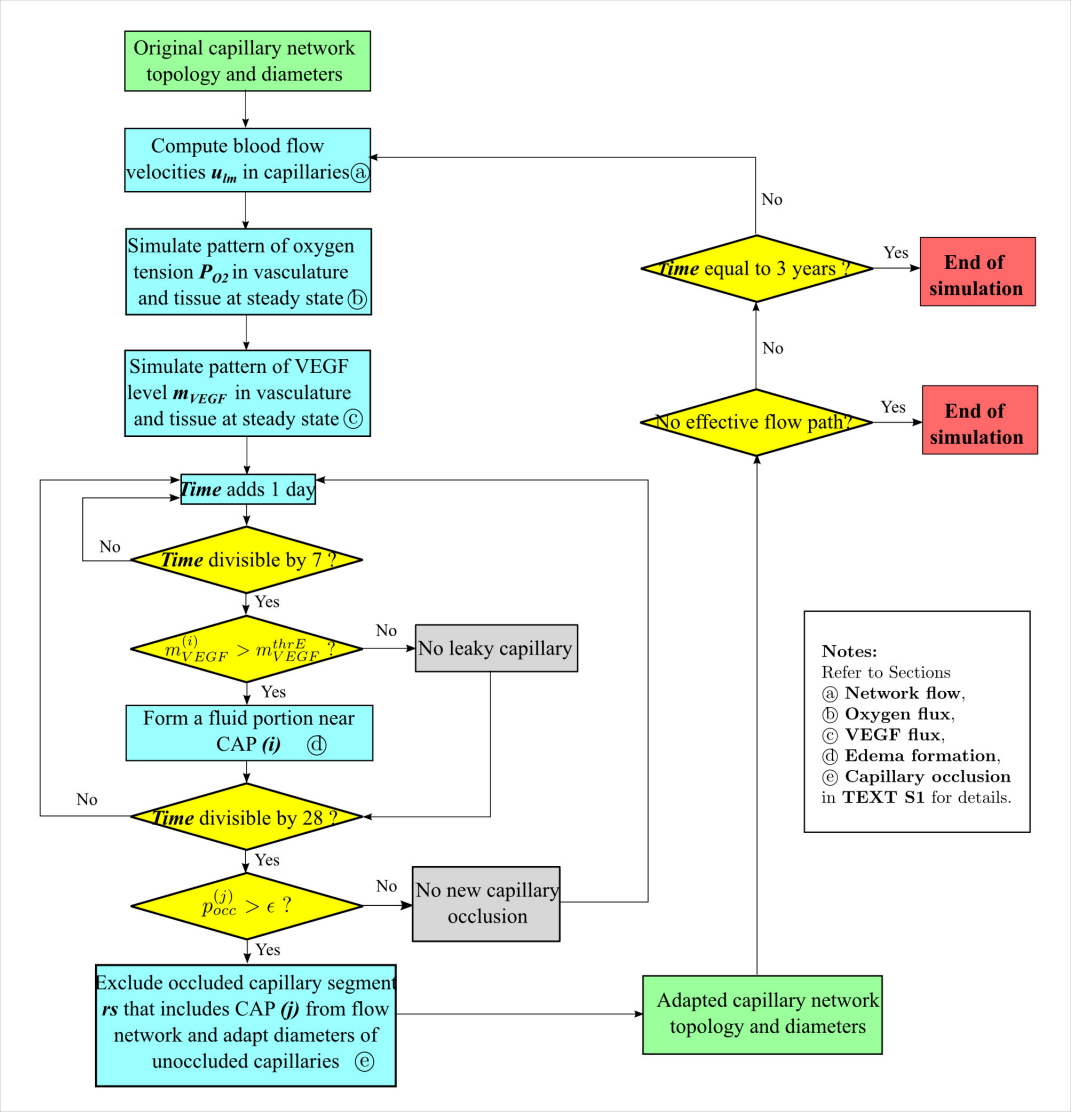
Model workflow. Simulation is initialized with input of the original capillary network topology and structure as well as the component cells. Computation of flow velocities and simulation of oxygen and VEGF fluxes occurs next. Each model week the model addresses capillary leakiness and each model month capillary occlusion is evaluated. (1) If no new occlusion occurs, the model simply repeats checking for edema formation and capillary occlusion until occlusion occurs or until a pre-assigned simulated time (3 years at our temporal conversion rate) is arrived at. (2) Once a new occlusion occurs, the network topology is changed and its structure is adapted to eliminate flow in the irreversibly occluded capillary, followed by a new iteration of previous steps. The process stops if either no more effective flow paths exist, invalidating flow velocity calculations, or the pre-assigned simulated time is arrived at.

Our simulation involves three distinct intervals of *time*: the time step of integration for oxygen and VEGF *flux*, Δ*t*_*f*_; the time interval to check *edema* formation, Δ*t*_*e*_; and the time interval to check for *occlusion*, Δ*t*_*o*_. The Δ*t*_*f*_ is chosen so that the differential equations of fluxes are properly integrated. In contrast, Δ*t*_*e*_ and Δ*t*_*o*_ are selected so that possible edema formation and capillary occlusion take place at a significantly *slower* pace (months to years), as compared with *fast* establishment (seconds) of the oxygen and VEGF steady state following a newly derived occlusion. With Δ*t*_*f*_ = 0.002*s*, Δ*t*_*e*_ = 7 *days*, Δ*t*_*o*_ = 28 *days*, a model workflow is shown in Fig 6. The time for oxygen and VEGF steady state is basically mandated by physics whereas the other longer times for edema and capillary occlusion are chosen to allow the model to progress in rough accordance to the progression of clinical disease.

1. **Input of network anatomy.** The simulation starts with input of a capillary network and cells. Both structural and topological information of the capillary network is required. In our model, initial structural and topological information is determined by digitization of an experimental image obtained by AOSLO imaging. Diameters of capillaries (*d*^*cap*^ were estimated to follow a Gaussian distribution with mean value of 5 μm and standard deviation of 0.5 μm based on measurement of the AOSLO image, and the terminal venule and arteriole were measured to have larger diameters up to 10 μm. This structural information, with topological information of nodes and edges, is read into Compucell3D to reconstruct the capillary network. Next, the Mueller cells, **MC**s, were organized uniformly into the open space surrounding the capillary network. These are of roughly anatomical size. The space not occupied by **MC**s becomes the other tissue (**OT**). (More details in **boundary conditions and initial state of simulation** of S1 Text)
2. **Computation of flow velocities.** Blood flow directions and rates were determined using the Poiseuille equation, with hydrostatic pressures at boundary nodes given and fixed through the simulation. Boundary nodes refer to all those extending to the capillary network outside the region of interest, including the arterial inlet and venous outlet. (More details in **Network flow** of S1 Text).
3. **Simulation of oxygen fluxes.** Simulation of oxygen advection, diffusion and consumption are divided into three separate sub-modules sharing the same time step of integration Δ*t*_*f*_. An important feature of the model is that advection is simulated at ***CB*** level, while diffusion and consumption are simulated at inter-cellular and cellular levels respectively. Therefore, before actual simulations of advection, a process is needed which reads current **CAP**-level oxygen volume to update ***CB***-level oxygen volume. After simulation of advection by the ***CB***s and before simulation of diffusion and consumption of oxygen, a process is needed to convert ***CB***-level oxygen volume to **CAP**-level oxygen volume. The simulation of oxygen fluxes is executed until all model objects arrive at a steady state of oxygen tension. (More details in **Oxygen flux** of S1 Text)
4. **Simulations of VEGF fluxes.** Simulations of VEGF synthesis, diffusion and decay are divided into separate sub-modules which share the same time of integration as the oxygen flux, Δ*t*_*f*_. This module follows immediately after the simulation of oxygen fluxes. The simulation of VEGF fluxes is executed until all model objects arrive at the steady state of VEGF level. (More details in **VEGF flux** of S1 Text).
5. **Formation of edema.** Every period of Δ*t*_*o*_, the condition for edema formation is checked for all **CAP** objects. Once local VEGF level at a **CAP** exceeds a pre-defined threshold $m_{VEGF}^{thrE}$, edema is formed near the **CAP**. The edema is modeled as a pseudo cell **FP,** which is created every Δ*t*_*o*_ as long as the requirement of supra-threshold VEGF is met, namely, the **CAP** remains in a “leaky” state. Fluid portions of the pseudo cell get eliminated at the bottom of the system, which represents the function of the retinal pigment epithelium removing excess fluid (More details in **Edema formation** of S1 Text).
6. **Probabilistic dependence of capillary occlusion.** Every period of Δ*t*_*e*_, the probability function for determining capillary occlusion is checked for all **CAP** objects. If the requirement, which is dependent on the intracellular VEGF level of a **CAP** and blood flow velocity of the capillary segment, is met, the **CAP** turns to the “occluded” state, and so do all **CAP**s that belong to the same capillary segment. Consequently, the capillary segment acquires an infinite flow resistance (in the model, an infinitesimal number is now assigned to the diameter of the segment) and is effectively occluded. (More details in **Capillary occlusion** of S1 Text) This is the model’s representation of the process of capillary occlusion by a leukocyte. A high flow is assumed to make adhesion of a leukocyte less likely as mechanically the leukocyte is experiencing more pressure pushing it down the capillary. The dependence on level of VEGF subsumes the dependence of local ICAM levels on local VEGF with higher VEGF levels giving higher ICAM levels and increased leukocyte adhesion.
7. **On occurrence or absence of a new capillary occlusion.** If occlusion occurs, network topology changes and capillary diameters are slightly adapted in response to hemodynamic and metabolic stimuli. Steps (2)-(6) are repeated until the end of the simulation or until all patent capillary flow paths no longer exists. If no occlusion occurs, steps (5)-(6) are repeated until a new occlusion occurs or until the end of the simulation.

### Model Outputs

#### (1) Capillary occlusion

**Fig 7:**
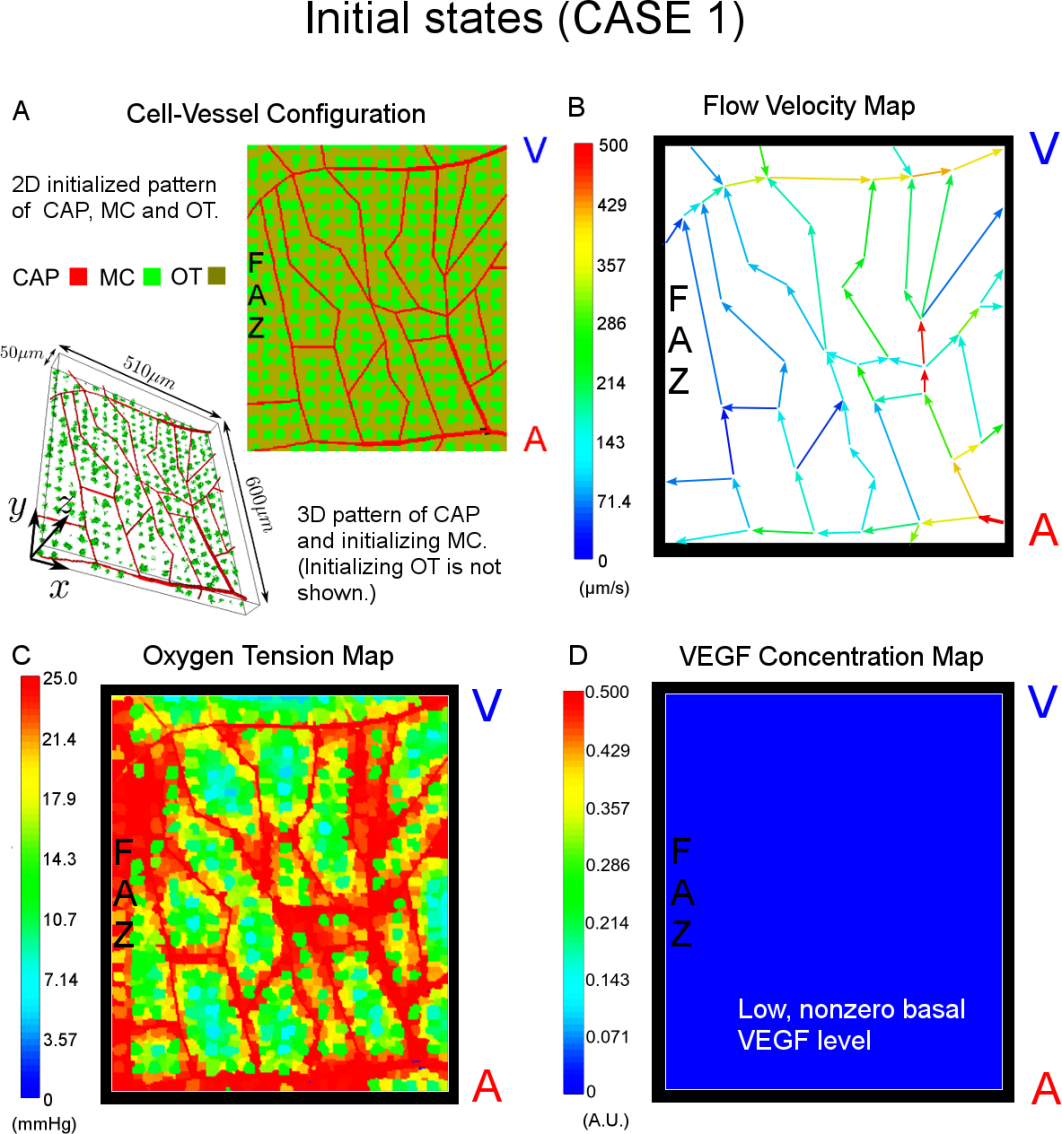
(CASE1) system configurations and field pattern under normal condition. (A) Shown in the 2D XY cross section involving capillary network, Mueller cells (green) and other retina cells (brown) are uniformly initialized between capillary segments composed of capillary blocks (red). The 3D image shows the simulated section during initialization (in the 3D completely initialized configuration, the capillary network is covered by **MC** and **OT** cells and visually inaccessible). The dimension of the simulated retinal section is 510*μm* × 600*μm* × 50*μm*. (B) Flow velocity map includes a primary arteriolar entrance (bottom right in the (B)), a main venular exit (top right), side traffic extending outside region of interest, and interconnected pathways. Capillaries closer to the FAZ (left in the figure) carry blood flow with relatively smaller velocity. The unit for velocity is μm/s. (C) Oxygen tension is highest near capillary segments and the FAZ. The more distant a cell is from irrigating capillaries, the lower is its oxygen level. The unit for oxygen tension is mmHg. (D) VEGF levels are initially assumed to be at a low but nonzero basal concentration across in the whole area. VEGF level has arbitrary unit. “FAZ refers to foveal avascular zone, “A” refers to arteriole, and “V” refers to venule.

The model starts with the cell-vessel configuration show in Fig 7A. In order to visually track occlusions of the capillary network, blood flow pathways are shown using a color map at each time point (Fig 7B). Additionally the model calculates the average minimal cell-to-vessel distance *d*^*min*^, which measures the distance from a Mueller cell to its closest patent, or non-occluded, capillary segment as a system output.

#### (2) Ischemia

Oxygenation of the retina is visualized using a color map in which the spatial distribution of oxygen is presented and ischemia is highlighted in blue (Fig 7C). In addition, our model takes advantage of the average oxygen tension of all Mueller cells as a global metric of the oxygen supply condition. At the same time, distribution of cellular oxygen indicates the fraction of cells that are insufficiently oxygenated, i.e. ischemic. The consequence of ischemia is local production of VEGF and produces a resulting equilibrium VEGF concentration map (Fig 7D).

#### (3) Flow

Total inflow rate is monitored in the model as system output indicating of how well the modeled section of tissue is receiving oxygenated blood. In addition, a vector map embedded on the capillary network shows the spatial distribution of flow velocities which are color coded.

#### (4) Retinal thickness

A color coded profile of retinal thickness, acting as a retinal edema surrogate, is monitored with the progression of the occlusive process. Retinal thickness simply refers to magnitude in Z axis of simulated section (Fig 7A).

## Results

### Spatial patterns of disease progression

It is important to maintain perspective regarding the model. While we initially present results in terms of an example run of the model, CASE 1, as explained below, the model was run many times to explore the impact of the implemented stochastic events.

The configuration of cells and vessels has been initialized for a sector near the FAZ with dimensions of 510*μm* × 600*μm* × 50*μm*, as determined from a patient ASOLO image. The sector during initialization is viewed in 3D with **CAP**s and **MC**s visualized (Fig 7A). Completely initialized configuration shows that **MC**s and **OT**s are uniformly patterned between vessels from a 2D view (Fig 7A). Under normal conditions, the blood flow is sourced from the arteriolar entrance, flows through the capillary network and exits via the venule. Because the model concentrates on a small arteriole-venule sector, side streams reflect capillaries connecting neighboring peri-foveal networks (Fig 7B). Capillaries near the FAZ carried a relatively small blood flow, and therefore had a higher probability of occlusion based on the assumed occlusion mechanism. Cells near capillaries had abundant oxygen supply, while more distantly situated cells received less, but still sufficient, oxygen to support normal activities without ischemia (Fig 7C). In the initial state VEGF production was assumed to be in the basal diabetic physiological range when retinal tissue was adequately oxygenated (Fig 7D). This corresponds to a slight elevation of VEGF above the basal non-diabetic state; an elevation sufficient to induce the presence of a low level of ICAMs allowing diabetes induced leukostasis.

**Fig 8:**
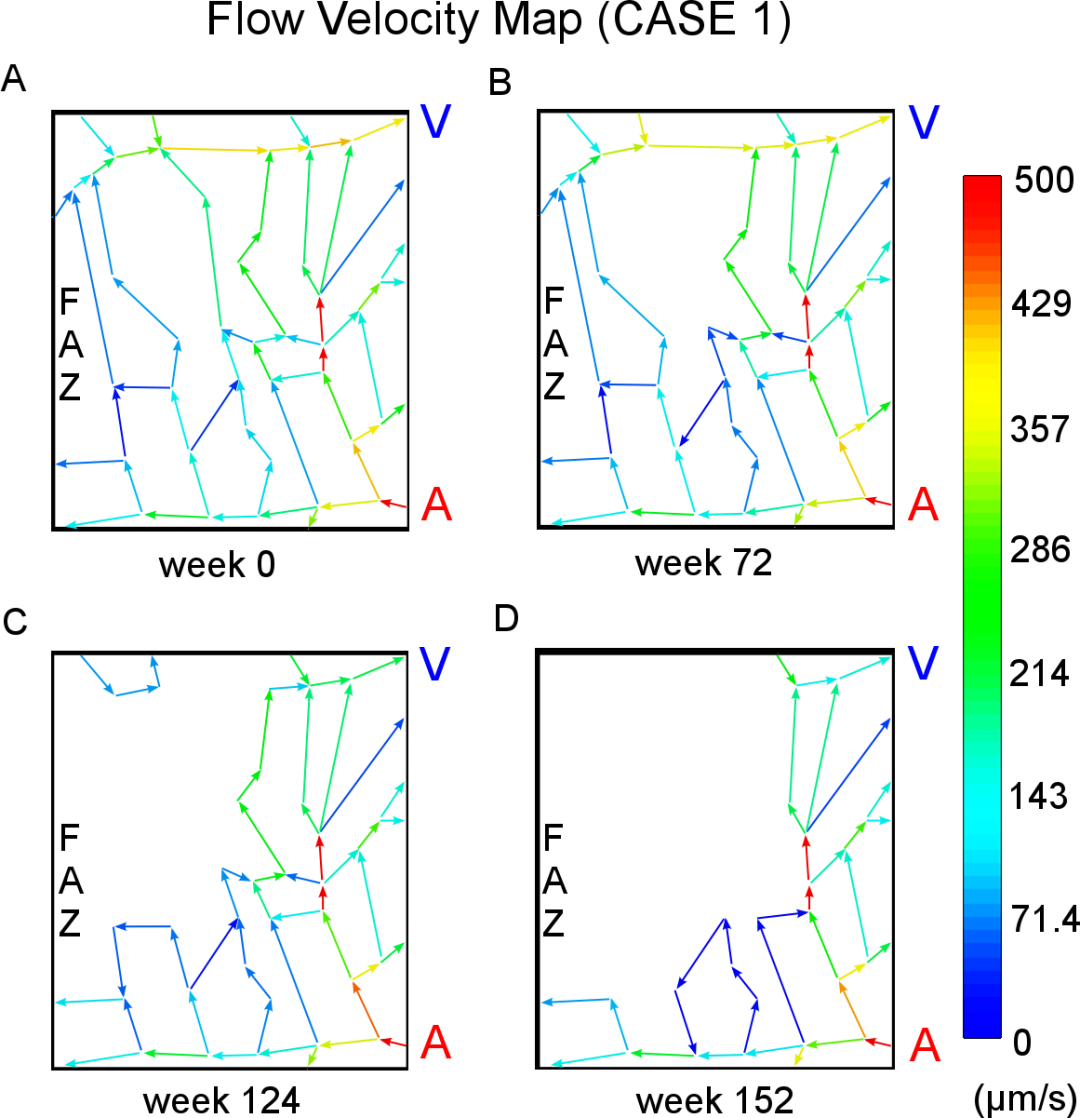
(CASE1) flow velocity pattern following initial capillary occlusion. (A) The flow velocity map captures loss of a flow pathway due to capillary occlusion in week 0. (B) The second capillary spatially close to initial occlusion site became occluded in week 72. (C) Several capillaries near FAZ and venule became occluded in week 124. (D) More than a quarter of the capillary network was obstructed by week 152. Color and the direction of arrows reflect the magnitude and orientation of velocities respectively. The redder the color is the greater the flow velocity. The unit for velocity is μm/s. “FAZ” in the figure refers to foveal avascular zone, “A” in red refers to arteriole, and “V” refers to venule.

**Fig 9:**
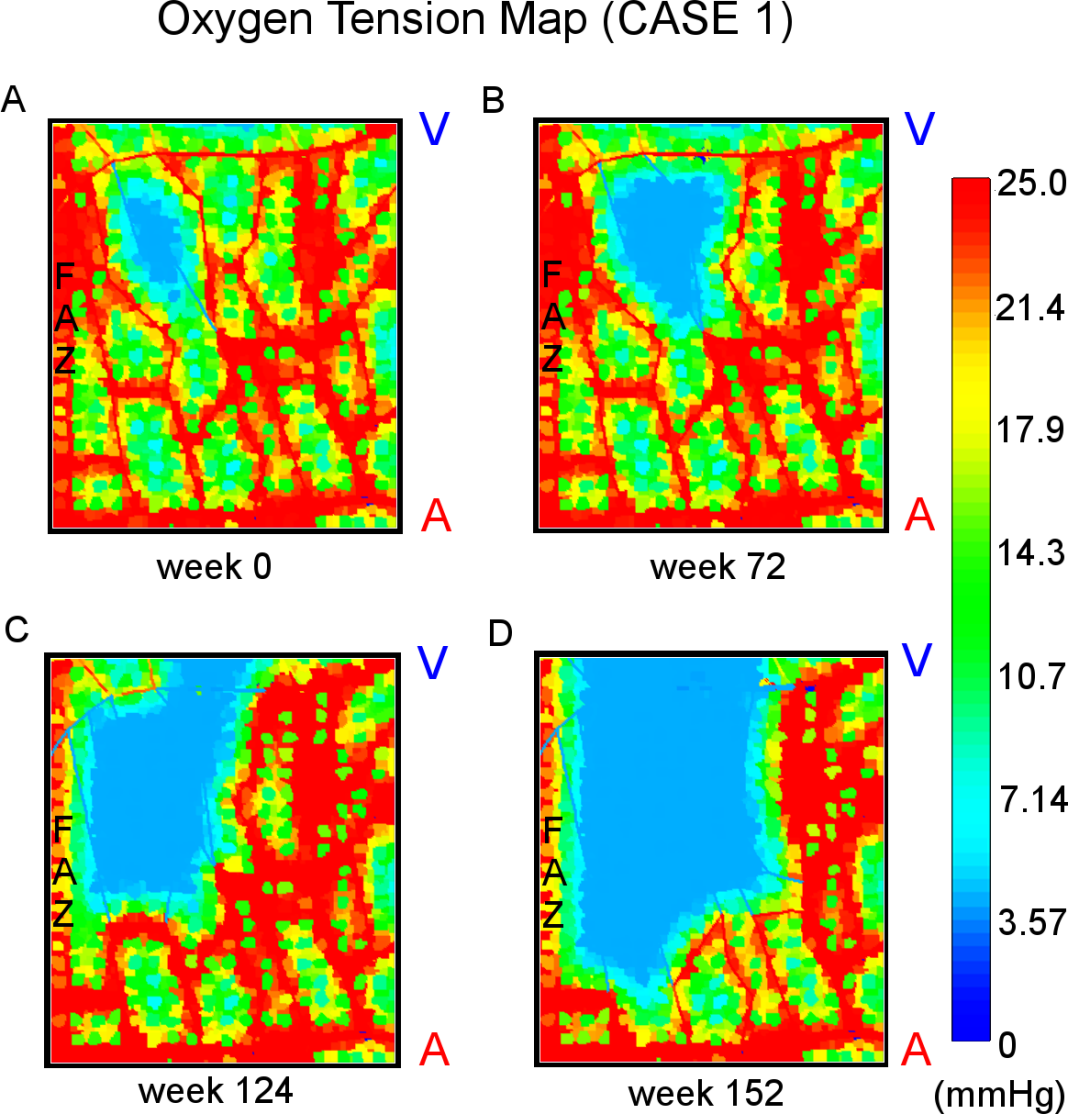
(CASE1) oxygen tension pattern following initial capillary occlusion. (A) Oxygen tension map shows a localized hypoxic region near the occluded capillary in week 0. (B) Hypoxic area of cells broadened to enclose a second occlusion site in week 72, but it’s still restricted and confined spatially to the arteriole-venule sector. (C) Local insult by hypoxia propagated to break the terminal capillary near the venule and extended to the neighboring AV sector in week 124. (D) Large area of hypoxia was observed in week 152. Color reflects magnitude of oxygen tension. The redder the color the higher the oxygen tension is. The unit for oxygen tension is mmHg. “FAZ” in the figure refers to foveal avascular zone, “A” in red refers to arteriole, and “V” refers to venule.

**Fig 10:**
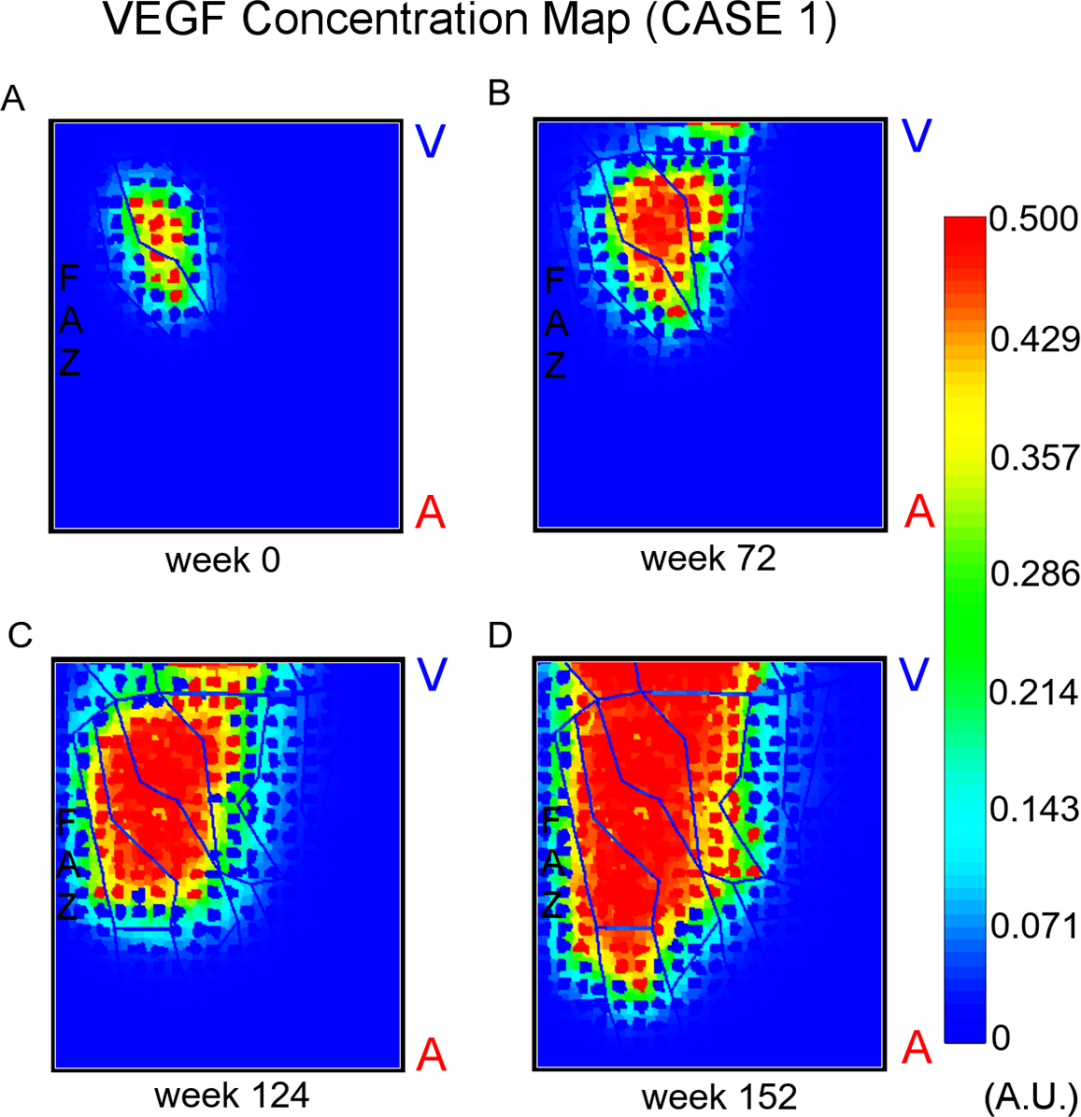
(CASE1) VEGF level pattern following initial capillary occlusion. (A) VEGF level map shows localized synthesis of VEGF by Mueller cells in response to hypoxia in week 0. (B)—(D) Increasing number of Mueller cells actively produced VEGF in weeks 72, 124, and 152, where the pattern of regions with high VEGF reproduced that of the area with low oxygen tension. Color reflects magnitude of VEGF level. The redder the color the higher the VEGF level is. VEGF level has arbitrary unit (A.U.). “FAZ” in the figure refers to foveal avascular zone, “A” in red refers to arteriole, and “V” refers to venule.

Once a first occlusion was initiated, the network topology changed reflecting the loss of that capillary and the steady state of oxygen tension and VEGF level were reestablished accordingly (Figs 8–10). In week 0, a capillary was occluded (Fig 8A) and consequently tissue surrounding it became poorly oxygenated (Fig 9A). Within the model, the date of first capillary occlusion is always week 0. This is not the date of the initiation of diabetes. Certain Mueller cells become ischemic from this capillary occlusion and these hypoxic Mueller cells produce and release VEGF, which then develops local concentration peaks (Fig 10A). Adjacent capillaries respond to the elevated concentration of VEGF, which increased their risk of occlusion via the ICAM mediated leukostasis mechanism not addressed separately in the model. Due to limited diffusion length of VEGF, distant capillaries are insensitive to such localized change. In week 72, secondary occlusions took place and the flow network became increasingly impaired (Fig 8B). Consequentially, more Mueller cells had an insufficient oxygen supply and produced increased levels of VEGF (Figs 9B and 10B). At this point, the capillary damage appeared well bounded and confined in one arteriolar-venular sector. Notably, several cells in a neighboring area became hypoxic and produced elevated VEGF, possibly because their nearest vessel lost an important upstream branch, which carried significant oxygen supply before closure. However, there were still barriers composed of healthy cells and capillaries between the adjacent sectors. There then followed a series of capillary occlusions with minor enhancement in total volume inflow rate, until the damage in week 124 resulted in drop of inflow rate the first time since onset of initial injury (see below).

Concomitant with the drop in total flow, the terminal venule between the two sectors was compromised (Fig 8C top of image) and a larger fraction of tissue was now hypoxic (Fig 9C). In week 152, capillaries near the FAZ and the terminal venule were no longer patent (Fig 8D), the FAZ is considerably enlarged, and roughly one third of the tissue was hypoxic and had an elevated VEGF environment (Figs 9D and 10D). We note that Sakata *et al*. 2006 [118] using a fluorescein angiography technique to measure perifoveal capillary blood velocity were able to show a significant negative correlation between capillary blood flow velocity and the size of the foveal avascular zone in diabetics without edema. In the model, capillaries bordering the avascular zone show slowed flow consistent with Sakata *et al*. results [118].

### Edema formation

**Fig 11:**
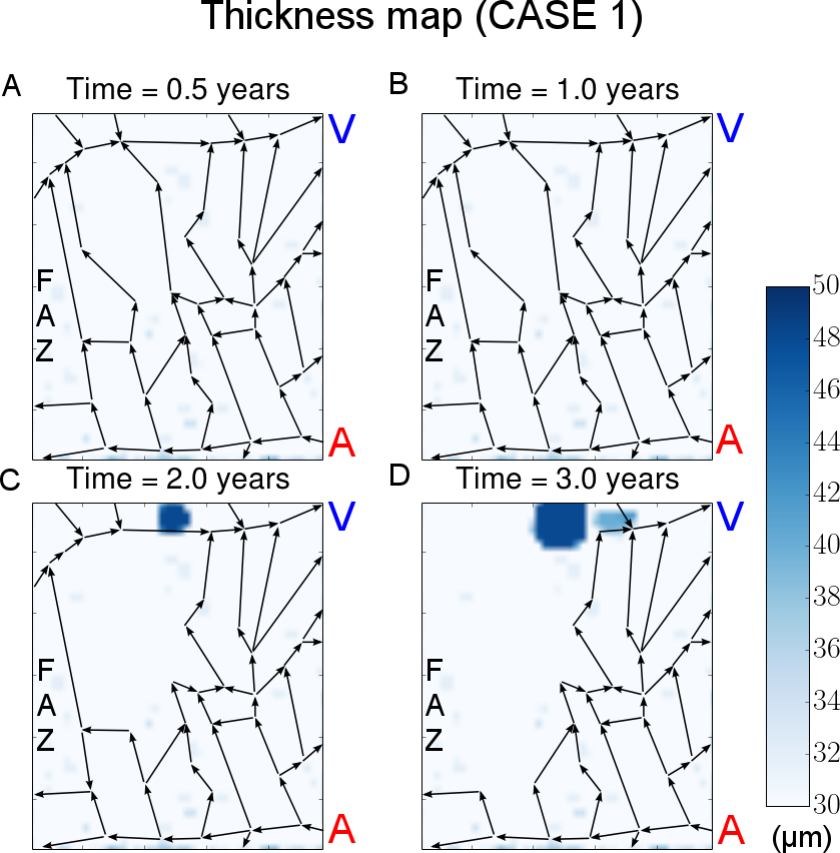
(CASE 1) retinal thickness over time. Thickness of the retinal layer is represented by a color map at the end of year 0.5 (A), year 1 (B), year 2 (C) and year 3 (D). Color represents the magnitude in Z axis. The bluer the color, the thicker a local retinal area is. Fluid appeared between year 1 and year 2, at the venous edge of the area of occlusion. The flow network is overlaid upon the color map to present patent flow paths at the time point of observation. Color bar only represents the thickness of tissue but not the flow velocities. “FAZ” in the figure refers to foveal avascular zone, “A” in red refers to arteriole, and “V” refers to venule.

Based on simple assumptions on the mechanism of edema formation (detailed in **Edema Formation** section in S1 Text), our model showed local retinal thickening (Fig 11). A significant volume of fluid was observed 3 years post onset of initial capillary occlusion (Fig 11D). Extravascular fluid was responsible for the increase in retinal thickness. Moreover, the spot where fluid was present correlated with the boundary of the ischemic region. The current study is limited to qualitative illustration of how retinal thickening can be caused by abnormal VEGF synthesis by Mueller cells. This is the canonical function of VEGF producing leakage from capillaries. In the model edema is only a function of threshold VEGF levels and not a measure of local Starling type relationships. It is reflective within the model of loci of active leakage at sites of intact, non-occluded capillaries which also have elevated VEGF above a particular threshold. Edema within the model is a prediction of expected locations of either retinal thickening or of retinal cystic structures as seen clinically on ocular coherence tomography. The model does not address the actual range of cysts since these can often be present in areas of retinal ischemia lacking active leakage by fluorescein angiography [12] and thus can represent processes other than active leakage such as cellular necrosis or impaired retinal pigment epithelial pumping. The model does leave cysts present in areas in which capillaries had leaked but then subsequently become occluded similar to what occurs clinically.

### Quantitative global measurement of disease progression

**Fig 12:**
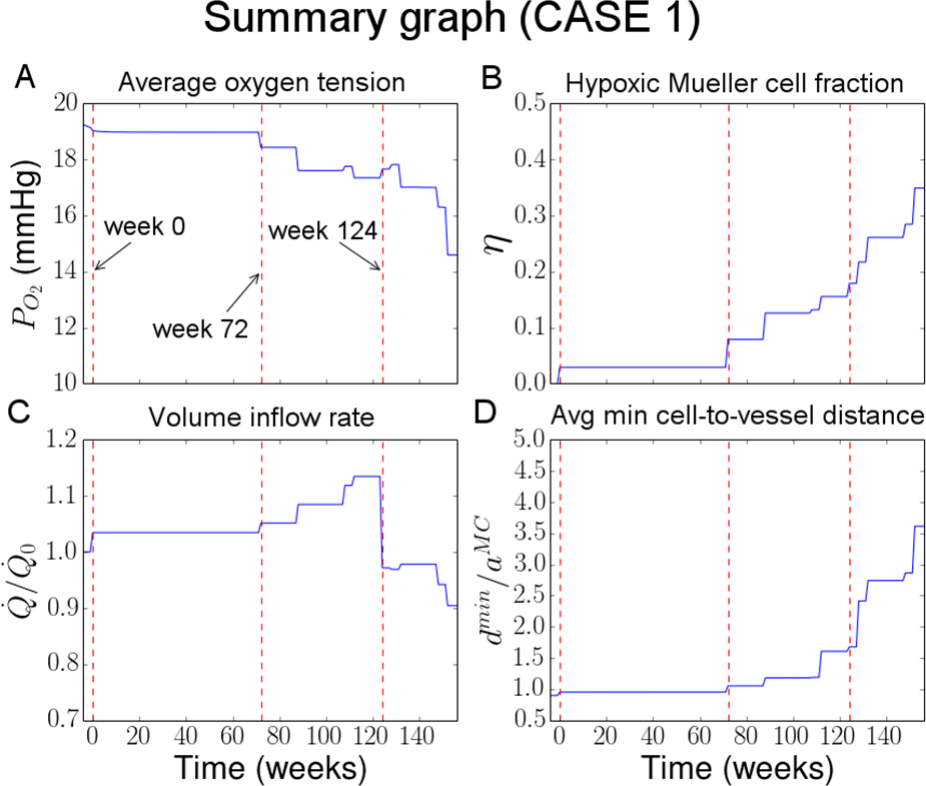
(CASE 1) quantitative development of model properties with time. (A) Average oxygen tension decreased with increasing occlusion events despite occasional small increases, and had an overwhelming drop near the end of simulation. (B) The hypoxic fraction of Mueller cells was observed to grow rapidly after the capillary occlusions induced by locally elevated VEGF, and near the end of simulation about one third of the Mueller cells were ischemic. (C) Total volume inflow rate established a rising trend before a major decline in week 124, after that it remained at a lower level than the starting point and eventually descended to below 90% of initial rate. (D) Average minimal cell-to-vessel distance maintained an increasing trend to reach 3.5 a^MC^ (Mueller cell diameter) units, which qualitatively reproduced the temporal pattern observed for hypoxic fraction of Mueller cells.

Following the onset of capillary closure, almost every additional occlusion caused average oxygen tension within cells to drop (Fig 12A). The fraction of hypoxic Mueller cells went up nearly linearly from week 72 to week 152 (Fig 12B). Distinct from the monotonic change in average oxygen tension and hypoxic fraction, the total volume inflow rate was non-monotonic, with continuous increases before a sharp decrease in week 124 and one more in week 152 (Fig 12C). Early stages of increased inflow rate might be attributed to physiological response to loss of a selected group of blood flow pathways. Because oxygen tension is assumed to be constant within inlet capillary blocks, as would be physiologically expected, a higher volume inflow rate gives more oxygen carried into the system within a given period of time. This might suggest that at early stages of the disease with few capillary closures, the system would compensate for the oxygen insufficiency by increasing the blood flow. In contrast, at later stages of disease when many capillaries connecting the arteriole to the venule were occluded, total flow declined and occlusions seemed to occur more frequently. To better map the model to clinically observed symptoms, our model used the scaled minimal cell-to-vessel distance *d*^*min*^ / *a*^*MC*^ as a metric to quantify disease progression spatially, where *a*^*MC*^ is the typical size of a Mueller cell (Fig 12D). This is basically the number of Mueller cell diameters to a patent, or unoccluded, vessel. The *d*^*min*^ / *a*^*MC*^ increased monotonically with each additional capillary closure. Interestingly, the capillary occlusion that triggered a rapid drop of the total volume inflow at week 124 had a mild effect on *d*^*min*^ / *a*^*MC*^. This implied that topological location of a capillary segment was influential to the system blood flow supply, which might be neglected in a spatially-based metric. Thus, a flow-based model may be helpful in understanding the critical point at which the disease starts to evolve in a different way i.e. toward a decline in total inflow.

**Fig 13:**
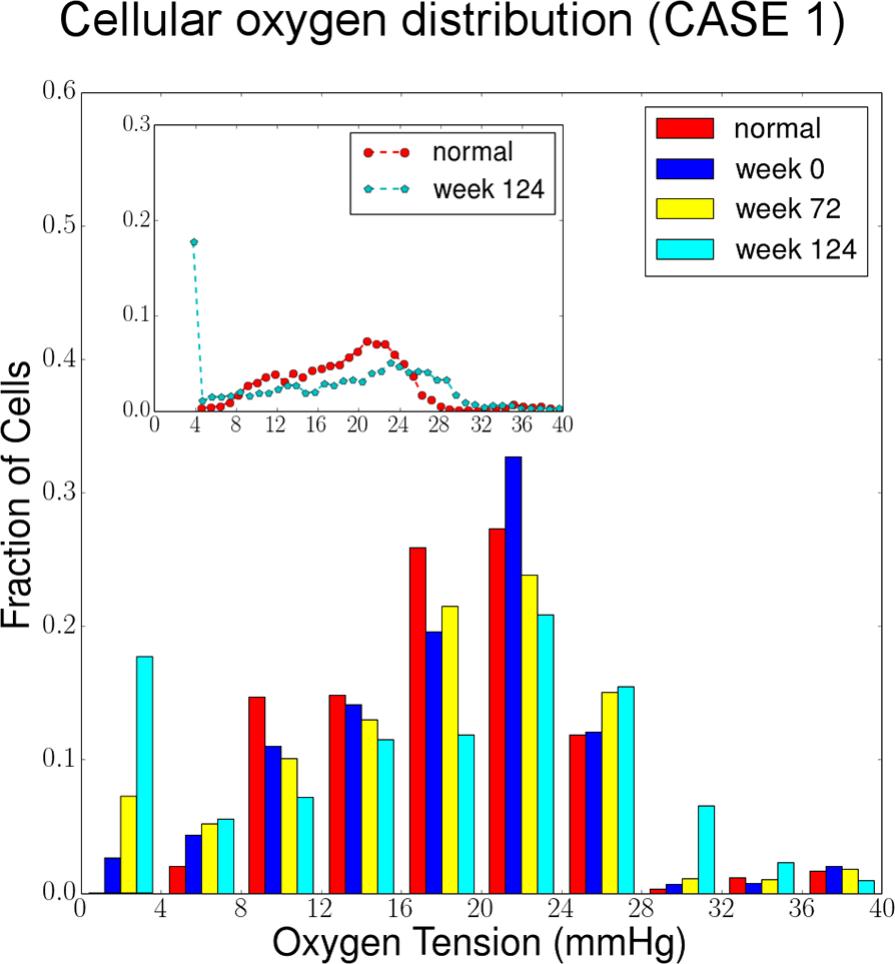
(CASE1) cellular oxygen distribution over time. Fraction of cells in each 4 mmHg oxygen bin is shown for the normal condition and 3 additional times. The distribution of oxygen tension within all cells exhibited an essentially unimodal shape under the normal condition (normal-red bars) where most cells had oxygen tensions of 10 to 25 mmHg, a small portion of cells located near vessels had higher levels ranging from 35 to 40 mmHg and no cells had an oxygen tension less than 4 mmHg O_2_. Capillary occlusions induced by VEGF gradually altered the distribution (week 0-blue bars, week 72-yellow bars, week 124-cyan bars). An increasing number of cells turned hypoxic. The broad peak of cells at moderate levels of oxygen decreased and broadened with more cells both at lower oxygen levels with each successive interval and more cells from about 25–30 mmHg in each successive interval. The cell oxygenation distribution gradually morphs from a unimodal distribution to a bimodal oxygenation distribution. Inset figure shows a comparison between normal condition and week 124 using a line-connecting-dot presentation, with finer oxygen tension spacing between two consecutive data points. The inset figure also strikingly shows the transition from unimodal to bimodal distribution as well as peak decreasing and broadening pattern at moderate levels.

The distribution of oxygen tension within all cells exhibited an essentially unimodal shape under the normal condition where most cells had oxygen tensions of 10 to 25 mmHg, a small portion of cells located near vessels had higher levels ranging from 35 to 40 mmHg (Fig 13) and no cells had an oxygen tension less than 4 mmHg O_2_, i.e. no cells were ischemic. Capillary occlusions induced by elevation of VEGF gradually altered the distribution. An increasing number of cells turned hypoxic. The broad peak of cells at moderate levels of oxygen decreased and broadened with more cells both at lower oxygen levels and more cells from about 25–30 mmHg. This suggests that while the size of the hypoxic region in this tissue section was growing larger more cells were exposed to high oxygenation producing a bimodal oxygenation distribution.

This model’s results are important both in terms of the images produced which bear a striking resemblance to those seen clinically and also as summarized in graphs showing changes occurring over time in a single run of the model for a specific initial capillary closure (Figs 8–13). Are these images and graphs consistent with what is known clinically about diabetic retinopathy? The pattern of capillary loss near the FAZ with expansion over time is very similar to what is seen in diabetic patients. The pattern of mixed ischemia and edema in the perifoveal area is also that usually seen clinically. The curve in Fig 12C is the most interesting in that flow rises from baseline levels for a period of time by as much as 13 percent and then declines. Clearly in end stage diabetic retinopathy with the entire capillary network occluded, the flow will go down. It is less clear that earlier stages in loss of the capillary network will result in increased total flow. This occurs because the model has some dilation of capillaries as a consequence of the adaptation module which would increase flows and also partially due to changes in network structure. The literature on blood flow in diabetes has been inconsistent due to measurements on diabetics at different stages of disease with a number of different technologies imaging flow in different locations [119]. The data from the Retinal Function Imager (RFI) measures flow velocities in small perifoveal vessels and seems most comparable to the vessel sizes in the model. This data is consistent with the model in that it shows an increased retinal blood flow velocity in diabetic patients without clinically seen morphological changes [119]. This would be similar to patients in the first year or two of the simulation (approximately 100 weeks).The percent increase in blood flow over controls was about 15% in [119], quantitatively similar to the model’s results. Physiologically this is possibly secondary to increased vasodilator mechanisms due to tissue hypoxia [120] and increased nitric oxide synthase [121] and possibly network changes which could have occurred even though the patients did not have clinically visible changes. The model replicates this effect though it does not explicitly utilize any analogous mechanisms. This pattern of increased macular blood flow accompanied by edema (Fig 11) is consistent with the regional distribution of diabetic lesions emphasized by [28]. Burgansky-Eliash 2010 has RFI data on patients with non-proliferative diabetic retinopathy [122] which show decreased blood flow at this stage of clinical retinopathy comparable to the blood flow decrease seen in the later weeks of this simulation. There is also clinical data on venous oxygen saturation in diabetes [123] showing that venous oxygen saturation in diabetes is elevated over that seen in normals. This model did not specifically treat venous oxygen levels but as blood flows were rising with a constant input oxygen saturation occurring simultaneously with a rise in hypoxic retinal cells, retinal oxygen extraction must be lower resulting in elevated venous oxygen saturation. The oxygen map shows this qualitatively as an expanded reddish tissue area (top right corner near “V” Fig 9C vs 9A) of elevated oxygen saturation around the venule. Hammer *et al*. (2009) interpret this elevation in venous oxygen levels to be due to a shortened arterio-venous passage time resulting in reduced oxygen extraction [123].

### Replicate simulations

**Fig 14:**
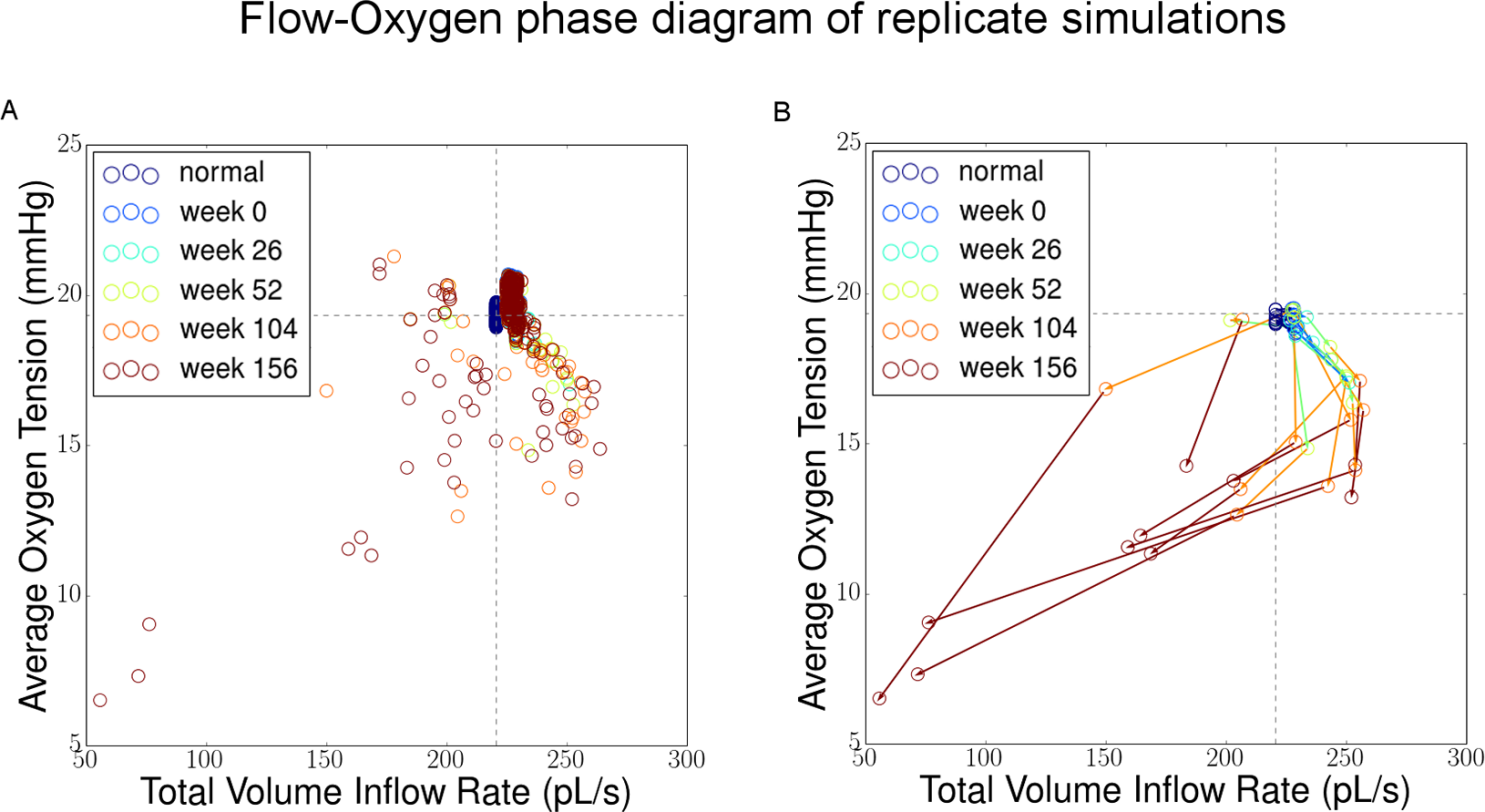
(CASE 1) Flow-Oxygen phase diagram of 362 replicate simulations. Flow-Oxygen phase diagram describes how total volume inflow rate and oxygen tension evolve with time in all replicate simulations. Color represents simulation stages where bluish circles stand for early stages and reddish ones correspond to later stages. (A) Many simulations show clustering of flow-oxygen states through all monitored time points. Those clusters, quite close to the equilibrium state under normal condition, remained overlapped post the first occlusion. On the contrary, a few simulations displayed a scattered pattern of flow-oxygen states near the end of the simulation with either reduced flow or oxygen tension or both. (B) Temporal trajectories of the scattered points shown in A whose states had either less than 75% initial inflow rate or oxygen tension in year 3, were considered progressive. Simulations that lead to the most severe propagation of capillary occlusions tend to end with both lower than normal flow rates and lower oxygen tension.

We performed 362 replications of the macular capillary sector simulation on Indiana University’s supercomputer BigRed II to pursue the consequences of different initial occlusion sites and explored the evolution of progression states in a flow-oxygen phase diagram (Fig 14). The probabilistic aspect of the capillary occlusions means that repeated runs of the model will not produce identical patterns of capillary loss but similarity of replications will be strongly influenced by network structure. In the flow-oxygen phase diagram, the normal condition (circles in dark blue) were clustered in a confined range, which showed that all simulations had similar equilibrium oxygen tension and total inflow rates initially as expected (Fig 14A). Circles gradually became scattered, because different simulations randomly picked different occlusion sites resulting in different disease progression trajectories. At 156 weeks, most simulations landed not far from the initial oxygen-flow states, which corresponded to situations without derived occlusions. On the contrary, some simulations showed distant end-point states situated mainly in low oxygen territory, which represented exacerbating progressive capillary occlusions. Of all such simulations, temporal trajectories were visualized (Fig 14B) if the simulations showed less than 75% total inflow rate or oxygen tension in year three. Most such simulations followed a clockwise trajectory temporally, starting from equilibrium state, transiting via low-oxygen and high-flow zone and ending in the low-oxygen and low-flow territory of the phase diagram, which indicated a severely damaged end-point of the capillary network. The rest seemed to pursue a temporal pattern of evolution but still remained in a transition stage. These graphs show the results of typical runs of the model. This might illustrate a general picture of how retinopathy progresses in terms of system oxygenation and total blood supply. Note that clinically many diabetics do not develop clinically significant retinopathy whereas a minority has significant propagation of capillary closure. This model would indicate that this variability of progression could have a significant element of probability, ‘bad luck’, in terms of which capillary was initially an occluded, in addition to other aspects of diabetic control and genetic/epigenetic factor [124–126]. It is now possible to gather time sequence data on the macular capillary network structure of diabetics through the utilization of AOSLO and in particular to do so in the two eyes of single patients, which possess different network anatomies but presumably identical environments in terms of glucose, blood pressure, and levels of systemic leukocyte activation. These data will allow comparisons of patient capillary occlusions and edema over time with sequentially constructed model based probability occlusion maps. This ought to allow refinement of model parameters or rejection of the model.

**Fig 15:**
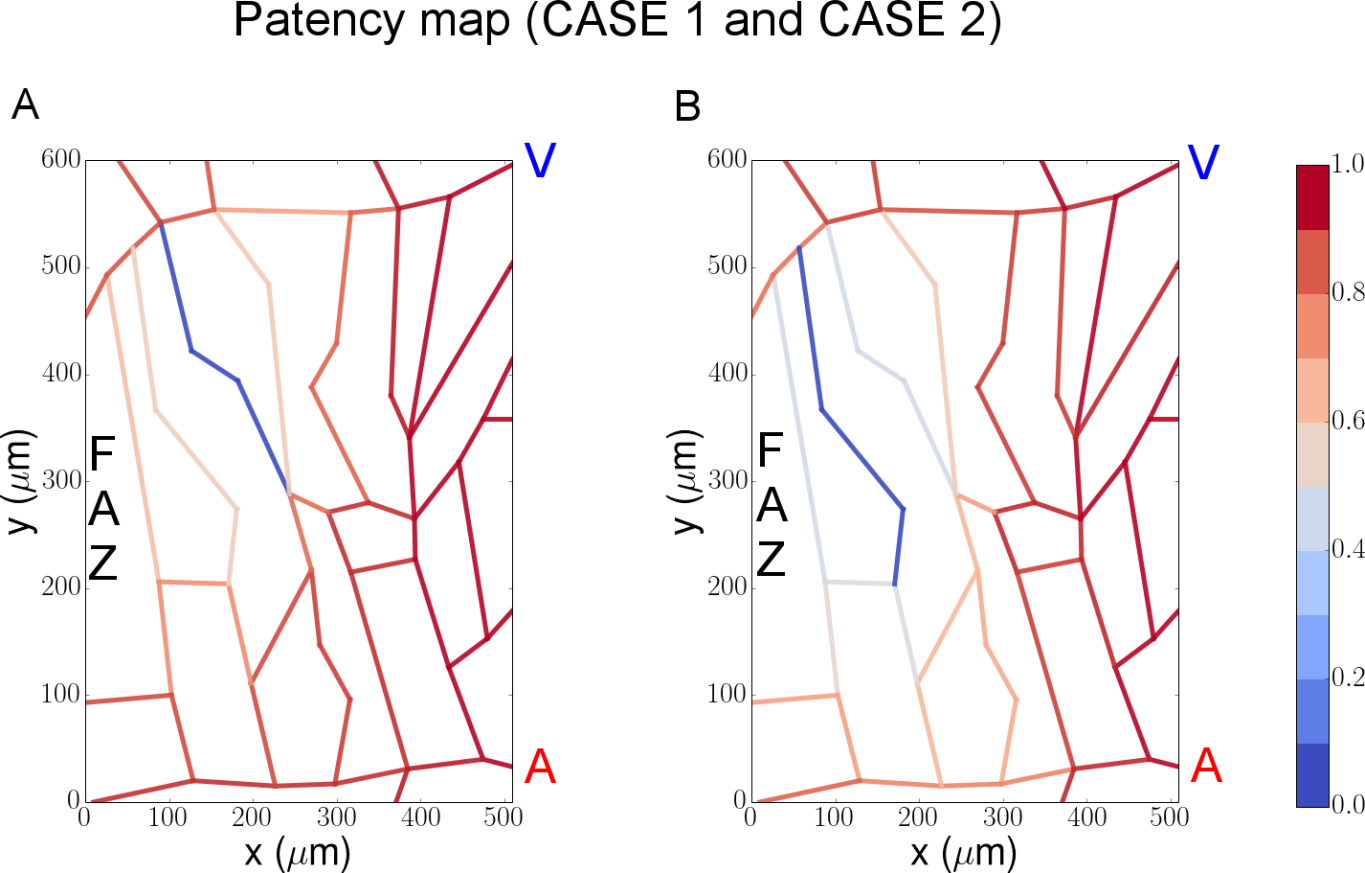
Patency map of the macular capillary network. The patency map illustrates the probability of each capillary segment remaining open given a specific initial capillary closure. These data are 2 subsets of the 362 replicate simulations in which 2 specific capillaries were occluded. (A) Patency map following CASE1 initial occlusion site shows that a high probability of secondary occlusions is limited to adjacent capillaries, largely those closer to the FAZ (n = 45). (B) Patency map following CASE2 initial occlusion site shows greater probability of broader propagation of injury (n = 30). Color represents the frequency of a capillary segment being patent after 3 years of simulated time since the initial occlusion. Warmer color corresponds to less vulnerability to occlusion, or higher capillary patency.

We further summarized the vulnerability of the capillary network given a certain initial occlusion site (Fig 15). Among all capillary segments carrying relatively slow blood flow, initial occlusion of two capillaries near the FAZ seemed to be most influential in triggering derived occlusions, while occlusions of others had a less significant impact. Both cases showed a spatially relevant patency distribution, with capillaries closer to the initial occlusion site bearing a higher frequency of occlusion. Not uncommonly, capillaries near the terminal FAZ venule and arteriole were also candidates of occlusion. Closure of these capillaries was likely to propagate injury to neighboring foveal arteriolar-venular sectors. These runs show similar results and show that certain portions of this subject’s capillary network are vulnerable to occlusion whereas others seem to be more resilient. Those more resilient areas tended to possess more densely situated capillaries and therefore the area of ischemia produced by a capillary occlusion is small and consequentially so is the resultant increase in local VEGF production and the probability of propagation. Occlusion or survival of a capillary is highly influenced by the local capillary network structure. This phenomenon seen in these results may be the result of more dense capillary networks further from the fovea where the visual impediment consequent to blood vessel opacity is less significant perhaps allowing more closely spaced capillaries. This pattern of loss of perifoveal capillaries, clinically called enlargement of the FAZ, is a commonly seen clinical pattern of macular ischemia development [11].

### Model improvements

Experimental data of a number of types can improve and validate the model. Arteriolar and capillary blood flow velocity data is needed to calibrate the network flow module and adjust the structural adaptation module. There are currently AOSLO based [127, 128] and ocular coherence tomography (OCT) angiography techniques [129] that will provide valuable inputs to future diabetic retinopathy modelling but no published techniques for measuring capillary blood velocities are sufficiently mature for routine use. Improved vascular imaging with capillary structures determined over the several retinal capillary layers in the macula would help extend the model beyond its current limitations. With regard to retinal edema, thickness projections can be validated and refined against OCT macular thickness maps and hydrostatic as well as oncotic pressures can be incorporated into the model. Refinements of the relationship of local retinal edema and capillary loss with visual functioning are also on the horizon [130] and can be assessed over time. More detailed structural modeling can be done of the cystic structure of the usual diabetic macular edema as can be determined by OCT. In the periphery the model predicts a gradual enlargement of the sorts of capillary free dark areas commonly seen on fluorescein angiography with occasional development of new small areas of capillary loss that also gradually expand and are blocked, at least temporarily by the oxygenated areas surrounding larger retinal vessels. Sequential fluorescein angiography, especially wide field angiography, would be validating in this area and allow refinement of the occlusion probability functions used in the model.

## Discussion

We have introduced a conceptually simple though computationally intensive model that often, but not always, produces a spreading area of capillary occlusion and retinal ischemia after an initial capillary occlusion within a defined capillary network. This model was largely coded in Python with partial implementation in the Compucell3D environment [98]. The paper’s chief aims were to model the progressive loss of capillaries in diabetic retinopathy as seen in both diabetic maculopathy as well as in peripheral retinal ischemia (see S4 Text). This simple model replicated several clinically observed features of diabetic retinopathy. The first being the progression of capillary loss once a single capillary has been occluded, though the model often runs for a long period prior to any ‘spontaneous’ occlusions. This is quite similar to the clinical situation in which it takes a number of years after the diagnosis of diabetes before the first retinopathy is clinically evident. The presumed physiology is that even though the diabetic patient has upregulated endothelial ICAMs and activated leukocytes very early in the disease, the leukocyte adhesion process which can then kill an endothelial cell has to act locally many times before the endothelial population is replicatively depleted and capillary occlusion can occur. Once an occlusion has occurred in the macula there is often but not invariably progression of capillary drop out to produce an area of ischemia. Often the loss of capillaries adjacent to the foveal avascular zone occurred early in the modelling process giving the enlargement of the FAZ, a common feature seen in diabetic maculopathy [11]. The pattern of macular edema produced by the model with active leakage at the edges of an ischemic area is similar to that clinically seen in diabetes. The model generates edema at the edge of areas of progressive capillary loss which can then be present within an area of capillary occlusion as the size of the ischemic area enlarges [118].

The model treatment of the peripheral retinal capillary network (see S4 Text) without model alteration except as to appropriate retinal capillary network structure, peripheral retinal capillary diameter and arteriovenous pressure difference, produced rapidly progressive loss of the ladder capillaries seen in the retinal periphery with preservation of the arterioles, venules and peripheral shunt vessels (S9–S11 Figs). This is again similar to the clinical picture. The model showed apparent barrier function of intact arterioles and venules as the progressive capillary occlusion does not generally cross to an adjacent sector but progresses within an arteriovenous sector. In addition, the peripheral model, unlike the macular model, did not generate significant retinal edema, a result that is strongly reminiscent of the clinically seen condition [28].

The model’s system summary graphs for the macular sector are particularly valuable since they clearly demonstrate global features of the evolution of damage and can therefore be compared to experimental data [119, 122]. The changing flow in the macular area over the time course of the model is quite consistent with these experimental results showing an early hyperemic state followed later by decreased macular blood flow. The model is also revealing in the comparison of the macular flow to that in the retinal periphery. The peripheral flow shows a progressive decline with time, reflecting an ischemic retinal periphery, whereas the macular flow is increased, hyperemic, for much of the modelling period. This is consistent with the regional disparities in diabetic retinal disease emphasized by Skov Jensen *et al*. [28].

The macular model was iterated and produced different patterns of capillary occlusion on different runs (compare Figs 8–11 vs. S1–S4 Figs). The same macular capillary network could remain largely intact without propagating ischemia on some runs and propagate on others largely depending on the location and timing of the initial capillary occlusion (Figs 14 and 15). This dependence on location of initial capillary occlusion is a statement about the importance of the local network structure. Similarly there is considerable variability between various patients with clinically similar control of their diabetes. Patients can remain stable without clinical signs of retinopathy for various periods of time or can differ in their development of diabetic maculopathy with different degrees of ischemia and differing patterns and amounts of macular edema [11, 131, 132]. This may occur even between the two eyes of a single patient without detected mitigating or exacerbating factors between the two eyes [11]. To what degree this variation across patients and between the two eyes of a single patient is a consequence of developmental variations in their capillary network structures or due to purely stochastic variations of the occlusive process is not known. There are no data on these issues. One can note that the area of the macula most resistant to propagation of occlusion is that furthest from the FAZ which has the greatest density of capillaries. In this area the region of ischemic retina produced by a capillary occlusion is smallest with therefor the smallest amount of VEGF produced. Certainly one would hypothesize that a densely vascularized macula would be at lower risk for propagation of ischemia than a sparsely vascularized one. It would be important for patient prognostication as well as for medical economics to understand and predict this phenomenon of clinical variation on the basis of observable capillary anatomy. This variation in structure of the capillary network in the retina is likely determined during development by local physics such as oxygen diffusion with an influence of genetic differences as well as stochastic events. The perifoveal capillary networks can now be visualized non-invasively by imaging technology such as the AOSLO [133] and OCT angiography [129]. The AOSLO-determined structures of the perifoveal capillary network are generally similar in size between the two eyes of a single patient, but certainly they are different in details of the capillary networks. (Figure 2 in [134], also Figure 5 in [133]). The FAZ size and shape are, unlike that between the two eyes of a single person, quite variable across individuals [135]. In the clinical situation the two eyes of a diabetic are often similar but with different degrees of macular disease [11]. A long term goal for our modelling approach is to utilize technologies available to image the local capillary network and input this into a retinal vascular model of the development and progression of diabetic retinopathy to produce an estimate of risk of progression for an individual patient. At this point the model suggests an element of probability in the development of progressive macular capillary occlusion, of course as a result of its assumption that local capillary occlusions, a result of leukostasis, are probabilistic, though it is difficult to imagine how they could be deterministic. Once initial occlusion events occur, the course of progression could be more predictable. If the capillary network anatomy is the dominant determinant of risk, and the variations in occlusion probability across the area modelled from the FAZ to areas only slightly further away seem to show this, as does the hexagonal model, then this sort of model of progressive capillary occlusion may eventually become clinically valuable. It would seem helpful even if the only macular result was to predict a vulnerability of sparse capillary networks in the perifoveal area, a result which has yet to be examined. The law of large numbers may also be of assistance since modelling of the full macular capillary network would involve many more capillaries and reduce the importance of random variations in individual capillary occlusions relative to anatomical network factors such as average capillary density. Given the generally slow rate of progression of diabetic retinopathy, validation and refinement of the model requires repeated imaging of many diabetic patients over a time frame of at least several years. Given capillary imaging from AOSLO examination, the model will make predictions about the most likely pattern of capillary loss. These predictions would need to be compared to actual individual patient trajectories of capillary occlusion unlike the current paper which qualitatively reproduces several general phenomena seen in diabetic retinopathy in both the macula and periphery without confirmation of predictions in actual individual patients. Imaging on both eyes of patients could make predictions regarding the vulnerability of one eye versus the other, eliminating issues of genetics and control of blood pressure and glucose across individuals. In this situation the only significant independent variable would be capillary network anatomy. We have begun this study with sequential AOSLO imaging but have only a handful of patients more than 1 year in and this is inadequate to make generalizations at this point.

Anti-VEGF drugs are used clinically in a long-term framework to reduce diabetic macular edema and improve visual acuity and are under evaluation in the treatment of diabetic neovascularization [136]. It will be interesting to simulate the consequences of anti-VEGF intravitreal injection and explore effectiveness of protection in future modeling studies. In the model this would reasonably decrease the progression of diabetic capillary occlusion by binding to VEGF diffusing from local ischemic areas. If this model is correct in the conclusion that local VEGF levels are responsible for the propagation of ischemia, a non-canonical effect of VEGF, then earlier treatment with anti-VEGF drugs may be able to halt the progressive ischemia seen in diabetics as well as treating the edema of diabetic retinopathy. Particularly useful data in this regard would be time sequences of wide-field angiograms. These data in the absence of therapeutic intervention would allow better quantification of the function relating probability of occlusion to flow but additionally comparisons of treated and untreated patients should, according to this model, show a decreased propagation of ischemic areas as well as lowered rates of initiation of new ischemic areas in cases treated with anti-VEGF agents. Consistent with this but not utilizing this type of angiography data there is now clinical evidence that long-term ranibizumab can yield a significant reduction in progression of diabetic retinopathy [137]. Note that a significant clinical worry has been that anti-VEGF agents would increase the development of ischemia based on the canonical angiogenesis effect of VEGF. The economic costs and risks of infection associated with intra-vitreal anti-VEGF injections would likely make earlier or more prevalent injections unacceptable except in unusual circumstances but other modalities of treatment may become available. We have done modelling of the peripheral progression of capillary ischemia using alternative linear patterns of small laser burns to create oxygenated barriers to ischemic propagation analogous to the role played to some degree by the larger retinal vessels. This is quite unlike what is done with traditional pan-retinal photocoagulation. Within the model, this work shows that properly structured patterns of burns can reduce propagation in not yet ischemic areas of retina with smaller total burn area than pan-retinal photocoagulation.

### Comparison to a non-diabetic retinal vascular occlusive disease

Why does diabetic retinopathy progress to extensive areas of ischemia whereas certain other ischemic retinal vascular conditions do not progress? An example would be the arteriolar occlusions seen from particulates in IV drug users. The distinction between diabetic retinopathy and arteriolar occlusions caused by particulates is in the nature of the systemic inflammatory state in diabetes. This is likely the explanation for the non-progressive areas of capillary loss seen in certain retinal vascular conditions such as talc retinopathy in the IV drug user [138] in distinction to the enlarging areas of capillary occlusion in diabetic retinopathy. Diabetics have both a systemic inflammatory condition causing induction of cell adhesion molecules on leukocytes as well as induction of cell adhesion molecules throughout the retinal vasculature. The arteriolar occlusions seen in the IV drug user are local and likely do, through the creation of local ischemia, elevated local VEGF and induce ICAM receptors on local endothelial cells but do so without alterations in leukocyte cell receptors as the systemic inflammatory state is lacking. Therefore there is only local non-propagating retinal ischemia in the IV drug user and in animal embolic arteriolar occlusion models of diabetic retinopathy [139]. The absence of progressive capillary occlusion in either talc retinopathy or embolic models supports a ‘two-hit’ situation in diabetes, with induction of appropriate receptors on both the retinal endothelial cells and on the circulating leukocytes. Similar reasoning can be applied to the sharply geographically constrained ischemic damage produced by branch retinal vein occlusion.

The degree to which generalizations can be made to other organ systems is unclear but the presence of a positive feedback between the loss of a capillary and the likelihood of adjacent capillary loss may be less applicable to those organs which lack the constraint of a minimally sufficient, i.e. critical, vascular supply, imposed on the eye by evolution. This may partially explain the relative vulnerability of the eye in diabetes to the propagation of capillary occlusions and to the development of diabetic retinopathy.

A model provides a framework within which to analyze and interpret data. Currently, there are no appropriate models for either the progression of peripheral ischemia in diabetics or for diabetic macular disease. This model provides a structure within which to analyze and compare diabetic retinal vascular disease and some framework even if it proves incorrect or inadequate is required to move the understanding of this area forward in an era of refined imaging techniques able to provide inputs at the level required for capillary network modelling. This model, may, in some future instantiation, after considerable refinement and validation, allow improved treatment, with personalized prognosis, from imaging of a diabetic patient’s capillary network and with subsequent modelling allow a prediction regarding risk for vision impairment. Those at especially elevated risk may be candidates for earlier intervention either from anti-VEGF agents or from an alternative approach to photocoagulation. It seems reasonable that this sort of model could influence the course of patient therapy possibly in the not distant future.

### Limitations

The current model is clearly limited relative to actual retinal vascular anatomy and physiology. The model was based on the advection and diffusion of the simplest known limiting factor, oxygen, rather than dealing with, say, removal of lactate or carbon dioxide. The model treats only one cytokine, a single VEGF isoform without consideration of other larger VEGF isoforms, PEDF, PDGF, erythropoietin, angiopoetin-1, angiopoetin-2 or angiopoetin-like 4, all of which likely play at least some role in vascular changes in diabetic retinopathy [117, 140–142]. In this case the level of therapeutic efficacy of the anti-VEGF injectable agents such as ranibizumab or aflibercept supports VEGF being a major [12, 100,143] if not the only important factor, supporting its relevancy and use in the model. This is in addition to all the evidence cited above (see **Support for model’s physiological assumptions**) supporting in choice of VEGF as the factor released from Mueller cells and ultimately responsible for capillary occlusion through elevation of ICAM and resultant leukostasis mediated capillary occlusion.

Movement of VEGF is a concern as there are a number of VEGF isoforms which have different molecular weights and also different structural domains which bind to tissue matrix [143]. Distribution of VEGF may depend on different states of the vitreous gel: attached, detached, syneretic, or absent (post-vitrectomy), as analogously, oxygen levels are dependent on intraocular location prior to, but not post, vitrectomy which presumably allows free advection [144]. We model only intra-retinal levels of VEGF, not vitreal levels, and there is little evidence specifically on these intraretinal levels and likely little relationship between these and the intra-vitreal levels seen late in the disease. It would seem that the current model is most applicable to the situation of non-proliferative disease with an attached intact vitreous resulting in minimal VEGF advection via fluid flow within the posterior chamber of the eye. This sort of elevated advecting VEGF would introduce another source of VEGF influence on local retinal areas not treated in the model.

The details of the complex mechanism of permanent capillary occlusion are unknown but it is likely that multiple intercellular adhesion events occur locally with brief capillary occlusions but also with endothelial cell damage and loss and that over time a local as well as a replacement endothelial cell population from the bone marrow are exhausted [145]. This seems to be another aspect of the impaired physiology of the diabetic. The model assumes a one-time irreversible occlusion event which we interpret as the local coup de grace for that capillary segment following a number of prior endothelial damaging leukocyte adhesion events. In the model occlusion probability was assigned a formula related to local VEGF levels as well as vessel size and flow. This is a sensible approximation to a complicated recurrent local adhesion/occlusion event but has no specific supporting data from the literature.

There are also limitations in the model’s approximation to the capillary anatomy in the macula. The model is three dimensional but the capillary bed treated is only two dimensional in the sense that it is a single lamina thought of as the retinal ganglion cell layer capillary network. It is a single capillary layer but only for about 60 microns from the FAZ. There are currently technological difficulties with resolution of the several capillary layers present further from the FAZ and in this area the model is based only on the inner capillary network imaged by AOSLO. The macular thickness increases over the clivus and thus the amount of tissue dependent on the capillary bed is changing with distance from the FAZ.

A number of clinical signs of diabetic retinopathy were not modelled such as microaneurysms, intra-retinal microvascular abnormalities, cotton wool spots, or dot/blot hemorrhages. These signs can reflect local ischemia and locally elevated VEGF but we feel they are not etiologically related to the topic of progression of capillary occlusions.

The word ‘periphery’ is used loosely in ophthalmology. A model limitation regarding the retinal periphery is that distinct and varying retinal vascular anatomy is seen from the most posterior retinal periphery, that near the arcades (Fig 1), to that anterior to the eye’s equator and especially that most anterior retina near the ora serrata (S8 Fig). We have modelled the extreme periphery (anatomically a single capillary layer) and made statements about ‘peripheral retina’ in general, much of which posterior to the equator has 2 capillary layers.

### Summary

We have implemented a quantitative model of the impact of diabetes on progressive capillary occlusion in retinal capillary networks producing a number of qualitative results very similar to the clinical picture in diabetic retinopathy. A full vascular model from a retinal arteriole to a venule with the linking capillaries is included with oxygen advection, oxygen diffusion, and oxygen consumption. This model requires only the capillary network anatomy, arteriolar and venular pressures, and a number of physical parameters such as a diffusion constant for VEGF and for oxygen. The model produces capillary occlusion on a stochastic basis dependent on local VEGF levels and flow where the local VEGF level is seen physiologically as elevating ICAMs, increasing leukocyte adhesion and ultimately resulting in the capillary occlusion. This results in a local adverse feedback cycle often producing progressive capillary occlusions and enlarging contiguous areas of retinal ischemia. With these simple properties if the model is implemented using a capillary network for a perifoveal area it shows regions of ischemia and macular edema similar to what is seen clinically in the macula including enlargement of the foveal avascular zone. If implemented on a vascular pattern similar to that seen in peripheral retina, model damage proceeds more in line with what is seen clinically in the periphery developing large ischemic areas without edema. This model seems to show that the type and nature of retinal damage depends largely on the pre-existing capillary network morphology, suggesting that it may, after further refinement and validation, have value not only by providing a model through which to augment our understanding of diabetic retinopathy but even potentially, after sufficient clinical validation, for prognostic evaluations and timing of treatment in individual patients.

## Supporting Information

S1 Fig (CASE2) Flow velocity pattern following initial capillary occlusion. (A) Flow velocity map captures loss of a flow pathway due to capillary occlusion in week 0. (B) The second capillary spatially close to initial occlusion site became occluded in week 76. (C) A capillary near FAZ became occluded in week 84. (D) More than a quarter of the capillary network was obstructed by week 96. Color and pointing direction of arrows reflect magnitude and orientation of velocities. The redder the color the greater the flow velocity is. The unit for velocity is μm/s. “FAZ” in the figure refers to foveal avascular zone, “A” in red refers to arteriole, and “V” refers to venule.
(TIF)

S2 Fig (CASE2) Oxygen tension pattern following initial capillary occlusion. (A) Oxygen tension map shows a localized hypoxic region near the occluded capillary in week 0. (B) Hypoxic area of cells broadened to enclose second occlusion site in week 76, but it is still restricted and confined spatially to the Arteriole-Venule district. (C) Area of hypoxia grew in week 84. (D) Large area of hypoxia was observed in week 96, but the propagation of occlusion was still bounded within one Arteriole-Venule sector. Color reflects magnitude of oxygen tension. The redder the color the higher the oxygen tension is. The unit for oxygen tension is mmHg. “FAZ” in the figure refers to foveal avascular zone, “A” in red refers to arteriole, and “V” refers to venule.
(TIF)

S3 Fig (CASE2) VEGF level pattern following initial capillary occlusion. (A) VEGF level map shows localized synthesis of VEGF by Mueller cells in response to hypoxia in week 0. (B)—(D) Increasing amount of Mueller cells actively produced VEGF in weeks 72, 84 and 96, where pattern of regions with high VEGF similar to that of area with low oxygen tension. Color reflects magnitude of VEGF level. The redder the color the higher the VEGF level is. VEGF level has arbitrary unit. “FAZ” in the figure refers to foveal avascular zone, “A” in red refers to arteriole, and “V” refers to venule.
(TIF)

S4 Fig (CASE2) Retinal thickness over time. Thickness of the retinal layer is represented by a color map at the end of year 0.5 (A), year 1 (B), year 2 (C) and year 3 (D). The bluer the color, the thicker a local area is. Fluid was formed between year 1 and year 2 near three of the four leaky sites, which were situated within the area of occlusion. A fourth site didn’t start leaking fluid until year 2, but the fluid accumulated eventually to a large volume in year 3. The flow network is overlaid upon the color map to present effective flow paths at the time point of observation. Color bar only represents the thickness of tissue and not the flow velocities. “FAZ” in the figure refers to foveal avascular zone, “A” in red refers to arteriole, and “V” refers to venule.
(TIF)

S5 Fig (CASE2) Quantitative development of model properties with time. (A) System oxygen tension exhibited a rapid decrease between week 76 and week 96. (B) Hypoxic fraction of Mueller cells was observed to grow rapidly within the same time period and eventually about 20% of cells were hypoxic. (C) Total volume inflow rate rose about 15%. (D) Average minimal cell-to-vessel distance maintained an increasing trend to reach more than 1.5 times the Mueller cell diameter, which qualitatively reproduced the temporal pattern observed for hypoxic fraction of Mueller cells.
(TIF)

S6 Fig (CASE2) Cellular oxygen distribution over time. The fraction of cells in each 4 mmHg oxygen bin is shown for the normal condition and 3 additional times. The pattern in CASE2 is quite similar to the CASE 1. The distribution of oxygen tension within all cells exhibited an essentially unimodal shape under the normal condition (normal-red bars) where most cells had oxygen tensions of 10 to 25 mmHg, a small portion of cells located near vessels had higher levels ranging from 32 to 40 mmHg and no cells had an oxygen tension less than 4 mmHg O_2_. Capillary occlusions induced by VEGF gradually altered the distribution (week 0-blue bars, week 76-yellow bars, week 96-cyan bars). An increasing number of cells became hypoxic. The broad peak of cells at moderate levels of oxygen decreased and broadened with more cells both at lower oxygen levels with each successive interval and more cells from about 25–30 mmHg in each successive interval. The cell oxygenation distribution gradually morphs from a unimodal distribution to a bimodal oxygenation distribution. Inset figure shows a comparison between normal condition and week 96 using a line-connecting-dot presentation, with finer oxygen tension spacing between two consecutive data points. The inset figure also strikingly shows the transition from unimodal to bimodal distribution as well as peak decreasing and broadening pattern at moderate levels.
(TIF)

S7 Fig (Hexagonal Network) Structure of artificial hexagonal capillary network. (A) Capillary network determined from ASOLO image for CASE1 and CASE2 simulations. (B) Hexagonal capillary network with selectively reduced edges has edge size of 65*μm* for each hexagon. This size seems to reflect critical vascular spacing that results in similar extent of progression of capillary occlusions on the hexagonal network to that observed in CASE 1 and CASE 2. Larger hexagon sizes result in cells with inappropriately low oxygen tension (hypoxia) under the normal pre-occlusion condition. By contrast, smaller hexagon size results in little to no hypoxia following capillary occlusions and so does not propagate, but would also be of greater density than necessary for tissue requirements. Size of hexagon near 65*μm* seems to be a critical value that affects proper patterning and irrigation of the capillary network.
(TIF)

S8 Fig (peripheral retinal network) Structure of peripheral retinal capillary network. (A) A bit map of 3 sectors between peripheral arteries and veins after Spitznas is shown [103]. The ora serrata is superior and the macula is inferior. This capillary network is unlike those posterior to the equator with large thick-walled capillaries (10 micron lumens) of simple structure (termed ladder capillaries by Spitznas) connecting the arterioles and venules. Superior in the image are arterio-venous shunt vessels with 18 micron lumens (the only arterio-venous shunt vessels in the retina), and low arterio-venous pressure gradients. The A and V along the bottom indicate arterioles and venules. (B) The schematic of the retinal vasculature in the peripheral retina based on Spitznas is shown. This shows a peripheral ‘ladder’ capillary model with connecting shunts between each arteriole and venule. The shunt vessels are 18 microns lumen diameter and the capillaries are about 10 microns.
(TIF)

S9 Fig (Peripheral retinal network) Flow pattern following initial capillary occlusion. (A) Under normal initial conditions, arterioles and venules carried higher blood flows than the ‘rung’ like capillaries did. Letter ‘A’ in red refers to the Arterial end and letter ‘V’ in blue to the Venous end. (B) One of the capillaries was occluded in week 0. (C) The ‘ladder’ like AV sector lost most of the capillary blood flow pathways in week 44, visualized as a large opening in the flow velocity map. (D) Four capillaries out of five became occluded. Color and pointing direction of arrows reflect magnitudes and orientations of velocities respectively, where the redder the color the greater the flow velocity. The unit for velocity is μm/s.
(TIF)

S10 Fig (Peripheral retinal network) Oxygen tension following initial capillary occlusion. (A) Under normal conditions, cells are properly oxygenated. Letter ‘A’ in red refers to the arterial end and letter ‘V’ in blue to the venous end. (B) A localized hypoxic region emerged near the occluded capillary within one arteriole-venule (A-V) sector in week 0. (C) Hypoxic area widened to spread bi-directionally in parallel to arteriole and venule in week 44, but is still confined spatially within the A-V sector. (D) An even greater area of hypoxia, though still confined sector-wise, was observed in week 116. Color reflects magnitude of oxygen tension, where the redder the color the higher the oxygen tension. The unit for oxygen tension is mmHg.
(TIF)

S11 Fig (Peripheral retinal network) VEGF level following initial capillary occlusion. (A) Under normal condition, VEGF maintained in a physiological diabetic baseline level. Letter ‘A’ in red refers to the Arterial end and letter ‘V’ in blue to the Venous end. (B) VEGF level map shows localized synthesis of VEGF by Mueller cells in response to hypoxia in week 0. (C)—(D) Increasing amount of Mueller cells actively produced VEGF in week 44 and 116, despite high levels of VEGF occlusions were unable to cross the border of the AV sector illustrating the effect of oxygenated zones around vessels in prevention of propagation of occlusion. Color reflects magnitude of VEGF level, where the redder the color the higher the VEGF level. VEGF level has arbitrary units.
(TIF)

S12 Fig (Peripheral retinal network) Retinal thickness over time. Thickness of the retina is represented by a color map at the end of year 0.5 (A), year 1 (B), year 2 (C) and year 3 (D). The bluer the color, the thicker a local area is. Very little fluid accumulation was found in the peripheral simulation, as compared to other retinal simulations. The flow network is overlaid upon the color map to present effective flow paths at the time point of observation. Color bar only represents the thickness of tissue but not the flow velocities. “A” in red refers to the arteriole, and “V” refers to the venule.
(TIF)

S13 Fig (Peripheral retinal network) Quantitative development of model properties with time. (A) System oxygen tension exhibited a decreasing trend. (B) Hypoxic fraction of Mueller cells was observed to grow rapidly within the same time period, and eventually more than 15% of cells suffered from poor oxygen supply. (C) Total volume inflow rate maintained a decreasing trend. (D) Average minimal cell-to-vessel distance kept increasing to reach about 1.5 times the size of a Mueller cell.
(TIF)

S14 Fig (Peripheral retinal network) Cellular oxygen distribution over time. The fraction of cells in each 4 mmHg oxygen bin is shown for the normal condition and 3 additional times. The distribution of oxygen tension within all cells exhibited an essentially unimodal shape under the normal condition (normal-red bars) where most cells had oxygen tensions of 10 to 20 mmHg and no cells had an oxygen tension less than 4 mmHg O_2_. Capillary occlusions induced by VEGF gradually altered the distribution (week 0-blue bars, week 44-yellow bars, week 116-cyan bars). An increasing number of cells turned hypoxic. The initial broad peak of cells at moderate levels of oxygen decreased and broadened with more cells at lower oxygen levels with each successive interval. The cell oxygenation distribution gradually morphs from a unimodal distribution to a bimodal oxygenation distribution. One interesting feature of the peripheral network is that except for the hypoxic regime, the oxygen distribution shows much smaller difference than CASE1 and CASE2. One possible explanation is that in the peripheral network simulation, flow velocities in the patent capillary network barely change with occlusions which maintain normal quality of oxygen irrigation and accordingly oxygen tension of cells nearby remains largely unchanged (compare S10A Fig and S10D Fig left half network).
(TIF)

S15 Fig Boundary conditions for CASE1 and CASE2 simulations. Image shows values of blood pressures and oxygen tensions at all boundary nodes. Capillaries extending to outside the region of interest are assigned intermediate blood pressure values between arterial and venous pressures. Boundary nodes that represent inlets are assigned smaller oxygen tension values than arterial oxygen tension.
(TIF)

S16 Fig Parameter influence on capillary network patency index and retinal thickness change in CASE1 simulation. Variations of six parameters one-at-a-time (listed vertically beside figure) at four widely varying values (listed horizontally below figure) are run on replicate simulations. Each colored block represents the average result of 28 simulations with a certain-value variation of a certain parameter from reference parameter set as in CASE1. The CASE1 parameter set used in the modelling in this paper is denoted as “× 1” in the figure. (A) Mean patency index is calculated as percentage of patent (unoccluded) capillaries at the end of each simulation, averaged for 28 simulations with same parameter set. Redder color stands for higher mean patency index, while bluer stands for lower mean patency index. (B) Mean thickness change is calculated as percentage change in retinal thickness from week 0 to the end of the simulation, averaged for 28 simulations with same parameter set. Darker color stands for larger change in thickness, while lighter color for smaller changes.
(TIF)

S1 Protocol Python source code for the simulations. (ZIP)

S1 Table Properties and behaviors of model objects. (DOCX)

S2 Table State transition of model objects. (DOCX)

S3 Table Process and dynamics of field flux. (DOCX)

S4 Table Model Parameters. (DOCX)

S1 Text Documentation of detailed description of the model. (DOCX)

S2 Text Macular simulation with a different initial capillary occlusion site CASE 2. (DOCX)

S3 Text Simulation in a patterned hexagonal capillary network. (DOCX)

S4 Text Simulation of peripheral capillary network schematic. (DOCX)
