# Supplementary figures and images for: Progression of Diabetic Capillary Occlusion: A Model
Source: PLoS Comput Biol. 2016 Jun 14;12(6):e1004932. doi: 10.1371/journal.pcbi.1004932 (PMC4907516; doi:10.1371/journal.pcbi.1004932)

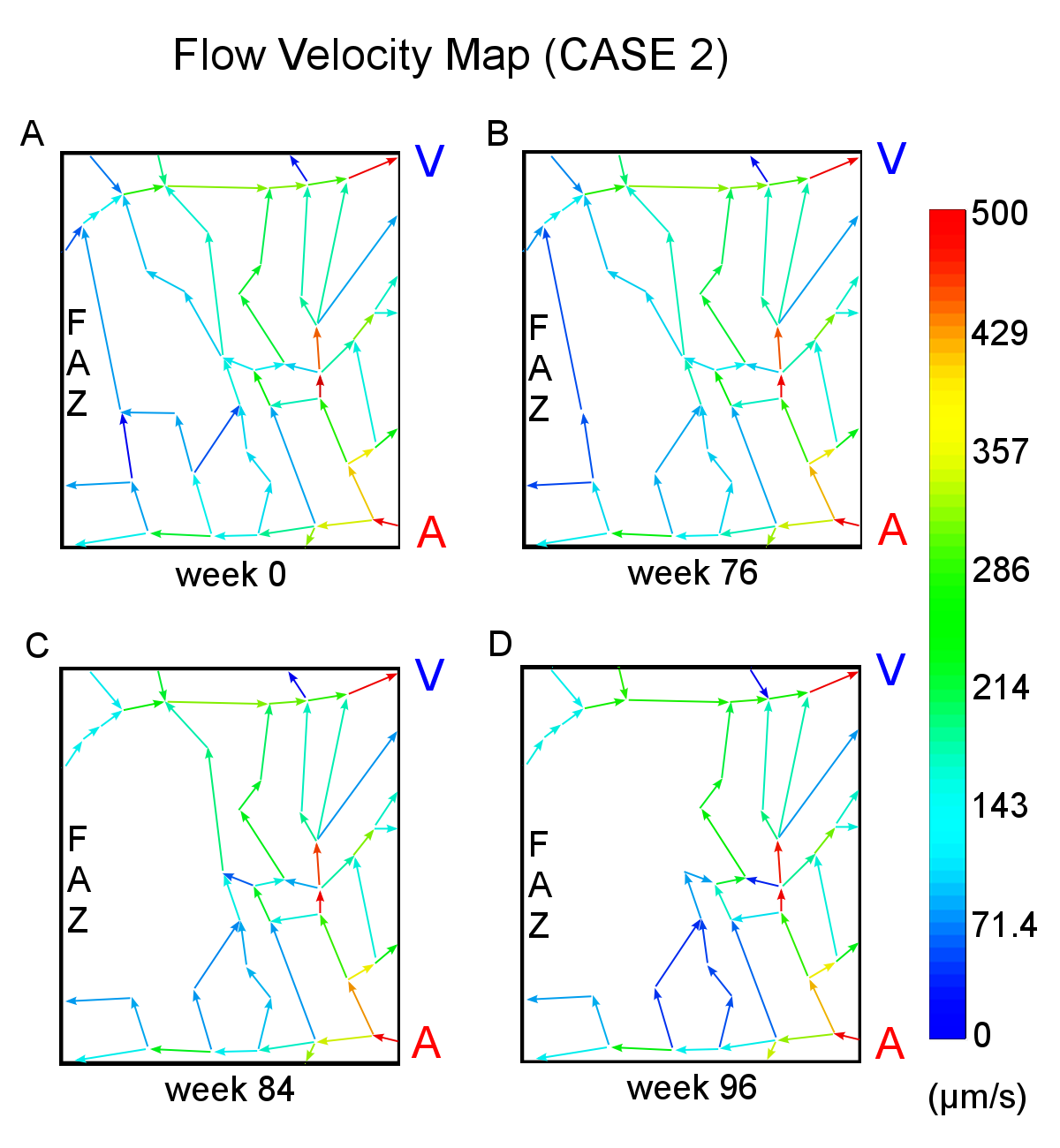

Supplement: S1 Fig — (A) Flow velocity map captures loss of a flow pathway due to capillary occlusion in week 0. (B) The second capillary spatially close to initial occlusion site became occluded in week 76. (C) A capillary near FAZ became occluded in week 84. (D) More than a quarter of the capillary network was obstructed by week 96. Color and pointing direction of arrows reflect magnitude and orientation of velocities. The redder the color the greater the flow velocity is. The unit for velocity is μm/s. “FAZ” in the figure refers to foveal avascular zone, “A” in red refers to arteriole, and “V” refers to venule. (TIF) [file pcbi.1004932.s001.tif]

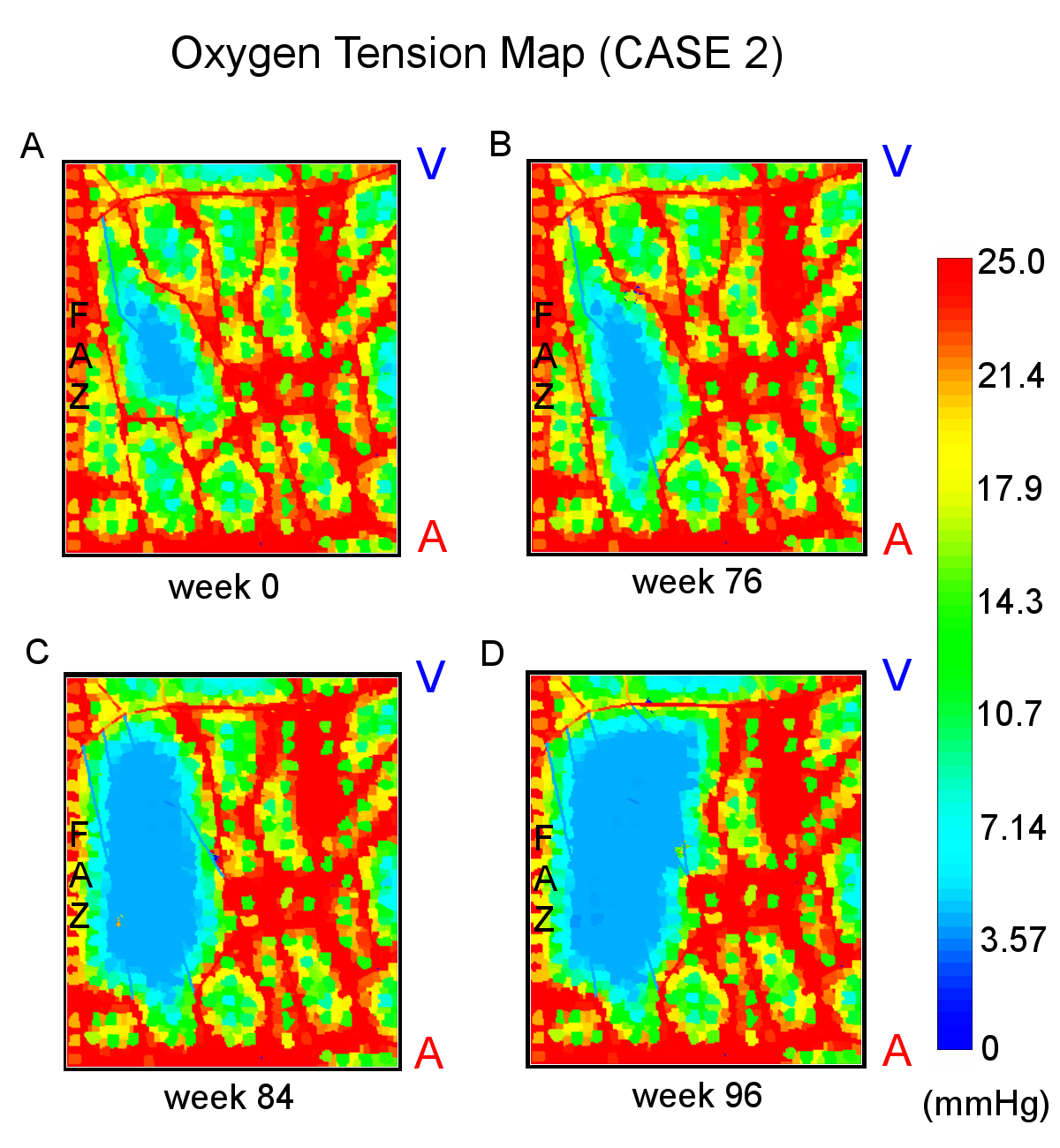

Supplement: S2 Fig — (A) Oxygen tension map shows a localized hypoxic region near the occluded capillary in week 0. (B) Hypoxic area of cells broadened to enclose second occlusion site in week 76, but it is still restricted and confined spatially to the Arteriole-Venule district. (C) Area of hypoxia grew in week 84. (D) Large area of hypoxia was observed in week 96, but the propagation of occlusion was still bounded within one Arteriole-Venule sector. Color reflects magnitude of oxygen tension. The redder the color the higher the oxygen tension is. The unit for oxygen tension is mmHg. “FAZ” in the figure refers to foveal avascular zone, “A” in red refers to arteriole, and “V” refers to venule. (TIF) [file pcbi.1004932.s002.tif]

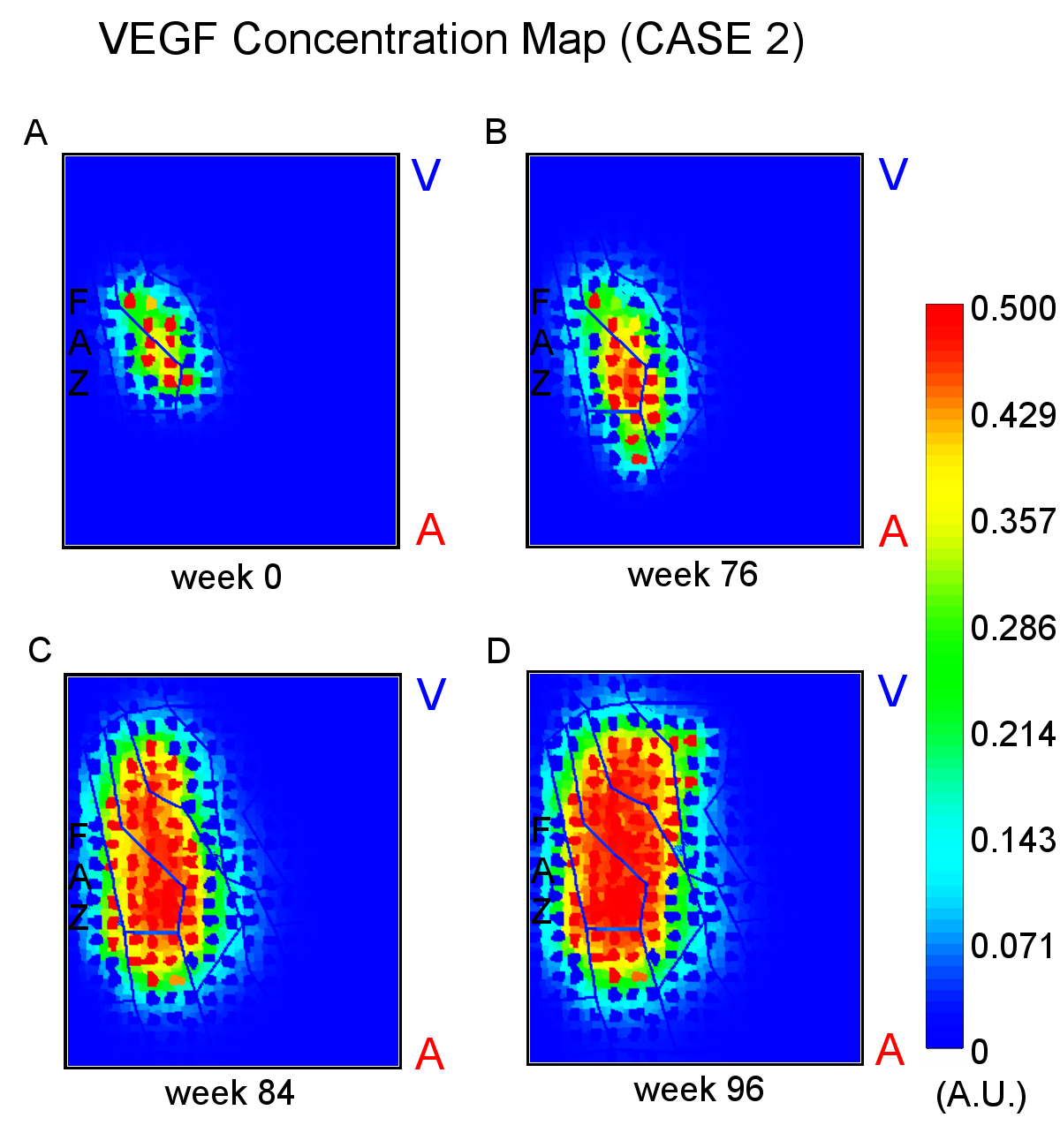

Supplement: S3 Fig — (A) VEGF level map shows localized synthesis of VEGF by Mueller cells in response to hypoxia in week 0. (B)—(D) Increasing amount of Mueller cells actively produced VEGF in weeks 72, 84 and 96, where pattern of regions with high VEGF similar to that of area with low oxygen tension. Color reflects magnitude of VEGF level. The redder the color the higher the VEGF level is. VEGF level has arbitrary unit. “FAZ” in the figure refers to foveal avascular zone, “A” in red refers to arteriole, and “V” refers to venule. (TIF) [file pcbi.1004932.s003.tif]

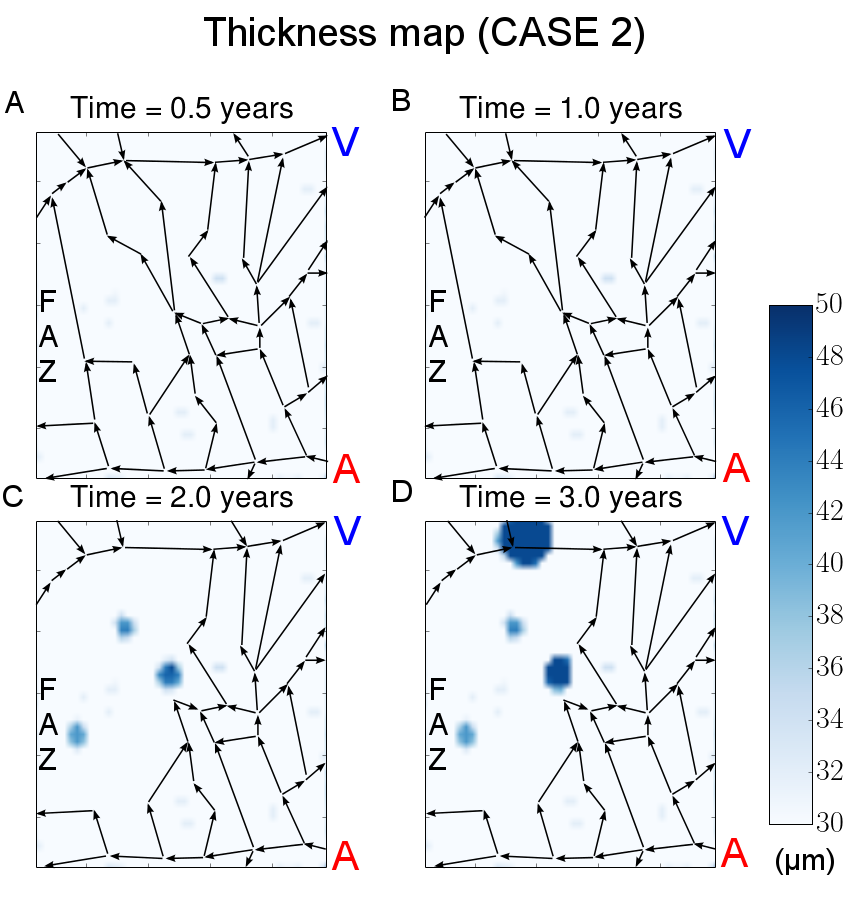

Supplement: S4 Fig — Thickness of the retinal layer is represented by a color map at the end of year 0.5 (A), year 1 (B), year 2 (C) and year 3 (D). The bluer the color, the thicker a local area is. Fluid was formed between year 1 and year 2 near three of the four leaky sites, which were situated within the area of occlusion. A fourth site didn’t start leaking fluid until year 2, but the fluid accumulated eventually to a large volume in year 3. The flow network is overlaid upon the color map to present effective flow paths at the time point of observation. Color bar only represents the thickness of tissue and not the flow velocities. “FAZ” in the figure refers to foveal avascular zone, “A” in red refers to arteriole, and “V” refers to venule. (TIF) [file pcbi.1004932.s004.tif]

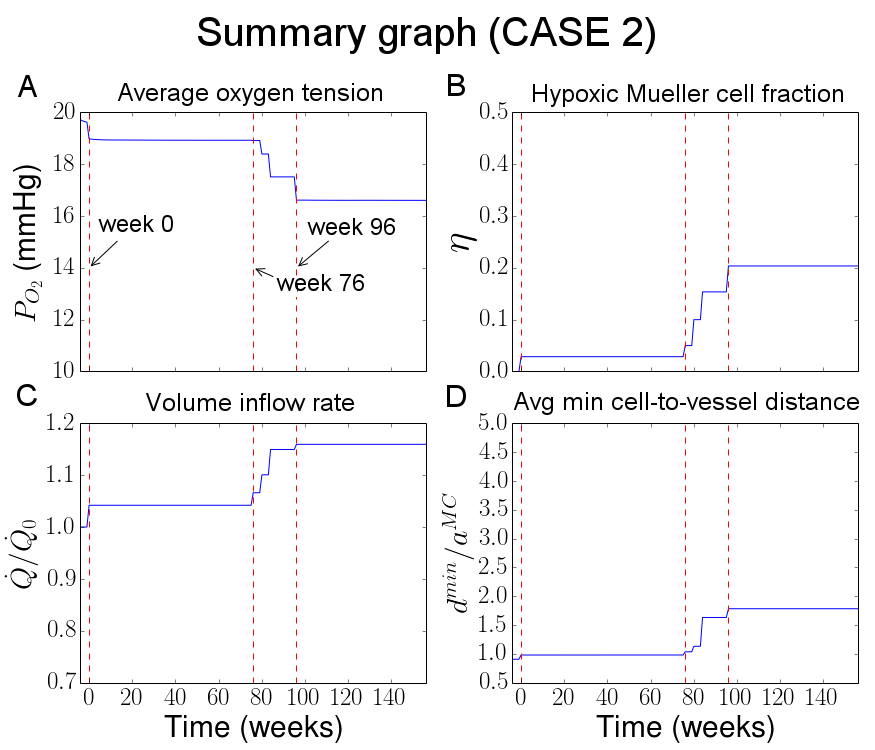

Supplement: S5 Fig — (A) System oxygen tension exhibited a rapid decrease between week 76 and week 96. (B) Hypoxic fraction of Mueller cells was observed to grow rapidly within the same time period and eventually about 20% of cells were hypoxic. (C) Total volume inflow rate rose about 15%. (D) Average minimal cell-to-vessel distance maintained an increasing trend to reach more than 1.5 times the Mueller cell diameter, which qualitatively reproduced the temporal pattern observed for hypoxic fraction of Mueller cells. (TIF) [file pcbi.1004932.s005.tif]

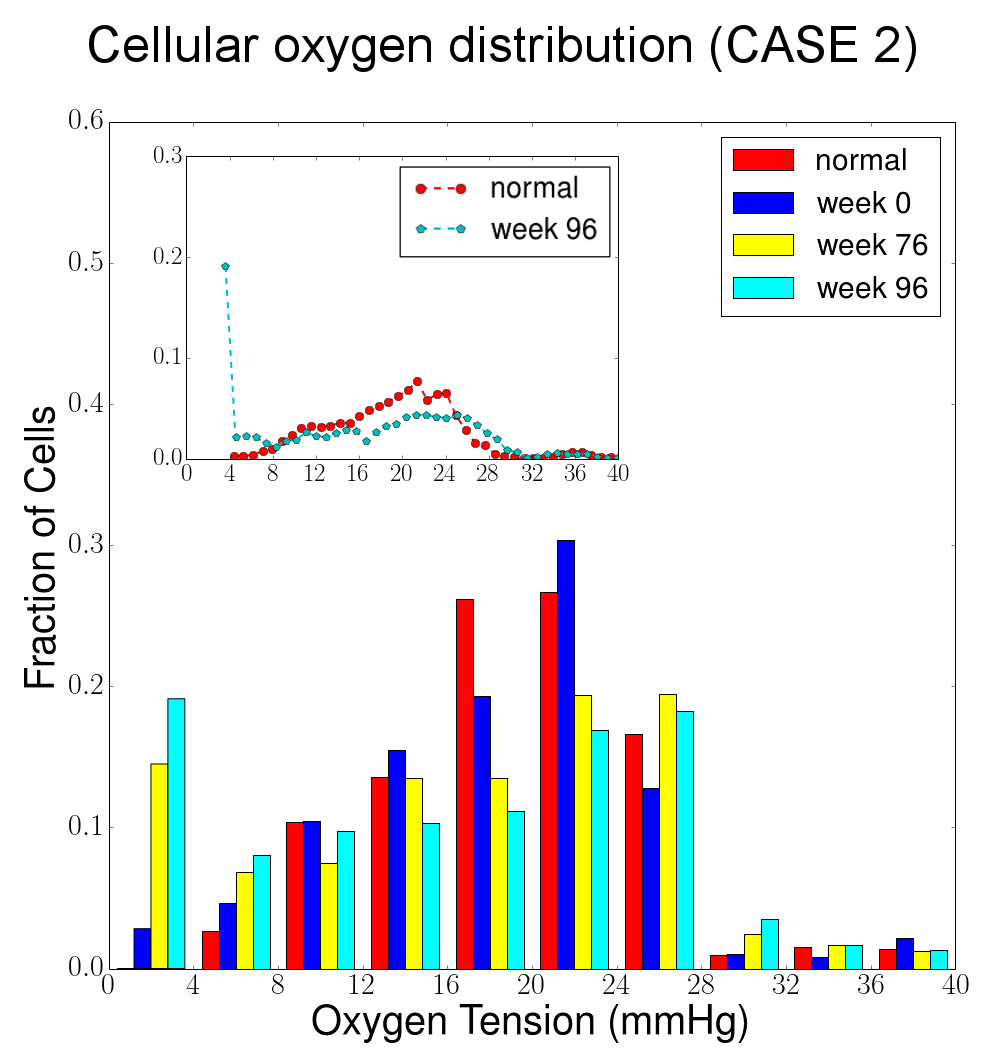

Supplement: S6 Fig — The fraction of cells in each 4 mmHg oxygen bin is shown for the normal condition and 3 additional times. The pattern in CASE2 is quite similar to the CASE 1. The distribution of oxygen tension within all cells exhibited an essentially unimodal shape under the normal condition (normal-red bars) where most cells had oxygen tensions of 10 to 25 mmHg, a small portion of cells located near vessels had higher levels ranging from 32 to 40 mmHg and no cells had an oxygen tension less than 4 mmHg O2. Capillary occlusions induced by VEGF gradually altered the distribution (week 0-blue bars, week 76-yellow bars, week 96-cyan bars). An increasing number of cells became hypoxic. The broad peak of cells at moderate levels of oxygen decreased and broadened with more cells both at lower oxygen levels with each successive interval and more cells from about 25–30 mmHg in each successive interval. The cell oxygenation distribution gradually morphs from a unimodal distribution to a bimodal oxygenation distribution. Inset figure shows a comparison between normal condition and week 96 using a line-connecting-dot presentation, with finer oxygen tension spacing between two consecutive data points. The inset figure also strikingly shows the transition from unimodal to bimodal distribution as well as peak decreasing and broadening pattern at moderate levels. (TIF) [file pcbi.1004932.s006.tif]

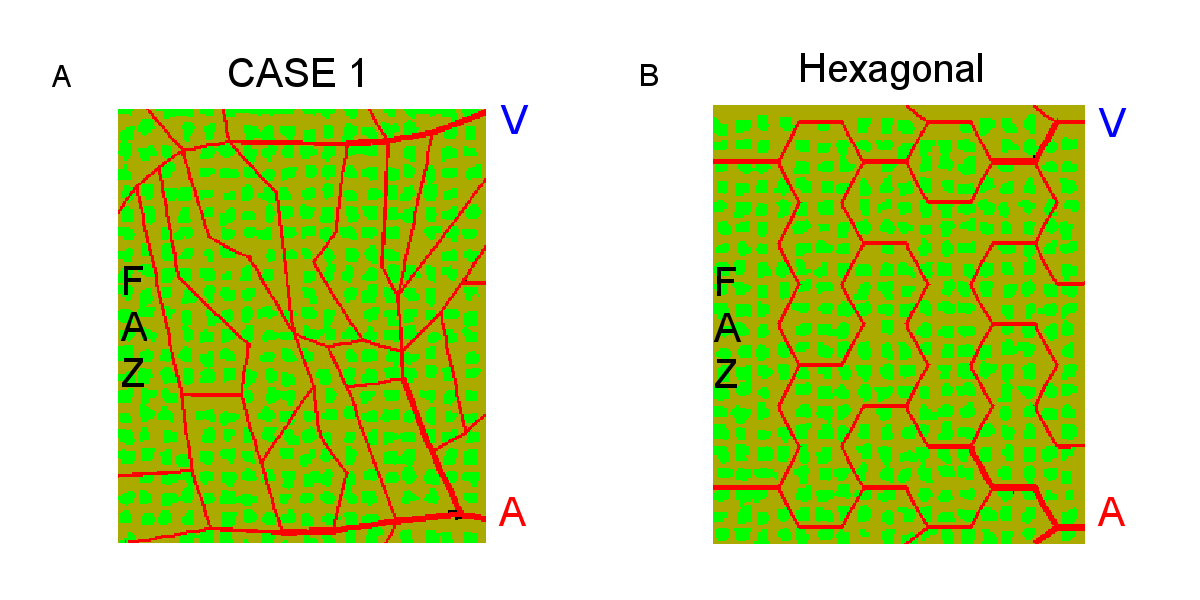

Supplement: S7 Fig — (A) Capillary network determined from ASOLO image for CASE1 and CASE2 simulations. (B) Hexagonal capillary network with selectively reduced edges has edge size of 65μm for each hexagon. This size seems to reflect critical vascular spacing that results in similar extent of progression of capillary occlusions on the hexagonal network to that observed in CASE 1 and CASE 2. Larger hexagon sizes result in cells with inappropriately low oxygen tension (hypoxia) under the normal pre-occlusion condition. By contrast, smaller hexagon size results in little to no hypoxia following capillary occlusions and so does not propagate, but would also be of greater density than necessary for tissue requirements. Size of hexagon near 65μm seems to be a critical value that affects proper patterning and irrigation of the capillary network. (TIF) [file pcbi.1004932.s007.tif]

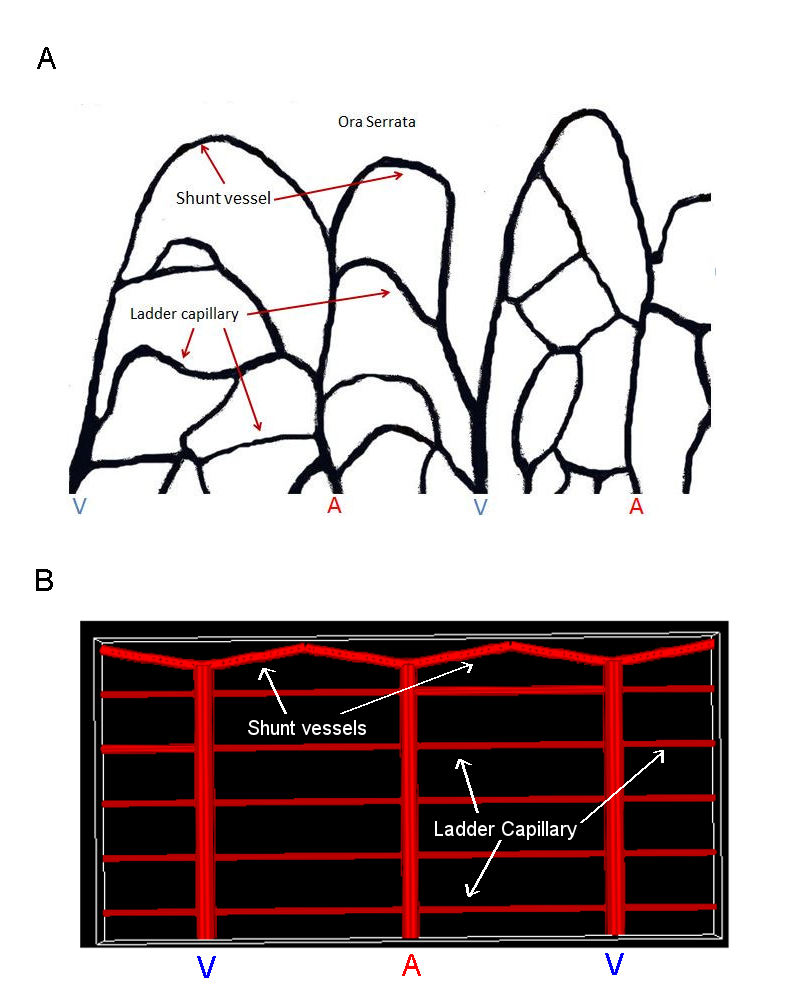

Supplement: S8 Fig — (A) A bit map of 3 sectors between peripheral arteries and veins after Spitznas is shown [103]. The ora serrata is superior and the macula is inferior. This capillary network is unlike those posterior to the equator with large thick-walled capillaries (10 micron lumens) of simple structure (termed ladder capillaries by Spitznas) connecting the arterioles and venules. Superior in the image are arterio-venous shunt vessels with 18 micron lumens (the only arterio-venous shunt vessels in the retina), and low arterio-venous pressure gradients. The A and V along the bottom indicate arterioles and venules. (B) The schematic of the retinal vasculature in the peripheral retina based on Spitznas is shown. This shows a peripheral ‘ladder’ capillary model with connecting shunts between each arteriole and venule. The shunt vessels are 18 microns lumen diameter and the capillaries are about 10 microns. (TIF) [file pcbi.1004932.s008.tif]

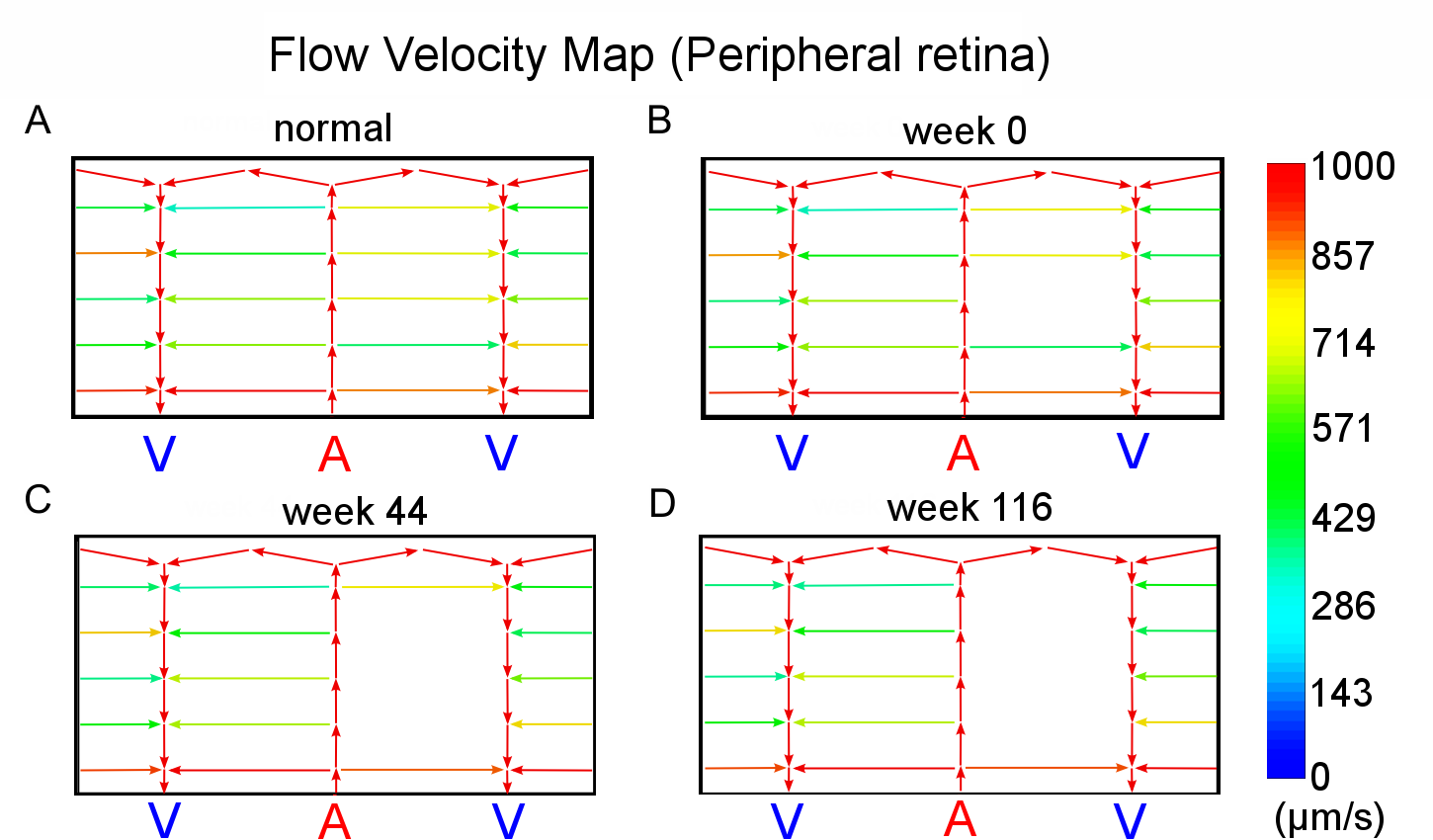

Supplement: S9 Fig — (A) Under normal initial conditions, arterioles and venules carried higher blood flows than the ‘rung’ like capillaries did. Letter ‘A’ in red refers to the Arterial end and letter ‘V’ in blue to the Venous end. (B) One of the capillaries was occluded in week 0. (C) The ‘ladder’ like AV sector lost most of the capillary blood flow pathways in week 44, visualized as a large opening in the flow velocity map. (D) Four capillaries out of five became occluded. Color and pointing direction of arrows reflect magnitudes and orientations of velocities respectively, where the redder the color the greater the flow velocity. The unit for velocity is μm/s. (TIF) [file pcbi.1004932.s009.tif]

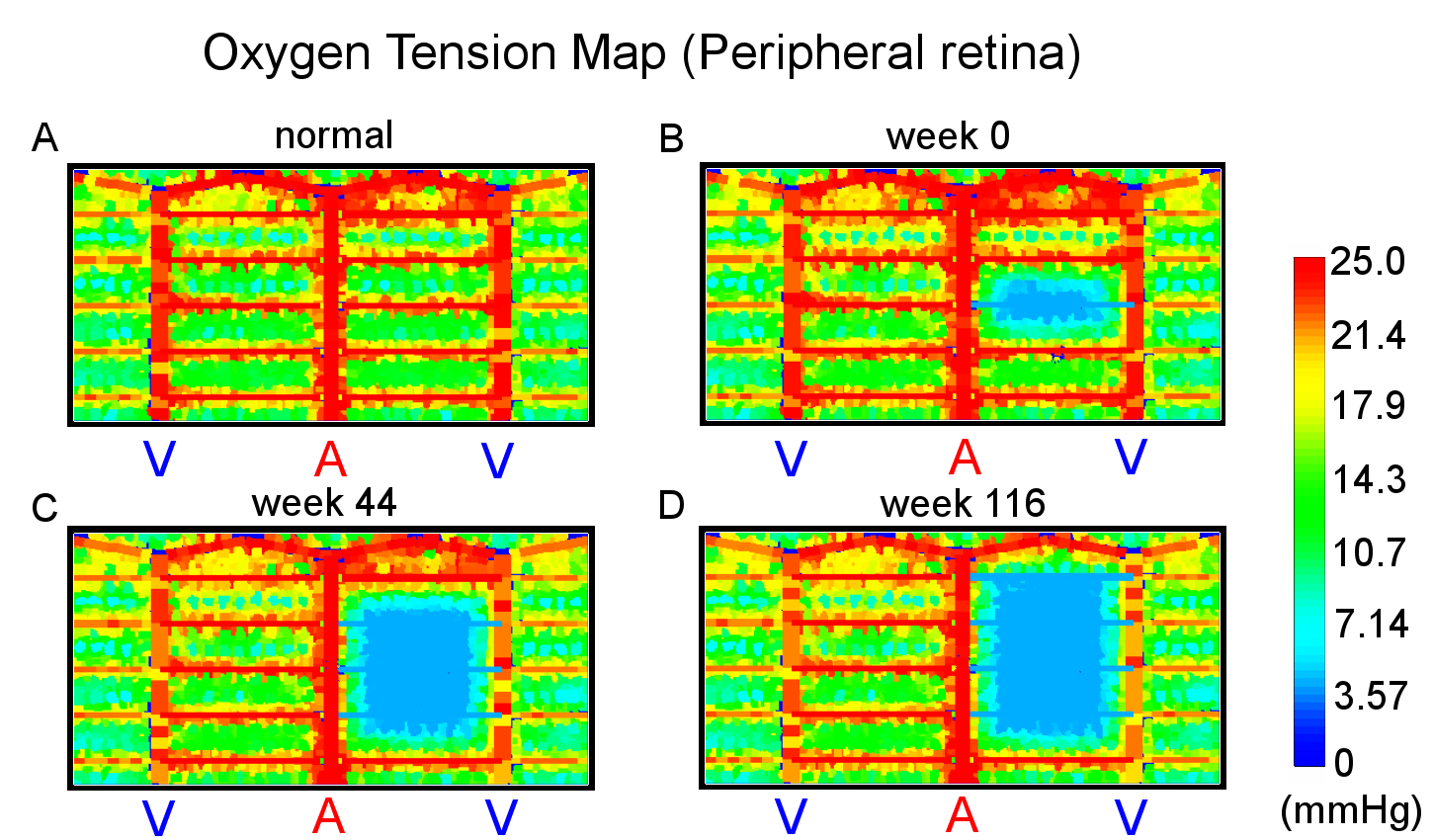

Supplement: S10 Fig — (A) Under normal conditions, cells are properly oxygenated. Letter ‘A’ in red refers to the arterial end and letter ‘V’ in blue to the venous end. (B) A localized hypoxic region emerged near the occluded capillary within one arteriole-venule (A-V) sector in week 0. (C) Hypoxic area widened to spread bi-directionally in parallel to arteriole and venule in week 44, but is still confined spatially within the A-V sector. (D) An even greater area of hypoxia, though still confined sector-wise, was observed in week 116. Color reflects magnitude of oxygen tension, where the redder the color the higher the oxygen tension. The unit for oxygen tension is mmHg. (TIF) [file pcbi.1004932.s010.tif]

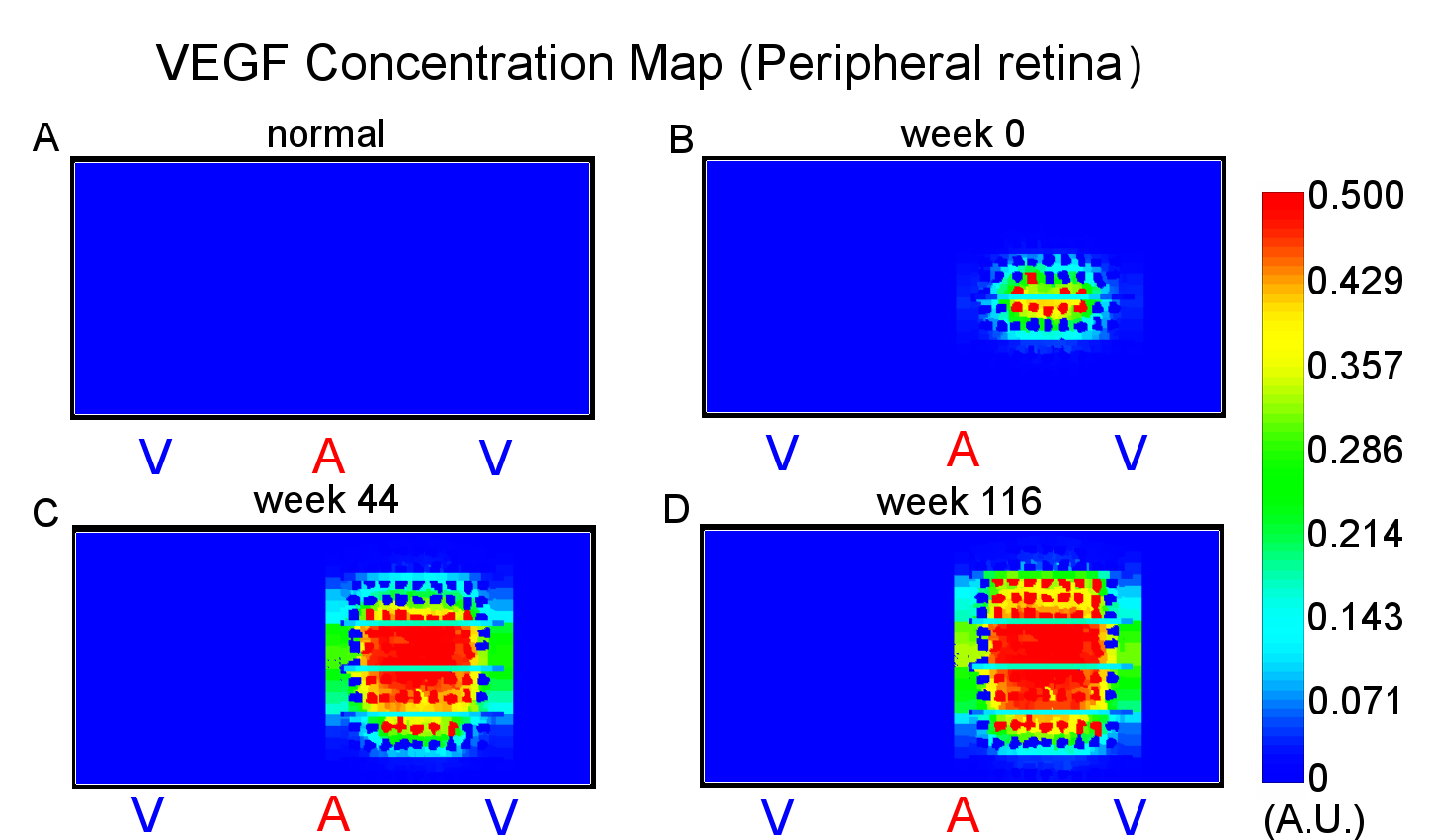

Supplement: S11 Fig — (A) Under normal condition, VEGF maintained in a physiological diabetic baseline level. Letter ‘A’ in red refers to the Arterial end and letter ‘V’ in blue to the Venous end. (B) VEGF level map shows localized synthesis of VEGF by Mueller cells in response to hypoxia in week 0. (C)—(D) Increasing amount of Mueller cells actively produced VEGF in week 44 and 116, despite high levels of VEGF occlusions were unable to cross the border of the AV sector illustrating the effect of oxygenated zones around vessels in prevention of propagation of occlusion. Color reflects magnitude of VEGF level, where the redder the color the higher the VEGF level. VEGF level has arbitrary units. (TIF) [file pcbi.1004932.s011.tif]

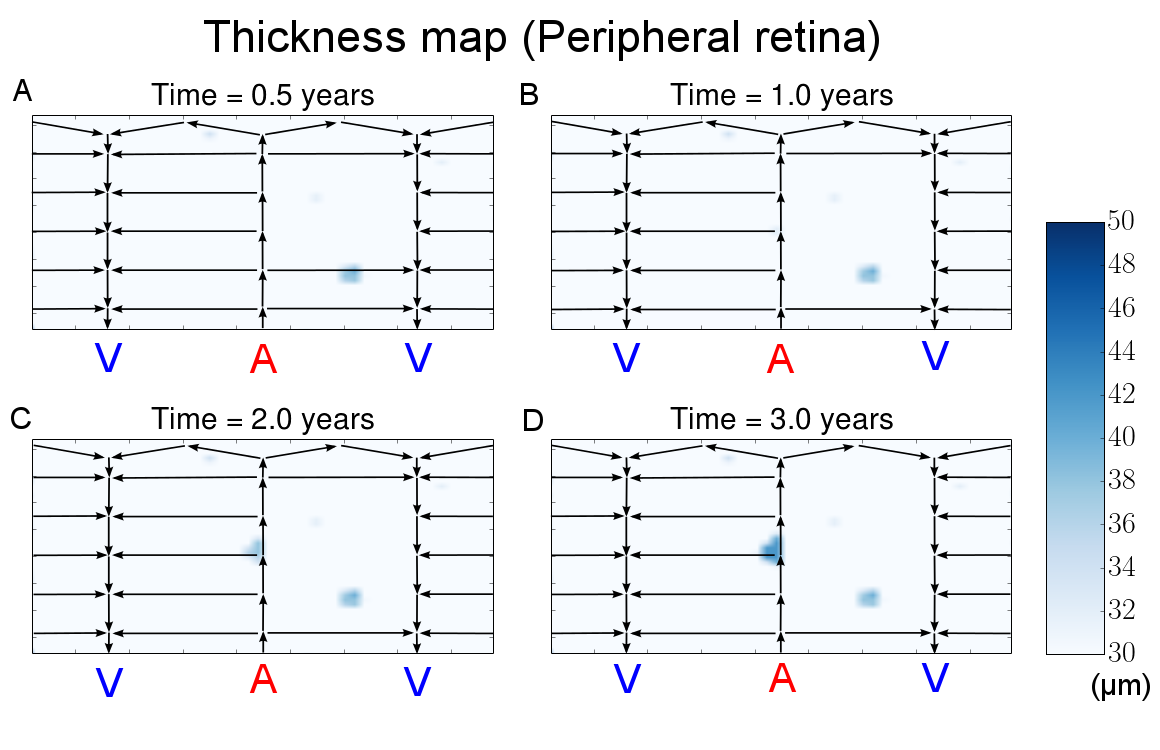

Supplement: S12 Fig — Thickness of the retina is represented by a color map at the end of year 0.5 (A), year 1 (B), year 2 (C) and year 3 (D). The bluer the color, the thicker a local area is. Very little fluid accumulation was found in the peripheral simulation, as compared to other retinal simulations. The flow network is overlaid upon the color map to present effective flow paths at the time point of observation. Color bar only represents the thickness of tissue but not the flow velocities. “A” in red refers to the arteriole, and “V” refers to the venule. (TIF) [file pcbi.1004932.s012.tif]

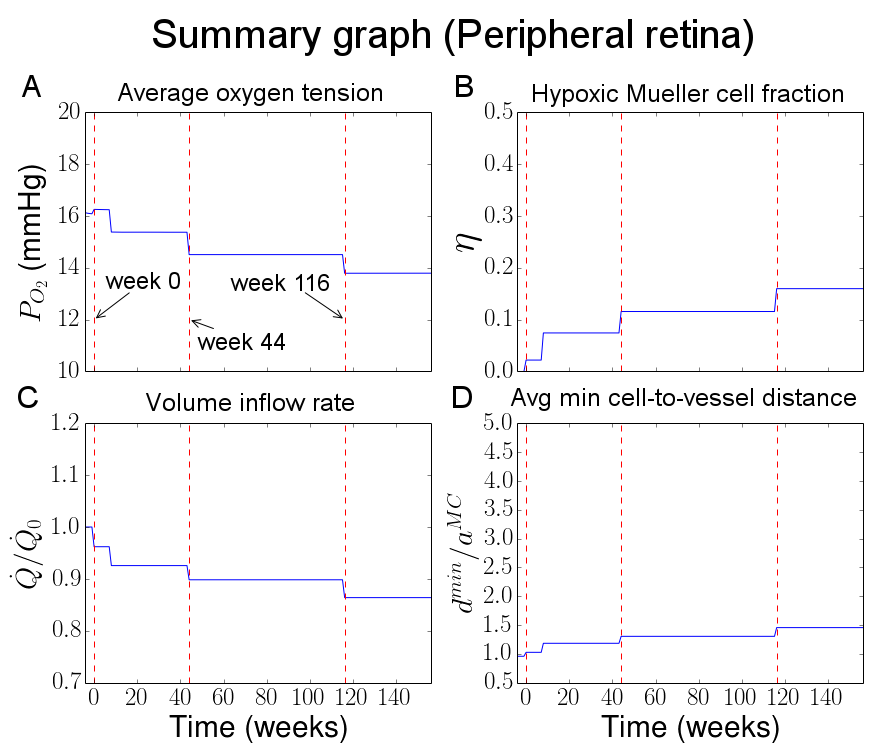

Supplement: S13 Fig — (A) System oxygen tension exhibited a decreasing trend. (B) Hypoxic fraction of Mueller cells was observed to grow rapidly within the same time period, and eventually more than 15% of cells suffered from poor oxygen supply. (C) Total volume inflow rate maintained a decreasing trend. (D) Average minimal cell-to-vessel distance kept increasing to reach about 1.5 times the size of a Mueller cell. (TIF) [file pcbi.1004932.s013.tif]

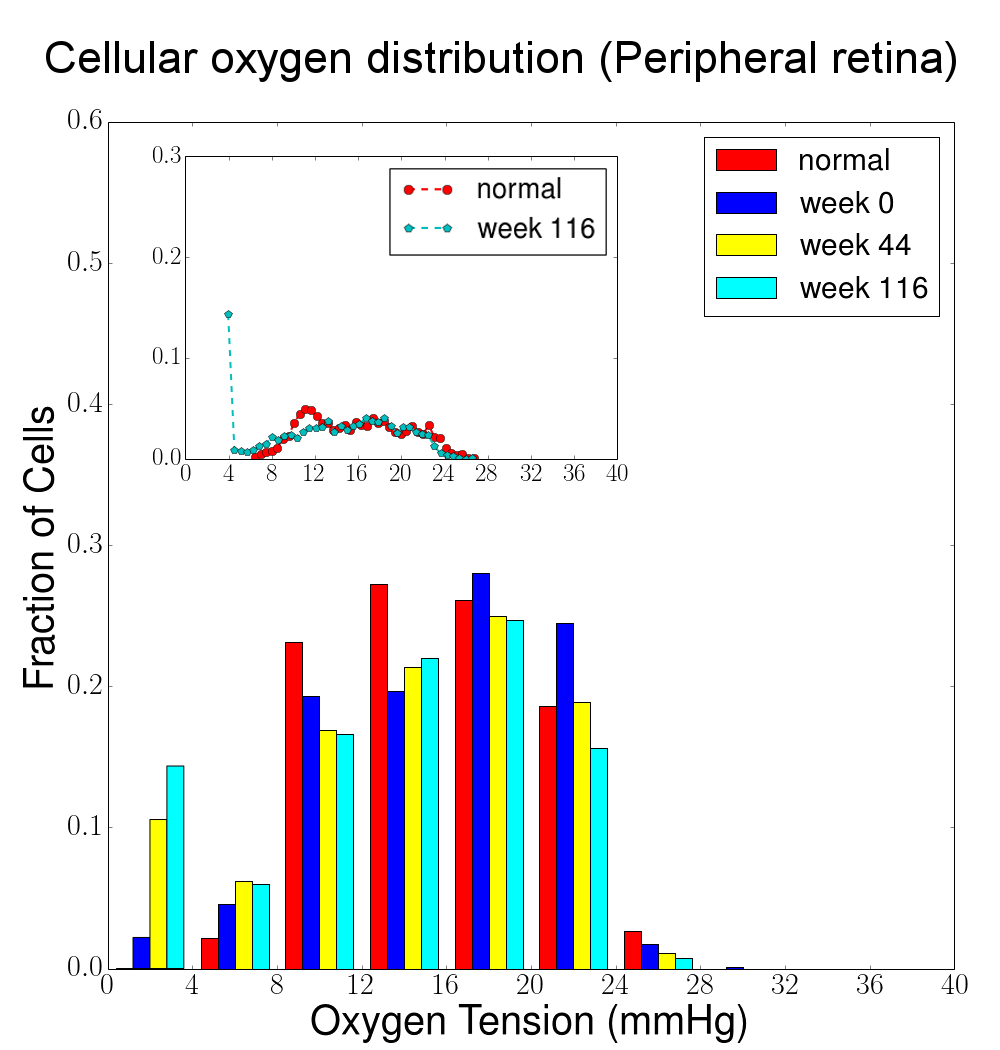

Supplement: S14 Fig — The fraction of cells in each 4 mmHg oxygen bin is shown for the normal condition and 3 additional times. The distribution of oxygen tension within all cells exhibited an essentially unimodal shape under the normal condition (normal-red bars) where most cells had oxygen tensions of 10 to 20 mmHg and no cells had an oxygen tension less than 4 mmHg O2. Capillary occlusions induced by VEGF gradually altered the distribution (week 0-blue bars, week 44-yellow bars, week 116-cyan bars). An increasing number of cells turned hypoxic. The initial broad peak of cells at moderate levels of oxygen decreased and broadened with more cells at lower oxygen levels with each successive interval. The cell oxygenation distribution gradually morphs from a unimodal distribution to a bimodal oxygenation distribution. One interesting feature of the peripheral network is that except for the hypoxic regime, the oxygen distribution shows much smaller difference than CASE1 and CASE2. One possible explanation is that in the peripheral network simulation, flow velocities in the patent capillary network barely change with occlusions which maintain normal quality of oxygen irrigation and accordingly oxygen tension of cells nearby remains largely unchanged (compare S10A Fig and S10D Fig left half network). (TIF) [file pcbi.1004932.s014.tif]

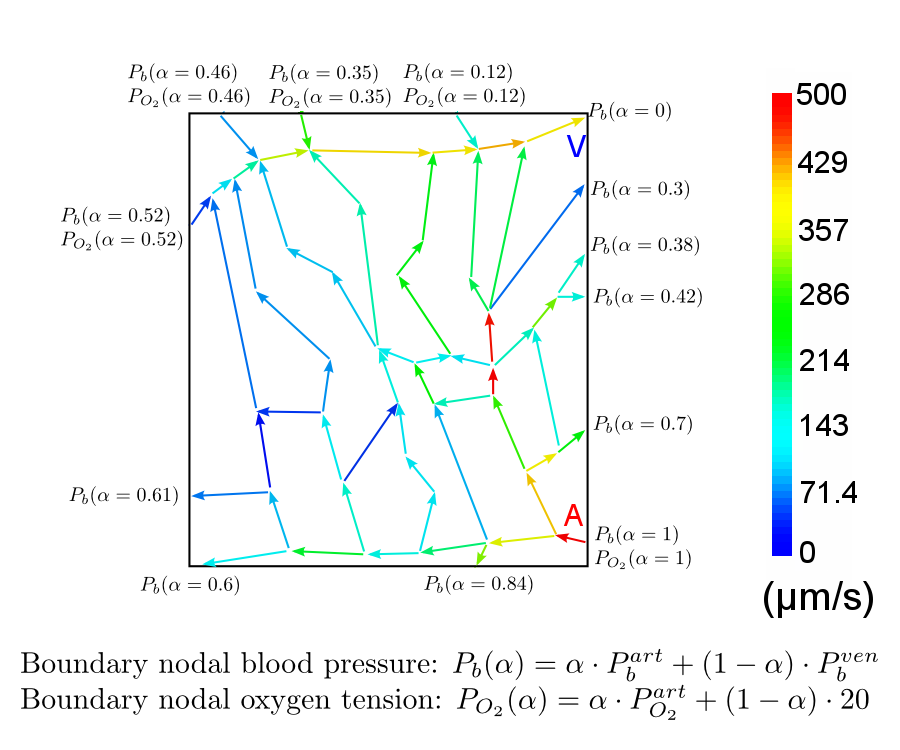

Supplement: S15 Fig — Image shows values of blood pressures and oxygen tensions at all boundary nodes. Capillaries extending to outside the region of interest are assigned intermediate blood pressure values between arterial and venous pressures. Boundary nodes that represent inlets are assigned smaller oxygen tension values than arterial oxygen tension. (TIF) [file pcbi.1004932.s015.tif]

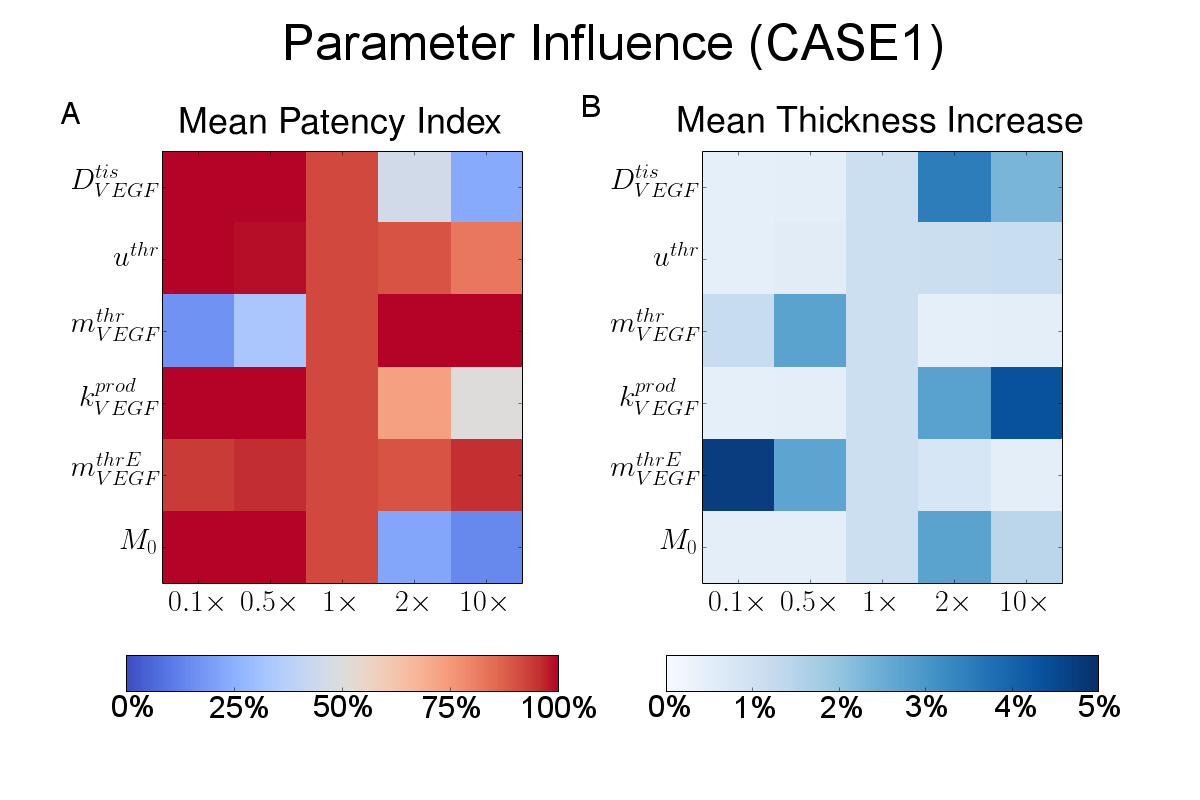

Supplement: S16 Fig — Variations of six parameters one-at-a-time (listed vertically beside figure) at four widely varying values (listed horizontally below figure) are run on replicate simulations. Each colored block represents the average result of 28 simulations with a certain-value variation of a certain parameter from reference parameter set as in CASE1. The CASE1 parameter set used in the modelling in this paper is denoted as “× 1” in the figure. (A) Mean patency index is calculated as percentage of patent (unoccluded) capillaries at the end of each simulation, averaged for 28 simulations with same parameter set. Redder color stands for higher mean patency index, while bluer stands for lower mean patency index. (B) Mean thickness change is calculated as percentage change in retinal thickness from week 0 to the end of the simulation, averaged for 28 simulations with same parameter set. Darker color stands for larger change in thickness, while lighter color for smaller changes. (TIF) [file pcbi.1004932.s016.tif]
