# Supplementary material for: Progression of Diabetic Capillary Occlusion: A Model
Source: PLoS Comput Biol. 2016 Jun 14;12(6):e1004932. doi: 10.1371/journal.pcbi.1004932 (PMC4907516; doi:10.1371/journal.pcbi.1004932)
Supplement: S1 Table — (DOCX) [file pcbi.1004932.s018.docx]

**S1 Table Properties and behaviors of model objects**

| Objects | | Properties | Behaviors |
| --- | --- | --- | --- |
| Generalized Cell | Other |  |  |
| Capillary Block (**CAP**) |  | (1) **CAP** is a structural unit of each capillary segment between two junctions. Each **CAP** represents a composite of endothelium and blood. **CAP** has a cylindrical shape, with diameter given by$d^{cap}$.  (2) Each **CAP** has more than one affiliated ***CB***s. | (1) **CAP** transfers *O_2_* into **MC*,* OT** and **FP** in contact with it.  (2) **CAP** uptakes *VEGF* from **OT** and **FP** in contact. A **CAP** can become leaky or occluded in the presence of high level of *VEGF*, if the conditions are met respectively.  (3) **CAP** destructs *VEGF*. |
| Mueller cell (**MC**) |  | (1) **MC** doesn't have a defined shape initially; each **MC** has a 1-voxel-size CC3D seed to grow from.  (2) **MC** has typical size of$a^{\mathrm{MC}}$. | (1) **MC** transfers and metabolizes *O_2_*. When cellular store of *O_2_* drops below a critical value, **MC** becomes hypoxic.  (2) **MC** produces and releases *VEGF* in an *O_2_* tension-dependent manner.  (3) **MC** transfers and destructs *VEGF*. |
| Other retinal cell (**OT**) |  | (1) **OT** doesn't have a defined shape initially; each **OT** has a 1-voxel-size CC3D seed to grow from.  (2) **OT** has typical size of$a^{\mathrm{OT}}$. | (1) **OT** transfers and metabolizes *O_2_*.  (2) **OT** transfers and destructs *VEGF*. |
| Fluid portion (**FP**) |  | (1 **FP** doesn't have defined shape initially; however, each **FP** has a 1-voxel-size CC3D seed to grow from beside a leaky **CAP**.  (2) **FP** has typical volume of${vol}^{\mathrm{FP}}$. | (1) **FP** transfers *O_2_*.  (2) **FP** transfers and destructs *VEGF*.  (3) **FP** is created close to a leaky **CAP**  (4) **FP** in contact with bottom surface of the system shrinks with time, representing RPE’s capability of pumping away leaked fluid. |
|  | Conveyor- belt Block (***CB***) | (1) ***CB*** is a functional unit for oxygen advection, which conceptually represented block of blood containing *O_2_* within a host **CAP**.  (2) ***CB*** has a cylindrical shape, with diameter identical to its host **CAP** and length proportional to local blood flow velocity. A certain capillary segment contains ***CBs*** of equal length. Different vessels have ***CB*** of different lengths proportional to flow velocity. | (1) A ***CB*** conveys *O_2_* to its downstream counterpart, representing advection. |
