# Supplementary material for: Progression of Diabetic Capillary Occlusion: A Model
Source: PLoS Comput Biol. 2016 Jun 14;12(6):e1004932. doi: 10.1371/journal.pcbi.1004932 (PMC4907516; doi:10.1371/journal.pcbi.1004932)
Supplement: S2 Table — (DOCX) [file pcbi.1004932.s019.docx]

**S2 Table. State transitions of model objects**

| Objects | State transition | Condition |
| --- | --- | --- |
| Mueller cell (**MC**) | $normal\underset{\to}{P_{O_{2}}^{(i)}}hypoxic$ | $P_{O_{2}}^{(i)}<P_{O_{2}}^{hyp}$ |
|  | $hypoxic\underset{\to}{P_{O_{2}}^{(i)}}normal$ | $P_{O_{2}}^{(i)}>P_{O_{2}}^{hyp}$ |
| Capillary Block (**CAP**) | $\left\{ \begin{aligned} normal \\ leaky \end{aligned} \right.\underset{\to}{{vol}^{(i)}\cdot c_{VEGF}^{(i)}, u_{kl}}occluded$ | $p_{occ}^{(i)}>\varepsilon$  where $p_{occ}^{(i)}=\frac{{vol}^{(i)}\cdot c_{VEGF}^{(i)}}{m_{VEGF}^{thr}+{vol}^{(i)}\cdot c_{VEGF}^{(i)}}\cdot\frac{{{(u}^{thr})}^{2}}{{{(u}^{thr})}^{2}+{{(u}^{kl})}^{2}}$, $\varepsilon$ is a random number in $(0,1)$ and $kl$ is a certain capillary segment. |
|  | $normal\underset{\to}{{vol}^{(i)}\cdot c_{VEGF}^{(i)}}leaky$ | ${vol}^{(i)}\cdot c_{VEGF}^{\left( i \right)}>m_{VEGF}^{thrE}$ |

Notations:

1. The superscript or subscript without () or [] such as *kl* represents a capillary segment or topological edge between junction *k* and junction *l*. It’s primarily used in the calculation of network flow.
2. The superscript or subscript with () such as (*i*) represents ids of objects **MC**, **OT**, **CAP** and **FP**. Each of these objects has unique id. When (*i*) and (*j*) pair up, it stands for a quantity between two object neighbors such as common surface area or distance between centers. It’s used in the simulation of oxygen and VEGF fluxes, except oxygen advection.
