# Supplementary material for: Progression of Diabetic Capillary Occlusion: A Model
Source: PLoS Comput Biol. 2016 Jun 14;12(6):e1004932. doi: 10.1371/journal.pcbi.1004932 (PMC4907516; doi:10.1371/journal.pcbi.1004932)
Supplement: S3 Table — (DOCX) [file pcbi.1004932.s020.docx]

**S3 Table. Processes and dynamics of field flux**

| Field | Process | Dynamics |
| --- | --- | --- |
| Oxygen (*O_2_*) | $M_{lm}^{[g]}\underset{\to}{advection}M_{lm}^{[g+1]}$ | $M_{lm}^{\left[ g+1 \right]}\left( t+\Delta t_{f} \right)=M_{lm}^{\left[ g \right]}\left( t \right);0\leq g<n_{lm}$ |
|  | $c_{O_{2}}^{(i)}\underset{\to}{diffusion}c_{O_{2}}^{(j)}$ | ${\Delta c}_{O_{2}}^{(i)}={-\Delta t}_{f}\cdot\sum_{(j)} \alpha\cdot D_{O_{2}}^{pl}\cdot\left( P_{O_{2}}^{(i)}-P_{O_{2}}^{(j)} \right)\cdot{\Delta S}_{(i)(j)}/{\Delta d}_{(i)(j)}$ |
|  | $c_{O_{2}}^{(i)}\underset{\to}{metabolism}$ | ${\Delta c}_{O_{2}}^{(i)}=\left\{ \begin{aligned} 0, if P_{O_{2}}^{(i)}<P_{O_{2}}^{hyp} \\ {-\Delta t}_{f}\cdot\frac{M_{0}\cdot P_{O_{2}}^{(i)}\cdot{vol}^{(i)}}{P_{O_{2}}^{(i)}+P_{{O_{2}}_{0}}}, if P_{O_{2}}^{(i)}\geq P_{O_{2}}^{hyp} \end{aligned} \right.$ |
| Vascular Endothelial Growth Factor (*VEGF*) | $c_{VEGF}^{(i)} \underset{\to}{diffusion}c_{VEGF}^{(j)}$ | ${\Delta c}_{VEGF}^{(i)}={-\Delta t}_{f}\cdot\sum_{(j)} D_{VEGF}^{tis}\cdot\left( c_{VEGF}^{(i)}-c_{VEGF}^{(j)} \right)\cdot{\Delta S}_{(i)(j)}/{\Delta d}_{(i)(j)}$ |
|  | $\underset{\to}{synthesis}c_{VEGF}^{(i)}$ | ${\Delta c}_{VEGF}^{(i)}={\Delta t}_{f}\cdot k_{VEGF}^{prod}\cdot\frac{m_{VEGF}^{max}-{vol}^{(i)}\cdot c_{VEGF}^{(i)}}{m_{VEGF}^{max}}\cdot\frac{e^{100\cdot(P_{O_{2}}^{hyp}-P_{O_{2}}^{(i)})}}{e^{100\cdot(P_{O_{2}}^{hyp}-P_{O_{2}}^{(i)})}+1}$ |
|  | $c_{VEGF}^{(i)}\underset{\to}{decay}$ | ${\Delta c}_{VEGF}^{(i)}={-\Delta t}_{f}\cdot k_{VEGF}^{dec}\cdot c_{VEGF}^{(i)}$ |

Notations:

1. The superscript or subscript without () or [] such as *lm* represents a capillary segment or topological edge between junction *l* and junction *m*. It’s primarily used in the calculation of network flow.
2. The superscript or subscript with () such as (*i*) represents ids of objects **MC**, **OT**, **CAP** and **FP**. Each of these objects has unique id. When (*i*) and (*j*) pair up, it stands for a quantity between two object neighbors such as common surface area or distance between centers. It’s used in the simulation of oxygen and VEGF fluxes, except oxygen advection.
3. The superscript or subscript with [] such as [g] represents position of object ***CB*** on a capillary segment. It’s used in the simulation of oxygen advection.
