# Supplementary material for: Progression of Diabetic Capillary Occlusion: A Model
Source: PLoS Comput Biol. 2016 Jun 14;12(6):e1004932. doi: 10.1371/journal.pcbi.1004932 (PMC4907516; doi:10.1371/journal.pcbi.1004932)
Supplement: S4 Table — (DOCX) [file pcbi.1004932.s021.docx]

**S4 Table. Model Parameters**

1. **Module Parameters**

| Module | Parameter | Value | Meaning | Source |
| --- | --- | --- | --- | --- |
| Oxygen Flux | $D_{O_{2}}^{pl}$ | $2.77\times{10}^{-5} {{cm}^{2}}/s$ | Diffusion coefficient of oxygen in blood plasma | [1] |
|  | $D_{O_{2}}^{tis}$ | $1.04\times{10}^{-5} {{cm}^{2}}/s$ | Diffusion coefficient of oxygen in tissue | [1] |
|  | $\alpha$ | $3\times{10}^{-5} {ml O_{2}}/{({cm}^{3}\cdot mmHg)}$ | Solubility of oxygen in blood and tissue | [1] |
|  | $M_{0}$ | $2\times{10}^{-4} {ml O_{2}}/{({cm}^{3}\cdot s)}$ | Maximum metabolic rate of oxygen in Müller cells and other retinal cells | [1] |
|  | $P_{{O_{2}}_{0}}$ | $1 mmHg$ | Oxygen tension when metabolic rate of Müller cells reach half of maximum value | [1] |
|  | $P_{O_{2}}^{hyp}$ | $4 mmHg$ | Critical oxygen tension when Mueller cells switch between normal and hypoxic state | Selected so no cells are hypoxic under normal conditions |
|  | $P_{O_{2}}^{art}$ | $45 mmHg$* | Oxygen tension in the inflow blood | Estimate based on  [2] |
|  | $P_{O_{2}}^{faz}$ | $45 mmHg$ | Oxygen tension at foveal avascular zone | Estimate based on [2] |
| VEGF Flux | $D_{VEGF}^{tis}$ | $5\times{10}^{-6} {{cm}^{2}}/s$ | Diffusion coefficient of VEGF in tissue | [3] |
|  | $k_{VEGF}^{dec}$ | $2.8\times{10}^{-4} 1/s$ | Decay rate of VEGF | [4] |
|  | $k_{VEGF}^{prod}$ | $10 1/s$ | Maximum synthesis rate of VEGF in Müller cells | Assumed, see Fig. S16 heat map |
|  | $m_{VEGF}^{max}$ | $1 (arbitrary unit)$ | Capacity of VEGF synthesis in Müller cells | Assumed, feedback loop |
| Network Flow | $P_{b}^{art}$ | $25 mmHg$** | Arteriolar pressure | Chosen to be within experimental physiological ranges reported by [5] ( 15-32 mmHg ) and [6] (11.3-26.3 mmHg) |
|  | $P_{b}^{ven}$ | $20 mmHg$*** | Venular pressure | Chosen to be slightly above mean intraocular pressure of 15 mmHg [6] |
|  | $\eta_{pl}$ | $1.05 cP$ | Viscosity of blood plasma | [1] |
| Occlusion | $m_{VEGF}^{thr}$ | $0.5 (arbitrary unit)$ | Critical VEGF level to induce occlusion | Assumed, see Fig. S16 heat map |
|  | $u^{thr}$ | $100 {\mu m}/s$ | Critical blood flow velocity to induce occlusion | Assumed, see Fig. S16, heat map |
| Edema formation | $m_{VEGF}^{thrE}$ | 0.01$(arbitrary unit)$ | VEGF threshold to trigger formation of edema | Assumed, see Fig. S16 heat map |

* Lower oxygen tension ($40 mmHg$) was assumed for peripheral simulation. ** Lower arterial pressure ($23 mmHg$) was assumed for peripheral simulation. *** Higher venular pressure ($22 mmHg$) was assumed for peripheral simulation.

1. **Geometrical parameters**

| Topology | Parameter | Value | Meaning | Source |
| --- | --- | --- | --- | --- |
| All | $\mu m per px$ | $2 \mu m/pixel$ | Unit conversion from pixel to micron | Selected to balance spatial resolution with computational cost |
| All | $a^{\mathrm{MC}}$ | $24 \mu m$ | Typical size of Mueller cells | Anatomically reasonable [7] |
| All | $a^{\mathrm{OT}}$ | $20 \mu m$ | Typical size of other retinal cells | Anatomically reasonable [7] |
| All | ${vol}^{\mathrm{FP}}$ | $3200 {\mu m}^{3}$ | Volume of fluid portion extracted in total from leaky capillary during ${\Delta t}_{e}$ | Assumed, because of lack of clinical data on rate of edema formation |
| Peri-foveal | $x^{dim}$ | $510 \mu m$ | Dimension in $\vec{x}$ direction | Measured from ASOLO image |
|  | $y^{dim}$ | $600 \mu m$ | Dimension in $\vec{y}$ direction | Measured from ASOLO image |
|  | $z^{dim}$ | $50 \mu m$ | Dimension in $\vec{z}$ direction | Selected to contain tissue layer of interest while excluding additional tissue layers |
|  | $d^{art}$ | $9 \mu m$ | Diameter of arterole | Measured from ASOLO image |
|  | $d^{ven}$ | $10 \mu m$ | Diameter of venule | Measured from ASOLO image |
|  | $d^{cap}$ | $5 \mu m$ | Average diameter of capillary | Measured from ASOLO image |
| Peripheral | $x^{dim}$ | $900 \mu m$ | Dimension in $\vec{x}$ direction | From [8] |
|  | $y^{dim}$ | $460 \mu m$ | Dimension in $\vec{y}$ direction | From [8] |
|  | $z^{dim}$ | $50 \mu m$ | Dimension in $\vec{z}$ direction | Selected to contain tissue layer of interest while excluding additional tissue layers |
|  | $d^{art}$ | $25 \mu m$ | Diameter of arteriole | From [8] |
|  | $d^{ven}$ | $30 \mu m$ | Diameter of venule | From [8] |
|  | $d^{shunt}$ | $18 \mu m$ | Diameter of shunt | From [8] |
|  | $d^{cap}$ | $10 \mu m$ | Average diameter of capillary | From [8] |
| Hexagonal | $x^{dim}$ | $510 \mu m$ | Dimension in $\vec{x}$ direction | Measured from ASOLO image |
|  | $y^{dim}$ | $600 \mu m$ | Dimension in $\vec{y}$ direction | Measured from ASOLO image |
|  | $z^{dim}$ | $50 \mu m$ | Dimension in $\vec{z}$ direction | Selected to contain tissue layer of interest while excluding additional tissue layers |
|  | $d^{art}$ | $9 \mu m$ | Diameter of arteriole | Measured from ASOLO image |
|  | $d^{ven}$ | $10 \mu m$ | Diameter of venule | Measured from ASOLO image |
|  | $d^{cap}$ | $5 \mu m$ | Average diameter of capillary | Measured from ASOLO image |

1. **Temporal parameters**

| Parameter | Value | Meaning | Source |
| --- | --- | --- | --- |
| $\sec perMCS$ | $86400 s/MCS$ | Unit conversion from Monte Carlo step to second | Selected, see Parameter selection section of Text S1 |
| ${\Delta t}_{o}$ | $4 weeks$ | Minimal time difference between two continuous capillary occlusion steps | Selected, see Parameter selection section of Text S1 |
| ${\Delta t}_{e}$ | $1 week$ | Minimal time difference between two continuous edema formation steps | Selected, see Parameter selection section of Text S1 |
| ${\Delta t}_{f}$ | $0.002 s$ | Time step for simulating modules of field fluxes | Selected for proper numerical integration |

**References**

1. Reglin B, Secomb TW, Pries AR. Structural adaptation of microvessel diameters in response to metabolic stimuli: where are the oxygen sensors? Am J Physiol Heart Circ Physiol. 2009;297(6):H2206-19.
2. Lau JC, Linsenmeier RA. Oxygen consumption and distribution in the Long-Evans rat retina. Experimental eye research. 2012;102:50-8.
3. Aubert M, Chaplain MA, McDougall SR, Devlin A, Mitchell CA. A continuum mathematical model of the developing murine retinal vasculature. Bull Math Biol. 2011;73(10):2430-51.
4. Shirinifard A, Glazier JA, Swat M, Gens JS, Family F, Jiang Y, et al. Adhesion failures determine the pattern of choroidal neovascularization in the eye: a computer simulation study. PLoS Comput Biol. 2012;8(5):e1002440.
5. Landis EM, Pappenheimer JR. Exchange of substances through the capillary walls. In: Hamilton WF, editor. Handbook of physiology Circulation. Washington D.C.: American Physiological Society; 1963. p. 961-1034.
6. Gooding KM, Tooke JE, von Lany H, Mitra M, Ling R, Ball CI, et al. Capillary pressure may predict preclinical changes in the eye. Diabetologia. 2010;53(9):2029-35
7. Reichenbach A, Bringmann A. Mueller cells in the healthy and diseased retina. New York: Springer 2010. 415 p.
8. Spitznas M, Bornfeld N. The architecture of the most peripheral retinal vessels. Albrecht Von Graefes Arch Klin Exp Ophthalmol. 1977;203(3-4):217-29.
