# Supplementary material for: Progression of Diabetic Capillary Occlusion: A Model
Source: PLoS Comput Biol. 2016 Jun 14;12(6):e1004932. doi: 10.1371/journal.pcbi.1004932 (PMC4907516; doi:10.1371/journal.pcbi.1004932)
Supplement: S1 Text — (DOCX) [file pcbi.1004932.s022.docx]

**S1 Text: DOCUMENTATION OF DETAILED DESCRIPTION OF THE MODEL**

**Network flow**

To calculate the blood flow velocities within a capillary segment *lm* (segment refers to capillary vessel between two branch junctions *l* and *m*), we applied Poiseuille equation which relates the flow resistance to capillary diameter, length and the apparent viscosity:

|  | $\dot{Q_{lm}}=\frac{\pi(P_{l}-P_{m})D_{lm}^{4}}{128\eta_{lm}L_{lm}}$ | (1) |
| --- | --- | --- |

where $\dot{Q_{lm}}$ is the volume flow rate from branching node $l$ to m; $P_{l}$is hydrostatic pressure at junction *l*, $P_{m}$ is the hydrostatic pressure at *m*; $D_{lm}$is intraluminal diameter of the segment; $\eta_{lm}$is the effective viscosity of blood within this segment; $L_{lm}$is the length of the segment.

Pries AR *et al.* summarized a relation of *in vivo* effective viscosity with vessel segment diameter and hematocrit by studying *in vivo* rheology of blood [1-3]. The proposed relation is in agreement with Fåhræus–Lindqvist effect which describes decreasing effective viscosity with decreasing diameter down to approximately$10 \mu m$ and inverse Fåhræus–Lindqvist effect which describes increasing effective viscosity with decreasing diameter for smaller diameters than $10 \mu m$. The relation of *in vitro* apparent viscosity with tube diameter of segment *lm* and hematocrit is given as [3]:

| $\eta_{vitro}=1+(\eta_{0.45}-1)\cdot\frac{\left( 1-H_{D} \right)^{C}-1}{\left( 1-0.45 \right)^{C}-1}$ | (2) |
| --- | --- |

where the $C$ and $\eta_{0.45}$ are calculated respectively as

| $C=\left( 0.8+e^{-0.075D_{lm}} \right)\cdot\left( -1+\frac{1}{1+{10}^{-11}{\cdot D}_{lm}^{12}} \right)+\frac{1}{1+{10}^{-11}{\cdot D}_{lm}^{12}}$ | (3) |
| --- | --- |
|  |  |
| $\eta_{0.45}=220e^{-1.3D_{lm}}+3.2-2.44e^{-0.06{D_{lm}}^{0.645}}$ | (4) |

Equation (2) would give *in vitro* effective viscosity. According to Pries AR *et al.*, flow resistance *in vivo* can be explained by *in vitro* viscosity and the effect of an endothelial surface layer that impedes blood flow [3, 4]. Then *in vivo* viscosity is calculated as$\eta_{vivo}=\eta_{vitro}\cdot{({D_{lm}}/{D_{lm}^{eff}})}^{4}$, where$D_{lm}^{eff}$is effective diameter of this segment, as calculated from equations (8)-(11) in [3]. We assumed that hematocrit in each capillary segment maintained the value of 0.45 in all calculations though plasma skimming is a known phenomenon which would progressively elevate hematocrit along an arteriole.

Our model also includes the structural adaptation module which adjusts effective diameters of all capillaries slightly after each occlusion. Reglin *et al.* proposed the convergence-aimed method described in [5] to adapt diameters in response to hemodynamic and metabolic stimuli with the form of$\Delta D=\Delta t\cdot S_{tot}\cdot D$, where $\Delta t=0.5\cdot\Delta t_{f}$ in the model and $S_{tot}=k_{h}\left( S_{\tau}+k_{p}S_{p} \right)+k_{m}\left( S_{m}+k_{c}S_{c} \right)-k_{s}$ is a combination of four stimuli: transmural pressure$S_{P}=-log(100-86e^{-5000{(\log\left( \log\left( P \right) \right))}^{5.4}})$, shear stress$S_{\tau}=log(\tau+0.103)$, oxygen tension$S_{m}=log(1+{1.38L(1-{P_{O_{2}}}/{P_{O_{2}}^{ref}})}/{(\dot{Q}+\dot{Q}_{ref})})$, and conducted signal$S_{c}$ (see equations (15)-(17) in [5]). $k_{h}, k_{p}, k_{m},k_{c}$ and $k_{s}$ are five coefficients, with values 1, 0.2, 1, 1.6 and 2 respectively. Stimulus caused by transmural pressure $S_{P}$, which is assumed to decrease sigmoidally with increase of transmural pressure, decreases diameter of the capillary. Increased stimulus caused by shear stress$S_{\tau}$ is assumed to result in an increase of diameter. We considered the average oxygen tension of all **CAP** objects in each capillary segment as the metabolic signal, with $P_{O_{2}}^{ref}=100mmHg$ and $\dot{Q}_{ref}=0.2pL/s$ in our model i.e. the lower the oxygen tension, the larger the metabolic stimulus. Increased stimulus caused by the metabolic signal $S_{m}$, the average oxygen tension in a capillary segment in our model, is assumed to increase diameter. The fourth component of the total stimulus is conducted signal of metabolic information $S_{c}$ generated in a segment that affects an upstream segment [5]. We want to reiterate that this module is not our focus in the model and the inclusion of the module is for qualitative description of the structural adaptation that can exist following capillary occlusions. We included all components of stimuli that contributed to adaptation of segment diameters but restricted the effect of adaption in the model by setting a relatively small$\Delta t$. Following each occlusion, we executed the adaptation module for only one cycle of $\Delta t$. As we observed, maximum diameter change due to such one-step adaptation is about 2%. Again, the purpose of this module is to make qualitative sense of diameter adaptation in response to hemodynamic and metabolic stimuli following updated distribution of flow velocities and oxygen tension. The extent to which such adaptation affects the progression of diabetic capillary occlusions remains clinically unclear, though given that IRMA are found surrounding local ischemic areas, there is likely at least some role. Future elaboration of the current model with more available experimental data on diameters and flow velocities will include a more realistic adaptation module.

For readers with interest, authors in [5] applied iterations of diameter change until a steady state of diameters was reached, which results in the ultimate velocity distribution of the network. This distribution of velocity is then validated against experimental measurement, and the goodness of validation is considered for improvement of coefficients that combine the different stimuli.

**Oxygen flux**

In the blood, oxygen exists in three compartments: hemoglobin-bound in red blood cells (RBCs), unbound in RBCs and unbound in plasma. Oxygen tension within blood, $P_{O_{2}}$ ,has the following relationship with oxygen concentration$c_{O_{2}}$ [5]:

| $c_{O_{2}}=H_{D}\cdot c_{Hb}\cdot s_{O_{2}}+H_{D}\cdot\alpha_{RBC}\cdot P_{O_{2}}+(1-H_{D})\cdot\alpha\cdot P_{O_{2}}$ | (5) |
| --- | --- |

where each term in the summation corresponds to one of the three compartments. In the first term, $c_{Hb}$ is the concentration of hemoglobin within the RBC, and $s_{O_{2}}$ is the saturation of oxygen binding to hemoglobin, which acquires a form of Hill’s function$s_{O_{2}}={{P_{O_{2}}}^{n}}/{({P_{O_{2}}}^{n}+{P_{O_{2}}}_{50}^{n})}$, where $n=3$ and oxygen tension at half-maximal hemoglobin saturation is${P_{O_{2}}}_{50}=38 mmHg$. In the second term, $\alpha_{RBC}$ is the solubility of unbound oxygen in RBCs. In the third term, $\alpha$ is the solubility of free oxygen in plasma.

In tissue, we assumed oxygen solubility was equal to$\alpha$. Then the relationship between oxygen tension and oxygen concentration follows Henry’s law $c_{O_{2}}=\alpha\cdot P_{O_{2}}$.

Diffusion of oxygen is modeled between all four cell types (**CAP, FP, MC, OT**), using a coarse-grained cell-to-cell transfer with the assumption that within each cell the oxygen tension is uniform. Within a short period of time$\Delta t_{f}$, the change of volume of oxygen within cell$(i)$due to cell-to-cell transfer is calculated as:

| ${\Delta v}_{O_{2}}^{(i)}=-\Delta t_{f}\cdot\sum_{(j)}^{N_{nb}^{(i)}} \frac{A_{(i)(j)}\cdot D_{O_{2}}\cdot\alpha\cdot(P_{O_{2}}^{\left( i \right)}-P_{O_{2}}^{(j)})}{d_{(i)(j)}}$ | (6) |
| --- | --- |

where ${\Delta v}_{O_{2}}^{(i)}$ is the change of oxygen volume in cell$(i)$. Bracketed notation $(i)$used in superscripts and subscripts represents cell ids, distinguished from unbracketed $i$ representing node ids of the capillary network, which applies to all following equations. On the right hand of the equation, $\Delta t_{f}$ is the time step of integration for simulation of oxygen and VEGF fluxes; $A_{(i)(j)}$ is the contact surface area between the pair of cell neighbors $(i)$ and$(j)$; $d_{(i)(j)}$ is the distance between centers of mass of cell $(i)$ and cell$(j)$; and the summation iterates over all of cell $(i)$’s neighbors in contact. $D_{O_{2}}$ is equal to$D_{O_{2}}^{pl}$ only if $(i)$ is **CAP** and $D_{O_{2}}^{tis}$ otherwise .

For cells near the foveal avascular zone, there exists additional oxygen flux coming from the FAZ, assumed to be supplied by choroidal capillaries. In the model, the FAZ is treated as the whole side of a three dimensional system. If a cell touches this side, it receives oxygen from the FAZ. We assume that the FAZ has a constant oxygen tension$P_{O_{2}}^{faz}$, and additional change in oxygen volume within cell$(i)$ due to FAZ-to-cell transfer is calculated as:

| ${\Delta v'}_{O_{2}}^{(i)}=-\Delta t_{f}\cdot\frac{A_{(i)}^{faz}\cdot D_{O_{2}}^{tis}\cdot\alpha\cdot(P_{O_{2}}^{\left( i \right)}-P_{O_{2}}^{faz})}{d_{(i)}^{faz}}$ | (7) |
| --- | --- |

where ${\Delta v'}_{O_{2}}^{(i)}$ is the additional change of oxygen volume in cell $(i)$ due to its contact with FAZ and ${(\Delta v}_{O_{2}}^{(i)}+{\Delta v'}_{O_{2}}^{(i)})$ gives total change of oxygen volume for such cells; $A_{(i)}^{faz}$ is the contact surface area between FAZ and cell$(i)$*,* which, for simplicity, is approximated to be one sixth of the cell surface area of cell$\left( i \right)$; $d_{(i)}^{faz}$ is the distance from the center of cell $\left( i \right)$ to the FAZ.

Consumption of oxygen is modeled for two cell types (**MC, OT**). In order to a capture cell’s capability to adapt its demand for oxygen according to available oxygen supply, we applied Michaelis-Menten type kinetics to model oxygen consumption. Within a short period of time$\Delta t_{f}$, the change of volume of oxygen within cell$(i)$resulting from consumption is calculated as:

| ${\Delta v}_{O_{2}}^{(i)}=-\Delta t_{f}\cdot\frac{M_{0}\cdot P_{O_{2}}^{\left( i \right)}}{P_{O_{2}}^{\left( i \right)}+P_{{O_{2}}_{0}}}$ | (8) |
| --- | --- |

where $M_{0}$ is the maximum oxygen consumption rate of cells; $P_{{O_{2}}_{0}}$ is the oxygen tension at which cells acquire the half maximum oxygen consumption rate.

We propose a conveyor-belt like method to model the oxygen advection (Fig. 4, 5). One advantage is that each capillary segment can be flexibly re-discretized without interfering with diffusion module. This is useful when capillary occlusion occurs and flow velocities on other patent capillary segments change. We describe in a relatively more detailed way how the advection module functions and is linked with the diffusion module as follows.

The module of oxygen advection is modeled for object ***CB***, and oxygen volumes are converted between the ***CB*** and the **CAP** during each iteration of the simulation of fluxes. While **CAP** is the structural element (visually seen in the model configuration) of a capillary segment, ***CB*** is the functional element (visually hidden) for oxygen advection. On a certain capillary segment$jk$, the model discretizes the segment into a sequence of ***CB***s, each with size$a_{jk}=u_{jk}\cdot\Delta t_{f}$, where$u_{jk}={\dot{Q_{ij}}}/{{(0.25\pi\cdot D}_{ij}^{2})}$ is flow velocity on the segment. Thus, once oxygen in a ***CB*** is transferred to the closest downstream ***CB***, oxygen moves with the speed of blood flow in that capillary segment. The slower the flow velocity on a capillary segment, the smaller each ***CB*** and the slower the advection of oxygen. All ***CB***s on the same capillary segment have the same size, and the quantity of ***CBs*** on a capillary segment is simply the length of capillary segment divided by size of a single ***CB***. The mapping of ***CBs*** to a **CAP** is decided by the center position of a ***CB*** after discretization. As long as the center of a ***CB*** is within the extent of a **CAP**, this ***CB*** belongs to this **CAP**. As an example, the first **CAP** contains first and second ***CB*** on segment$jk$, and the second **CAP** the third and fourth ***CB***, and the last ***CAP*** the ${(n-1)}^{th}$ and $n^{th}$ ***CB*** (Fig 4). During a small period of time$\Delta t_{f}$ that involves advection and diffusion, two intermediate steps are needed to convert oxygen volumes between a **CAP** and its associated ***CB***s (Fig 5): (1) modeling advection of oxygen volumes in ***CB***s on each capillary segment at time step$t_{0}$; (2) summation of oxygen volumes at ***CB***-level to update **CAP**-level immediately after advection at time step$t_{0}$; (3) modeling diffusion between objects as described above; (4) subtraction of **CAP**-level diffused oxygen volumes from associated ***CB***s immediately after diffusion at time step$t_{0}$.

On a certain capillary segment$jk$which has $n_{jk}$***CB***s, ***CB***s$\left\{ 0,1,2,\ldots, n_{jk}-1 \right\}$ carry oxygen volumes$\left\{ M_{jk}^{[0]},M_{jk}^{[1]}, M_{jk}^{[2]}, \ldots, M_{jk}^{\left[ n_{jk}-1 \right]} \right\}$, where superscript$[i]$ refers to ranking of the ***CB*** on the segment. Following three equations are used for the simulation of advection (Fig. 4):

| $M_{jk}^{\left[ i+1 \right]}\left( t+\Delta t_{f} \right)=M_{jk}^{\left[ i \right]}\left( t \right);0\leq i<n_{jk}-1$ | (9) |
| --- | --- |

| $\left\{ \begin{aligned} M_{kr}^{\left[ 0 \right]}\left( t+\Delta t_{f} \right)=M_{jk}^{\left[ n_{jk}-1 \right]}\left( t \right)\cdot\frac{S_{kr}\cdot u_{kr}}{S_{jk}\cdot u_{jk}} \\ M_{ks}^{\left[ 0 \right]}\left( t+\Delta t_{f} \right)=M_{jk}^{\left[ n_{jk}-1 \right]}\left( t \right)\cdot\frac{S_{ks}\cdot u_{ks}}{S_{jk}\cdot u_{jk}} \end{aligned} \right.$ | (10) |
| --- | --- |
| $M_{jk}^{\left[ 0 \right]}\left( t+\Delta t_{f} \right)=M_{xj}^{\left[ n_{xj}-1 \right]}\left( t \right)+M_{yj}^{\left[ n_{yj}-1 \right]}\left( t \right)$ | (11) |

Equation (9) describes advection on a capillary segment, using$jk$ as an example. Equation (10) describes the distribution of oxygen volumes at a junction that bifurcates a parent capillary segment into two daughter capillary segments, using junction$k$ as an example. Equation (11) describes the summation of oxygen volumes at a junction that merges two predecessor capillary segments into one successor segment (Fig. 4). In equation (9), $n_{jk}-1$is the ranking of last ***CB*** on segment$jk$. This equation (9) describes the process needed to “convey” oxygen volume from a ***CB*** to the closest downstream neighbor ***CB***. In equation (10),$M_{jk}^{\left[ n_{jk}-1 \right]}$ is the oxygen volume in the last ***CB*** on parent capillary segment$jk$ and$M_{ks}^{\left[ 0 \right]}$ is oxygen volume in the first ***CB*** on one daughter capillary segment, $ks$ in this case. $S_{jk}\cdot u_{jk}$and $S_{ks}\cdot u_{ks}$ are the volumes of blood flow exiting segment$jk$ and entering segment$ks$ per unit time respectively. Based on conservation of volume of blood flow at the junction$k$ and assumption that oxygen tension remain constant at the *instant* of blood flow leaving parent segment and entering two daughter segments, the *first* ***CB*** on segment $ks$ receives a fraction of oxygen volume from *last* ***CB*** on segment$jk$, with the fraction equal to${(S}_{ks}\cdot u_{ks})/(S_{jk}\cdot u_{jk})$. The same rule of distribution of oxygen volume into segment$jk$ is applied. In equation (11), $M_{jk}^{\left[ 0 \right]}$ is the oxygen volume in the first ***CB*** on successor capillary segment$jk$ and$M_{xj}^{\left[ n_{xj}-1 \right]}$ is oxygen volume in the last ***CB*** on one predecessor capillary segment, $xj$ in this case. According to conservation of volume of blood flow at the instant of merging flow,$M_{jk}^{\left[ 0 \right]}$ is equal total amount of$M_{xj}^{\left[ n_{xj}-1 \right]}$and$M_{yj}^{\left[ n_{yj}-1 \right]}$. General extensions can be made for junctions with N daughter segments and M parent segments with N and M greater than 1, using the combination of rules for branching and merging situation.

An example of the conveyor-belt model of oxygen advection is shown in Fig. 5. Three consecutive time steps are looked at. For purpose of clarity, within each time step only modules of oxygen advection and diffusion are included in the example and a simple route of diffusing flux (shown in black arrow) from the **CAP** (shown in light red) to **OT** (shown in light brown) is assumed. In addition, to emphasize the change of oxygen volumes in a given step, we highlight numbers in bold. During the first time step from $t_{0}$ to $t_{0}+\Delta t$, while the second **CAP** and its associated ***CB***s still have zero oxygen volume, the first **CAP** and associated ***CB***s undergo (1) a process of advection that passes 1 unit oxygen volume (an example of an amount conveyed from upstream ***CB*** not shown) to the first ***CB***, while **CAP**s are not involved in advection; (2) an intermediate step that updates **CAP**’s pre-diffusion oxygen volume by adding 1 (its first associated ***CB***) and 0 (its second associated ***CB***); (3) the process of diffusion that delivers 0.2 to **OT** in contact (amount assumed for convenience in this example, and again only a simple **CAP**🡪**OT** diffusion is considered in this example) and **CAP**’s post-diffusion oxygen volume becomes 0.8, while ***CB***s are not involved in diffusion; (4) an ultimate step that updates **CAP**’s associated ***CB***s’ oxygen volumes by subtracting diffused amount 0.2/1=20% (diffused/pre-diffusion), the first CB thus having 0.8 oxygen volume now. During the second time step from$t_{0}+\Delta t$ to $t_{0}+2\Delta t$, a similar verbal “simulation” proceeds. (1) process of advection takes place as another 1 oxygen volume is passed to first ***CB*** and 0.8, previously held by the first ***CB***, passed to the second ***CB***; (2) an intermediate step adds 1 and 0.8 to first **CAP**, but still none for the second **CAP**; (3) process of diffusion updates the first **CAP**’s oxygen volume to 1.44, with 0.36 (chosen for convience in this example) diffused out; (4) an ultimate step updates oxygen volumes in both of **CAP**’s two ***CB***s, again by subtracting diffused fraction 0.36/1.8=20%. During the third time step from$t_{0}+2\Delta t$ to $t_{0}+3\Delta t$, advection now passes an oxygen volume of 0.64, previously held by the second CB, into the third ***CB***, which is associated with the second **CAP**. An intermediate step updates both **CAP**s by summing up oxygen volumes in their associated ***CB***s. The diffusion process now changes oxygen volumes of both **CAP**s, with the first and second diffusing out 0.36/1.8=20% and 0.1/0.64=15.625% respectively. An ultimate step subtracts oxygen volumes from their associated ***CB***s’ with the percent change.

**VEGF flux**

In our model, VEGF plays an important role in leading to occlusion of the capillary network. Specifically, VEGF synthesized and released by Mueller cells under hypoxic conditions is assumed to make a contribution in this model to both capillary occlusion and leakage. Physiologically, VEGF causes capillary occlusion indirectly by inducing ICAM expression on endothelial cells resulting in increased leukocyte leukostasis and capillary occlusion whereas in the model these intermediate steps are not treated. Edema, in the model, is considered a direct effect of elevated VEGF. In the current model, we neglect the synthesis of VEGF by model cell types other than **MC**. Also, advection of VEGF via blood flow was not treated.

Production of VEGF is modeled for one cell type (**MC**), with its synthesizing rate dependent on both current cellular VEGF level and oxygen tension. As the produced VEGF amount increases within a Mueller cell, the production rate drops corresponding to limited producing capacity of the cell partly owing to feedback signaling. In addition, the production rate is also directly dependent on a factor determined by a cellular oxygen tension threshold. When cellular oxygen tension is below a given hypoxic threshold, this factor rapidly approaches 1. During the period of time${\Delta t}_{f}$, the change in concentration of VEGF in cell $\left( i \right)$ is given by the following equation:

| ${\Delta c}_{VEGF}^{(i)}={\Delta t}_{f}\cdot k_{VEGF}^{prod}\cdot\frac{1}{{vol}^{(i)}}\cdot\frac{m_{VEGF}^{max}-{vol}^{(i)}\cdot c_{VEGF}^{(i)}}{m_{VEGF}^{max}}\cdot\frac{e^{100\cdot(P_{O_{2}}^{hyp}-P_{O_{2}}^{(i)})}}{e^{100\cdot(P_{O_{2}}^{hyp}-P_{O_{2}}^{(i)})}+1}$ | (12) |
| --- | --- |

where$k_{VEGF}^{prod}$ is production rate constant of VEGF; $m_{VEGF}^{max}$ is the capacity of VEGF production; $P_{O_{2}}^{hyp}$ is the threshold of oxygen tension separating normoxia and hypoxia; ${vol}^{(i)}$ is the volume of cell$\left( i \right)$; $c_{VEGF}^{(i)}$ is the cellular VEGF concentration and$P_{O_{2}}^{(i)}$ is the cellular oxygen tension.

Decay of VEGF is modeled for four cell types (**CAP, FP, MC, and OT**). During a short period of time${\Delta t}_{f}$, the change in concentration of cellular VEGF due to decay is described by the following equation:

| ${\Delta c}_{VEGF}^{(i)}={-\Delta t}_{f}\cdot k_{VEGF}^{dec}\cdot c_{VEGF}^{(i)}$ | (13) |
| --- | --- |

where$k_{VEGF}^{dec}$ is the decay rate constant of VEGF.

Diffusion of VEGF is modeled between certain pairs of the four cell types (**CAP, FP, MC, and OT**), which includes following fluxes: **MC**🡪**OT**, **OT**🡪**OT, FP🡪FP, OT🡨🡪FP,** **FP🡪CAP** and **OT**🡪**CAP**. Our model assumes that VEGF is not absorbed by **MC** and doesn’t exit **CAP** by advection. The governing equation for transfer of VEGF from cell $\left( i \right)$ is similar to that describing oxygen diffusion:

| ${\Delta c}_{VEGF}^{(i)}={-\Delta t}_{f}\cdot\sum_{(j)} \frac{{A_{(i)(j)}\cdot D}_{VEGF}^{tis}\cdot\left( c_{VEGF}^{(i)}-c_{VEGF}^{(j)} \right)}{d_{(i)(j)}}$ | (14) |
| --- | --- |

where $D_{VEGF}^{tis}$ is the approximated diffusion rate coefficient of VEGF within tissue space. And possible directions of $\left( i \right)$🡪$\left( j \right)$ obey the above regulations for transfer fluxes.

Similar to the situation for the diffusion of oxygen, there exists an additional flux for cells in contact with the FAZ, where FAZ serves as a sink for VEGF and it rapidly removes VEGF. The additional change in VEGF concentration within cell $\left( i \right)$when touching FAZ is calculated as:

| ${\Delta c'}_{VEGF}^{(i)}={-\Delta t}_{f}\cdot\frac{{A_{(i)}^{faz}\cdot D}_{VEGF}^{tis}\cdot c_{VEGF}^{(i)}}{d_{(i)}^{faz}}$ | (15) |
| --- | --- |
|  |  |

where$A_{(i)}^{faz}$ and $d_{(i)}^{faz}$ follow the same approximation rule as for oxygen diffusion.

**Capillary occlusion**

We use a probabilistic function to determine the occurrence of a capillary occlusion. Occlusion is an irreversible process in the model, since occluded capillaries would ultimately become acellular, likely resulting from biochemical interactions with the occluding leukocyte. Physiologically, there are recurrent temporary occlusions due to leukostasis which ultimately, through loss of endothelial cells and limits on their regeneration, result in irreversible capillary occlusion. The model only addresses this final capillary occluding event. The time interval between two events which calculate the probability of capillary occlusion is much greater than the time step of integration of the ODEs descriptive of oxygen and VEGF flux. In our model, judgement of capillary occlusion is made at the **CAP** level. Every period of time${\Delta t}_{o}$, the calculated probability of occlusion of each **CAP** cell (*i),* $p_{occ}^{(i)}$ ,is compared with a random number between 0 and 1. Occlusion occurs if the former is greater than the latter (S2 Table). If the occlusion decision is made, the diameter of the whole capillary segment *kl* that the **CAP** cell *(i)* belongs to will be set to an infinitesimal number (not zero because of division by zero issues). Mathematically this gives a huge resistance to blood flow as the equivalent of vascular obstruction. This probability function is related to both local VEGF level and the blood flow velocity, and has the following form:

| $p_{occ}^{(i)}=\frac{{vol}^{(i)}\cdot c_{VEGF}^{(i)}}{m_{VEGF}^{thr}+{vol}^{(i)}\cdot c_{VEGF}^{(i)}}\cdot\frac{{{(u}^{thr})}^{2}}{{{(u}^{thr})}^{2}+{{(u}^{kl})}^{2}}$ | (16) |
| --- | --- |

where$u^{thr}$ is a critical blood flow velocity,$m_{VEGF}^{thr}$ is a critical VEGF level, and ${vol}^{(i)}$is the volume of **CAP** (*i*).

The form of the occlusion probability function is chosen so that it has a sigmoid shape in response to each VEGF level and flow velocity. Higher VEGF level and lower flow velocity give greater occlusion probability. Exponents in the probability function control the steepness of sigmoid curve, while VEGF level equal to$m_{VEGF}^{thr}$ and flow velocity equal to $u^{thr}$correspond to the steepest part of the sigmoid curve. As discussed in the **Parameter selection and analysis of parameter influence** section below, a wide range of values are tested for$m_{VEGF}^{thr}$ and$u^{thr}$. Variation in each parameter greatly influences capillary network patency and retinal thickness.

**Edema formation**

Edema formation is triggered by elevation of VEGF above a threshold. In our model, we use a pseudo cell type fluid portion (**FP)** as the edema component. We assume that the **FP** is an object that barely spreads and is trapped by surrounding objects. We also assume that a patent (unoccluded) capillary segment becomes leaky if any of its member **CAP**s’ local VEGF level is greater than a threshold, i.e., ${vol}^{(i)}\cdot c_{VEGF}^{\left( i \right)}>m_{VEGF}^{thrE}$(S2 Table). Edema is formed only near the leaky site *(i)*. A **FP** is created nearby a leaky **CAP** at every ${\Delta t}_{e}$ as a visual representation of leaked fluid and is the cause of retinal thickening. In addition, a pumping mechanism, representing retinal pigment epithelial cell function, is added to eliminate the **FP**s only if they are large enough to physically touch the bottom boundary surface at$Z=0$. This reflects the role of the retinal pigment epithelial pumps to remove excess accumulated fluid.

The creation of **FP** is similar to initialization of cells at the start of the simulation. A voxel in contact with a randomly chosen surface voxel of the leaky **CAP** is selected as a seed for a **FP**. Then the Cellular Potts Model is implemented for expansion of the one-voxel seed to pre-defined size${vol}^{\mathrm{FP}}$. During the expansion, **FP** displaces surrounding cells to result in thickening of retinal tissue. According to the Cellular Potts model [6, 7], the growth of the FP seed and the pushing effect are effectively governed by minimization of the following Hamiltonian:

| $H=\sum_{i,j neighbors} J\left( \tau\left( \sigma\left( i \right) \right),\tau\left( \sigma\left( j \right) \right) \right)\cdot\left( 1-\delta\left( \sigma\left( i \right),\sigma\left( j \right) \right) \right)+\sum_{i} \lambda_{vol}\cdot{(vol\left( \sigma\left( i \right) \right)-{vol}^{tgt}(\sigma\left( i \right)))}^{2}$ | (17) |
| --- | --- |

where the first summation describes adhesion energy between cells and the second summation describes the volume constraint of cells.$\tau\left( \sigma\left( i \right) \right)$stands for cell type of cell$\sigma\left( i \right)$.$J\left( \tau\left( \sigma\left( i \right) \right),\tau\left( \sigma\left( j \right) \right) \right)$ is the adhesion energy for the two cells types between lattice sites$i,j$. The term$\left( 1-\delta\left( \sigma\left( i \right),\sigma\left( j \right) \right) \right)$ limits calculation of adhesion energy only between lattice sites representing different cells.$\lambda_{vol}$ specifies the strength of volume constraint. $vol\left( \sigma\left( i \right) \right)$ and ${vol}^{tgt}(\sigma\left( i \right))$ are present cell volumes and target cell volumes respectively.

In terms of edema formation,$\lambda_{vol}$ acquires a large value and${vol}^{tgt}$ is equal to ${vol}^{\mathrm{FP}}$so that a slight deviation from the target volume increases *H* significantly and thus the pseudo cells rapidly grow to the target size. In terms of fluid elimination at the bottom,${vol}^{tgt}$ is instead set to 0, which shrinks the **FP**. It should be noted that Cellular Potts Model is not essential for modeling the expansion of the **FP** seed to pre-defined size. However, the Cellular Potts model brings convenience to the process of surrounding cell rearrangement once **FP** is formed.

**Boundary conditions and initial state of simulation**

A few boundary conditions are imposed in the model. The first boundary condition assigns values for hydrostatic pressures and incoming oxygen tension of boundary nodes used by the network flow module. In terms of hydrostatic pressures, arteriolar node (**A**) has pressure$P_{b}^{art}$, venous node (**V**) has pressure$P_{b}^{ven}$, and all others have intermediate pressure values$\alpha\cdot P_{b}^{art}+(1-\alpha)\cdot P_{b}^{art}$ (S15 Fig). Three criteria (i), (ii), (iii) were considered when selecting pressure values for boundary nodes which are neither **A** or **V**. (i) we made an estimate of pressure values for boundary nodes depending on ratio of their topological distance from **A** and that from **V**: $\beta$. We define topological distance as the shortest path length between two nodes. We calculated pressure values based on topological distance, the smaller $\beta$ is, the higher the nodal pressure assigned, namely a greater$\alpha$ is used. (ii) We assumed that a capillary segment involving a boundary node is always a “daughter” edge of the relevant junction. For each junction in the network, there may exist “daughter” edges with a “parent” edge (in some places we alternatively use a “predecessor”-“successor” relationship if it’s a merging junction instead of branching junction). (iii) We assumed that if $\beta$ is small, a node connected with a boundary node is likely a branching junction where the flow velocity vector points to the boundary node representing outflow. In contrast, for nodes topologically closer to venous outlet node, it’s likely a merging junction where the flow velocity vector points from the boundary node representing inflow. During progression of capillary occlusions, all these pressure values were fixed. Refer to S15 Fig for a flow velocity map under normal condition with marked pressure values for all boundary nodes in CASE 1 and CASE 2. Similar rules are used for the boundary values of incoming oxygen tension within blood. Outgoing oxygen tension is not assigned but calculated from the steady state simulation. Arteriolar node (**A**) has pressure$P_{O_{2}}^{art}$ and all other inlet boundary nodes have intermediate pressure values$\alpha\cdot P_{O_{2}}^{art}+(1-\alpha)\cdot20$. We assume that cells don’t move outside any of the six boundary surfaces. Therefore, the second boundary condition is that there are no periodic boundary conditions. We assume that FAZ is a perfect stable oxygen source and VEGF sink. Therefore, the third boundary condition assigns a fixed oxygen tension of$P_{O_{2}}^{faz}$ and also assigns a zero VEGF level to the FAZ region.

The initial state of the model configuration is reconstructed as a vessel network with uniformly seeded cell centers, each occupying one voxel. The Cellular Potts model is implemented to grow one-voxel cells to proper size, i.e., $a^{\mathrm{MC}}$or$a^{\mathrm{OT}}$, in a similar fashion as discussed in **Edema formation** section. In addition, we assumed initially a low baseline level of VEGF (1% of$m_{VEGF}^{thr}$) in all cell types merely serving the purpose of representing initial diabetic physiological conditions and creating a very small but non-zero probability of capillary occlusion. Note that the diabetic likely has a significant elevation of leukocyte adhesion probability even in the first weeks of the diabetic state according to animal models, prior to any permanent capillary occlusion. However these leukocyte adhesion events do not result in permanent occlusions because the endothelial cell population has not yet been depleted by these recurrent events. The model deals only with the permanent occlusion events. In the model the initial oxygen tension (“initial” here means after construction of cells and vessels but before any simulation of fluxes) is zero everywhere, which will be updated after simulation of oxygen advection/diffusion/metabolism under normal condition.

**Parameter selection and analysis of parameter influence**

We divide parameters in the model into three general categories: geometrical parameters, temporal parameters and module parameters.

Geometrical parameters are selected based on either our imaging pictures (CASE 1, CASE 2 and Hexagonal network) or a published structural drawing (Peripheral network) [8]. Modeled cell sizes are selected to be anatomically reasonable (S4 Table) especially for the MCs and the CAPs. Conversion rate from pixels to microns is 2 micron per pixel, chosen with consideration of both computational cost and visualization resolution (S4 Table).

Temporal parameters are selected based on simulation requirements. The parameter${\Delta t}_{f}$ is the time step of integration for simulation of oxygen and VEGF fluxes. The criterion is that it should be small enough to ensure proper and stable integration of differential equations, and large enough to allow practical computation. All other temporal parameters were selected so that the model produces time scales comparable to clinical observations. In the future, temporal parameters could be adjusted when more clinical data becomes available. Conversion rate of MCS to seconds is 86,400 second per MCS is selected as long as equivalent time of 1 MCS is much greater than${\Delta t}_{f}$. We assume that it takes much longer to result in a new possible capillary occlusion than it does to simulate the steady state of fluxes. The former, from clinical experience and animal experimentation, ranges from days to weeks to years, while the latter occur in seconds.

Some module parameters were well studied in published studies, such as those related to oxygen and VEGF. Beyond these, we introduce some model-specific parameters that are not described elsewhere, such as $k_{VEGF}^{prod}$ involved in VEGF synthesis, $m_{VEGF}^{thr}$ and $u^{thr}$ involved in the calculation of occlusion probability, and $m_{VEGF}^{thrE}$ involved in edema formation. Such parameters were mainly selected for convenience in order to produce qualitatively comparable model outputs. Therefore, to get a better understanding of the influence of several important parameters on model outputs, we varied a number of parameters one-at-a-time around their reference values and investigated how two model outputs at the end of the simulation, capillary patency index and mean retinal thickness change, were affected (S16 Fig). Note that the mean retinal thickness change parameter map has an abscissa with a maximum of only 5%. This may seem small but this is mean retinal thickness averaged over the entire area and retinal edema was generally localized. The parameters selected are: diffusion coefficient of VEGF $D_{VEGF}^{tis},$critical blood flow velocity$u^{thr}$, critical VEGF level$m_{VEGF}^{thr}$, maximum synthesis rate of VEGF$k_{VEGF}^{prod}$, threshold of VEGF level to trigger edema$m_{VEGF}^{thrE}$, and maximum metabolic rate of oxygen$M_{0}$. All parameter variation simulations use CASE 1 parameters as a reference point denoted as $"1\times"$. Capillary patency index, calculated as fraction of patent (i.e., unoccluded) capillaries at the end of the simulation with magnitude ranging from 0% to 100%, measures the degree of progression of the capillary occlusion within the simulation. Higher value of$D_{VEGF}^{tis}$, higher value of$k_{VEGF}^{prod}$, higher value of$u^{thr}$, higher value of$M_{0}$ and lower value of$m_{VEGF}^{thr}$ result in comparably smaller capillary patency index in slightly different ways (S16A Fig). Increase of$D_{VEGF}^{tis}$ enhances the diffusing length of VEGF, while increase of$k_{VEGF}^{prod}$, on the other hand, increases synthesis rate of VEGF by Mueller cells. In contrast, increase of $u^{thr}$or decrease of$m_{VEGF}^{thr}$ raises occlusion probability given a certain blood flow velocity or VEGF level respectively. Nevertheless,$m_{VEGF}^{thrE}$ shows little effect on the patency index, because it plays no role in probabilistic judgement on capillary occlusion.

Relative thickness change is calculated literally as percentage of variation in average magnitude along Z direction of the retinal tissue at the end of simulation. A higher value of$m_{VEGF}^{thrE}$, a higher value of$D_{VEGF}^{tis}$ and a lower value of$k_{VEGF}^{prod}$ all lead to less retinal thickening (S16B Fig). Apparently, $m_{VEGF}^{thrE}$ influences retinal thickness change significantly, by directly determining how persistently edema is formed. In contrast, $D_{VEGF}^{tis}$ and$k_{VEGF}^{prod}$ control VEGF availability, how fast VEGF diffuses and how fast it is produced respectively. A minor effect is also observed from change in $u^{thr}$, $m_{VEGF}^{thr}$ and $M_{0}$, which play an indirect role in edema formation. Because occluded capillaries don’t actively leak though they may have leaked and resulted in FP in the past, rapid progression of capillary occlusion seems to decrease the amount of edema formation.

These parameter variation simulations provide more insight for some parameter selections by showing how a certain parameter *at the cellular level* would influence a model outcome *at the tissue level*. Such simulations support that the model can still produce plausible results with considerable variations of parameter values. This supports the validity of the model’s fundamental structure, an adverse feedback mechanism governing retinal capillary occlusion and also means that as future experimental data for these parameters become available the model can be refined to improve morphological accuracy and provide greater quantitative predictive value for clinical applications.

**Implementation of simulations**

All simulations were executed using open-source software Compucell3D (<http://www.compucell3d.org/> [9]. Replicate experiments were carried out on Indiana University supercomputer Big Red 2 (<https://kb.iu.edu/d/bcqt>).

**References**

1. Pries AR, Secomb TW, Gaehtgens P, Gross JF. Blood flow in microvascular networks. Experiments and simulation. Circ Res. 1990;67(4):826-34.
2. Pries AR, Secomb TW, Gessner T, Sperandio MB, Gross JF, Gaehtgens P. Resistance to blood flow in microvessels in vivo. Circ Res. 1994;75(5):904-15.
3. Pries AR, Secomb TW. Microvascular blood viscosity in vivo and the endothelial surface layer. Am J Physiol Heart Circ Physiol. 2005;289(6):H2657-64.
4. Pries AR, Secomb TW, Gaehtgens P. The endothelial surface layer. Pflugers Arch. 2000;440(5):653-66.
5. Reglin B, Secomb TW, Pries AR. Structural adaptation of microvessel diameters in response to metabolic stimuli: where are the oxygen sensors? Am J Physiol Heart Circ Physiol. 2009;297(6):H2206-19.
6. Graner F, Glazier JA. Simulation of biological cell sorting using a two-dimensional extended Potts model. Phys Rev Lett. 1992;69(13):2013-6.
7. Glazier JA, Graner F. Simulation of the differential adhesion driven rearrangement of biological cells. Phys Rev E Stat Phys Plasmas Fluids Relat Interdiscip Topics. 1993;47(3):2128-54.
8. Spitznas M, Bornfeld N. The architecture of the most peripheral retinal vessels. Albrecht Von Graefes Arch Klin Exp Ophthalmol. 1977;203(3-4):217-29.
9. Swat MH, Thomas GL, Belmonte JM, Shirinifard A, Hmeljak D, Glazier JA. Multi-scale modeling of tissues using CompuCell3D. Methods Cell Biol. 2012;110:325-66.
