# Supplementary material for: Progression of Diabetic Capillary Occlusion: A Model
Source: PLoS Comput Biol. 2016 Jun 14;12(6):e1004932. doi: 10.1371/journal.pcbi.1004932 (PMC4907516; doi:10.1371/journal.pcbi.1004932)
Supplement: S2 Text — (DOCX) [file pcbi.1004932.s023.docx]

**S2 Text: MACULAR SIMULATION WITH A DIFFERENT INITIAL CAPILLARY OCCLUSION SITE – CASE 2**

Another macular simulation with the same starting capillary network topology but a different initial capillary occlusion site is presented here for detailed comparison. In this simulation, a different capillary, which was closer to the FAZ, was initially occluded (S1 Fig). A series of new occlusion events were observed from week 76 to 96 but not afterwards, and the Arteriole and Venule flow path both remained intact (S1B-D Fig). This feature is also reflected in the oxygen and VEGF color map (S2 Fig, S3 Fig). Average oxygen tension dropped about 3 mmHg since the onset of capillary occlusion (S5A Fig), and about 20% of cells eventually turned hypoxic (S5B Fig, S6 Fig). Since the major vessels of this network were well preserved, the total flow rate was enhanced each time an occlusion occurred as a flow response compensated for the reduced oxygenation (S5C Fig). The high flow-low oxygen state in CASE 2 reflected a transition stage as depicted in Fig. 14B. Average minimal cell-to-vessel distance grew more than 50% at the end of the simulation (S5D Fig). Several edematous sites developed, within and at the edge of the ischemic region (S4 Fig).The initially occluded capillary segment in CASE 2 is another capillary whose blockage triggered propagation of occlusion with high probability as shown in the patency map of the replicate simulations (Fig. 15B). In roughly half of the simulations in which this particular capillary was initially occluded, nearby capillaries were also observed to be occluded at the end of the simulation. In addition, this occlusion seemed to have a greater impact on capillary segments at the arterial side of the network, since they had a higher frequency of derived occlusion than did those near the venous end of the capillary network. The general patterns of capillary occlusions, ischemia, and VEGF concentrations seen in the figures for CASE 2 are qualitatively similar to those for CASE 1 though the specific quantitative model outputs are different.
