# Supplementary material for: Progression of Diabetic Capillary Occlusion: A Model
Source: PLoS Comput Biol. 2016 Jun 14;12(6):e1004932. doi: 10.1371/journal.pcbi.1004932 (PMC4907516; doi:10.1371/journal.pcbi.1004932)
Supplement: S3 Text — (DOCX) [file pcbi.1004932.s024.docx]

**S3 Text: SIMULATION IN A PATTERNED HEXAGONAL CAPILLARY NETWORK**

We further explored whether similar propagation of occlusion could be observed in an artificially designed and constructed hexagonal capillary network (S8B Fig). Qualitatively the hexagonal network can reproduce the progression of occlusion as observed in CASE 1 and CASE 2. Largely the hexagonal model served to reinforce the necessity of the vascular criticality assumption as only networks of a particular size produced propagation of capillary occlusion similar to the macular network.

Structural characteristics seem to be the primary contributing factor to susceptibility within a hexagonal network. A range of hexagon diameters was explored. If the hexagon size was small relative to oxygen diffusion distances, there was very little propagation of occlusions. This would be expected as cells could remain oxygenated without VEGF production even in the presence of a capillary occlusion. Hexagonal capillary networks with small hexagon sizes thus did not meet the criticality of vascular supply condition postulated for the retina. A capillary network with too large of hexagon sizes has the opposite situation in that areas are hypoxic initially, VEGF is produced, and capillary occlusion rapidly progresses. Only for an intermediately sized hexagon network can the model of the capillary occlusion process progress similarly to that seen in the retinal capillary network. Even in this case both the density of junctions and connections obviously differ from those seen in the macular network (S8A Fig). The digitized macular network had much more densely packed branching junctions and linked capillary segments distant from the FAZ, while capillaries in the manually built hexagonal network were more uniformly patterned. As expected from the hexagonal network model, the macular network had a low tendency for propagation far from the FAZ where the capillary network is denser (Fig. 15).
