# Supplementary material for: Progression of Diabetic Capillary Occlusion: A Model
Source: PLoS Comput Biol. 2016 Jun 14;12(6):e1004932. doi: 10.1371/journal.pcbi.1004932 (PMC4907516; doi:10.1371/journal.pcbi.1004932)
Supplement: S4 Text — (DOCX) [file pcbi.1004932.s025.docx]

**S4 Text: SIMULATION ON PERIPHERAL CAPILLARY NETWORK SCHEMATIC**

S8A Fig shows a drawing of the peripheral vascular arcade after Spitznas [1]. We ran simulations on a manually created peripheral retinal capillary network schematic, structurally similar to those imaged in the retinal periphery by Spitznas [1] (S8B Fig). In this simplified schematic structure, each “tombstone”-like sector is comprised of an arteriole, a venule, a peripheral shunt vessel and several parallel capillaries in a ‘ladder’ type arrangement as termed by Spitznas [1]. As the problem of severe visual loss in diabetes is due to retinal neovascularization and as this neovascularization is generally at the posterior border of large areas of peripheral ischemia, it seemed appropriate to apply the model to vascular network architecture similar to that seen in the peripheral retina. Another important consideration is areal. The retinal periphery constitutes the large majority of total retinal area which, while not subserving visual acuity, is likely the major contributor to the total VEGF production with high vitreal VEGF levels present in diabetic neovascularization and rubeosis iridis. The model shows rapid progressive loss of a sector between an arteriole and venule through loss of capillary rungs with the development of occlusions, ischemia, and areas of elevated VEGF (S9-S11 Fig). Additionally there is limited tendency to fluid leakage and edema formation in this model of peripheral retinal capillary occlusion (S12 Fig). This is consistent with the very limited clinical tendency toward peripheral edema in diabetic retinopathy [2]. This occurs within the model largely due to the rapid progression of capillary occlusion in the peripheral capillary network once occlusion occurs. In replicative runs the capillary loss generally did not cross from one sector to another but rapidly progressive loss occurred within a sector (not shown). Two clinical observations seen commonly in fluorescein angiography are 1) the preservation of flow in the peripheral arterioles and venules despite extensive peripheral capillary dropout and 2) the apparent ‘barrier’ function of an arteriole or venule in that capillary dropout can often be extensive on one side of the vessel with a relatively intact capillary network on the other side. Both of these phenomena are seen in the model. The summary graphs of model properties with time (S13 Fig) are the simplest seen in the model. There is a simple stepwise progression over time of each of the model properties measured. In particular there is a progressive decrease of blood flow in the periphery with no period of increased flow. This results in peripheral ischemia consistent with the regional distribution of diabetic lesions emphasized by Skov Jensen [2]. In S14 Fig, the cell oxygenation fraction histogram is similar in form to those seen earlier with a transition from a unimodal to a bimodal shape over time with an increase in number of cells that are hypoxic. There is a difference from the other graphs in that no cells have the very high oxygen levels seen in the other network structures. This is a consequence of the lower flows at the lower peripheral arterio-venous pressure differences. The peripheral retinal vascular network anterior to the eye’s equator is essentially a vascular monolayer more similar to the structure of the model than is the posterior pole of the eye [1]. The situation in the periphery is distinct in that the arteriolar venular pressure difference must continue to decrease at more and more peripheral locations. This physiological situation is made ‘worse’, i.e. the difference made even less, by the specialized peripheral shunt vessel carrying blood flow from the arteriole to the venule without passage through a capillary network. Additionally it is thought that plasma skimming [3] in the more posterior vessels increases the hematocrit and therefore blood viscosity as blood travels further into the periphery. Conceptually these factors would seem to predispose to occlusion. The model does not incorporate plasma skimming but does incorporate the peripheral shunt vessels. The model shows a much greater tendency toward propagation of occlusion in the peripheral network than in the macular sector. The model shows this high propensity to propagation of capillary occlusion in the periphery as well as some vascular ‘barrier’ function of the arterioles and venules, likely due to limited VEGF diffusion distances. These features emulate those commonly but not universally seen in the clinical situation.

**References**

1. Spitznas M, Bornfeld N. The architecture of the most peripheral retinal vessels. Albrecht Von Graefes Arch Klin Exp Ophthalmol. 1977;203(3-4):217-29.
2. Skov Jensen P, Jeppesen P, Bek T. Differential diameter responses in macular and peripheral retinal arterioles may contribute to the regional distribution of diabetic retinopathy lesions. Graefes Arch Clin Exp Ophthalmol. 2011;249(3):407-12.
3. Lemmingson W. Changes in the pattern of retinal blood circulation caused by plasma skimming. Klin Monbl Augenheilkd. 1971;159(6):790-3.
